# Resonance-assisted/impaired anion–π interaction: towards the design of novel anion receptors†

**DOI:** 10.1039/d0ra07877h

**Published:** 2020-10-01

**Authors:** Juan Du, Changwei Wang, Shiwei Yin, Wenliang Wang, Yirong Mo

**Affiliations:** Key Laboratory for Macromolecular Science of Shaanxi Province, School of Chemistry & Chemical Engineering, Shaanxi Normal University Xi'an 710119 China upc.changweiwang@gmail.com yin_sw@snnu.edu.cn wlwang@gmail.com; Department of Nanoscience Joint School of Nanoscience & Nanoengineering, University of North Carolina at Greensboro Greensboro NC 27401 USA y_mo3@uncg.edu

## Abstract

Substituents alter the electron density distribution in benzene in various ways, depending on their electron withdrawing and donating capabilities, as summarized by the empirical Hammett equation. The change of the π electron density distribution subsequently impacts the interaction of substituted benzenes or other cyclic conjugated rings with anions. Currently the design and synthesis of conjugated cyclic receptors capable of binding anions is an active field due to their applications in the sensing and removal of environmental contaminants and molecular recognition. By using the block-localized wavefunction (BLW) method, which is a variant of *ab initio* valence bond (VB) theory and can derive the reference resonance-free state self-consistently, we quantified the resonance-assisted (RA) or resonance-impaired (RI) phenomena in anion–π interactions from both structural and energetic perspectives. The frozen interaction, in which the electrostatic attraction is involved, has been shown to be the governing factor for the RA or RI interactions with anions. Energy analyses based on the empirical point charge (EPC) model indicated that the anion–π interactions can be simplified as the attraction between a negative point charge (anion) and a group of local dipoles, affected by the enriched or diminished π-cloud due to the resonance between the substituents and the conjugated ring. Hence, two strategies for the design of novel anion receptors can be envisioned. One is the enhancement of the magnitudes and/or numbers of local dipoles (polarized σ bonds), and the other is the reduction of π electron density in conjugated rings. For cases with the RI characteristics, “curved” aromatic molecules are preferred to be anion receptors. Indeed, extremely strong binding was found in complexes formed with fluorinated corannulene (F-CDD) and fluorinated [5]cycloparaphenylene (F-[5]CPP). Inspired by the RA phenomenon, complexes of *p*-, *o*- and *m*-benzoquinones with halides were revisited.

## Abstract 2

Substituents alter the electron density distribution in benzene in various ways, depending on their electron withdrawing and donating capabilities, as summarized by the empirical Hammett equation.

## Graphical abstract

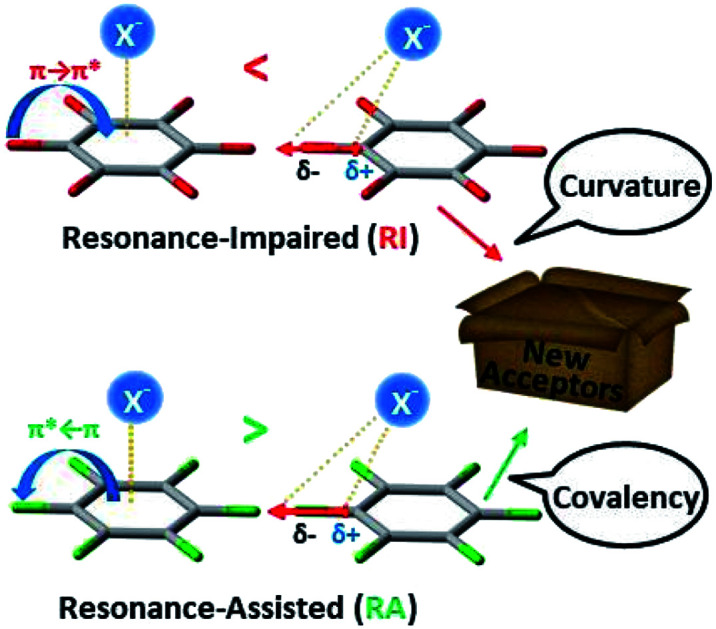

## 1. Introduction

Noncovalent interactions exist ubiquitously in all aggregates and materials, and provide us with directional “linkers” for the construction of chemical and biological substances.^1–5^ Unlike a chemical bond which involves sharing a pair of electrons and thus is strong, a noncovalent bond is usually a weak force of various natures such as electrostatic, van der Waals, polarization *etc.* The accumulation of many weak noncovalent interactions can form a strong binding force in such as proteins, nucleic acids and self-assembling materials. Based on the specific element providing the electrophilic cap for the contact, noncovalent interactions can be defined as hydrogen bonds, tetrel bonds, pnicogen bonds, chalcogen bonds, halogen bonds *etc.*, in which the electrostatic attraction stems from the positively charged hydrogen or σ-hole,^6–8^ and the π face^9–15^ or lone pair region.^16–20^ Both experimental and computational studies of noncovalent interactions get broader over the time as new unconventional types and counterintuitive models, such as the resonance-assisted and resonance-impaired hydrogen bond (RAHB and RIHB),^21–28^ and anti-electrostatic hydrogen or halogen bonds^29–31^ have been identified and proposed. These new family members of noncovalent interactions, on one hand, potentially supply us with novel elementary “glues” for the architecture of complexes. On the other hand, they also call for updates of our understanding of noncovalent interactions. One of the particular instances is the anion–π interaction, which represents the attraction between the π face of a conjugated molecule and an anion.^32–44^ This novel interaction has sparked a surge of interests,^32,33,38,40,45–50^ and been remarkably utilized in anion recognition,^38,40,46,48,50–55^ supramolecular chemistry^56–59^ and catalysis.^56–58,60–62^ From the first sight, the anion–π interaction is counterintuitive as the interaction between the π-cloud of electrons and anion is expected to be electrostatically repulsive. Nevertheless, the key role of electrostatic attraction was implied in the pioneering experimental investigations decades ago,^63–65^ and was theoretically supported in the term of quadrupole inversion (opposite sign to benzene) due to the substitution of strong electron-withdrawing groups,^66^ which turns the negative electrostatic potential (ESP) on the π-face to be positive (π-hole).^13–15,67,68^ More interestingly, the anion–π interaction indisputably is an resonance-related phenomenon,^33^ since conjugated and often aromatic rings have been broadly adopted as anion receptors. However, the exact role of resonance has been barely explored largely due to the lack of proper references. From the view of the valence bond (VB) theory, the best reference is always the major Lewis structure of any anion receptor itself with all electrons localized between two bonding atoms or individual atoms. For instance, the resonance in benzene can be best elucidated with the reference of the Kekulé structure.

Still, numerous computational investigations have been carried out to explore the nature of anion–π interactions within the molecular orbital (MO) theory or density functional theory (DFT).^11,34,39,69^ Politzer *et al.* found that the strength of anion–π interaction correlates well with the magnitude of the ESPs on the π-hole and its interacting anion.^6,11^ In other word, the anion–π interaction can be well interpreted with the electrostatic model. This is also supported by Wheeler *et al.*, who observed the good correlation between the ESPs above the ring centroids and the predicted interaction energies.^70^ Moreover, evidences for the key role of the electrostatic attraction,^71,72^ as well as the significance of the polarization effect, have also been obtained quantitatively, using a variety of energy decomposition analysis (EDA) approaches.^34,66,73–78^ Differently, the strength of anion–π interaction in the I^−^⋯C_6_F_6_ complex was determined in a combined experimental and computational investigation by Anstöter *et al.*, who found that the attraction is governed by the electron correlation. The latter accounts for about 41% of the interaction energy, with the rest from the frozen and polarization interactions.^79^ But this prominent role of the electron correlation could be compatible with the electrostatic explanation, because the destabilizing Pauli exchange repulsion is also included in the frozen term in their absolutely localized molecular orbital (ALMO) EDA analysis.^80–82^ In other words, if the Pauli repulsion is taken out of the frozen energy term, the remaining electrostatic stabilization would be comparably important as the electron correlation. Experimentally, novel anion–π associates were proposed by Kepler *et al.* in 2019, by using the *p*-benzoquinones as the halide receptors.^83^ Intriguingly, the bonding site in a *p*-benzoquinone is shifted away from the principal axis of the aromatic ring, which is deformed by the bonding with a considerable magnitude of covalency.^83^ Furthermore, the charge transfer nature was proved by means of the Natural Bond Orbital (NBO) analysis^84,85^ and Mulliken correlation.^86–88^ In another case, quinoid rings were utilized as iodide receptors in experiments in 2018, and the importance of charge transfer interactions was suggested by the color of crystals and DFT calculations.^89^ Thus, it is reasonable to conclude that all electrostatic attraction, polarization, charge transfer and dispersion (electron correlation) could be significant to specific anion–π contacts, making the anion–π interaction a diversified bonding family.

Apart from the nature of anion–π interactions, how the π electron cloud contributes to the interaction is another intriguing issue, as the understanding would allow researchers to modulate the π-cloud and consequently tune the anion–π interactions. The correlations between the bonding strength and the aromaticity criteria such as isodesmic stabilization energy and NICS^90^ of the anion receptor were found by Alkorta *et al.*, thus highlighting the significance of π resonance.^33^ However, Kozuch suggested that the “π-hole” bond is a misleading term because it originates from the σ framework.^14^ For instance, fluorine, as one of the most wildly adopted electron-withdrawing substituents on the anion receptors,^42^ is a σ-electron acceptor but π-electron donor. As a consequence, an electrostatic attraction of C_6_F_6_ with anions comes from the accumulated σ-holes of F–C bonds in the σ framework, and is actually discounted by the enrichment of π-electrons (a RI phenomenon).^14^ Furthermore, persuasive evidence against the positive contribution of π electrons was presented in the complex formed by hexafluoroborazine and chloride (B_3_N_3_F_6_⋯Cl^−^), where the conjugated ring was twisted remarkably by the anion, indicating an unfavorable role of the resonance.^14^ The negative role of π-electrons in anion–π interactions was also supported by Wheeler and Houk, who reproduced the interaction energies for 83 anion–π complexes qualitatively, using a simple charge-dipole model, in which the π-polarization effect was turned off.^70^ Similar theoretical approach has also been successfully applied in the investigation of π/π stacking.^91^

It has been a textbook knowledge that a substituent can influence the π electron distribution and subsequently affect the reaction rates at different sites of the substituted benzene ring, as summarized by the empirical Hammett equation. Elucidating the impacts of conjugation between the cyclic receptor ring and substituents could clarify the different roles of σ and π electrons and provide us a more feasible pathway to modulate the strengths and bonding sites of anion–π interactions. In this regard, the popular RAHB,^21–26^ which refers to the strengthened interplay between H-bonding and the π resonance, is an advisable example for the current work. While the RAHB has been extensively investigated and utilized,^92–112^ recently the RIHB concept has also been proposed and confirmed.^27,28^ Notably, the enhanced electrostatic attraction due to the π-resonance was theoretically proved to be the main cause of RAHB by Mo and coworkers.^98,110,113,114^ Using the block-localized wavefunction (BLW) method which can disable the π resonance,^115,116^ they showed that the frozen term, in which the electrostatic interaction is included, becomes more stabilizing for exemplary cases, when the π-conjugation is “turned on”. Moreover, both RAHB and RIHB have been systemically examined, and the key difference lies in the flowing direction of π-electrons.^117^ Analogous to RAHB/RIHB, the contribution of resonance to the anion–π interactions is also a heuristic topic, yet less explored. The critical justification of the resonance effect requires a theoretical approach that can define electron-localized (resonance or Lewis) state as reference. This can be achieved by the VB theory, where a molecular wavefunction can be defines with a linear combination of resonance states and each resonance state can be defined with the Heitler–London–Slater–Pauling function.^118–122^ The BLW method is the simplest *ab initio* VB method that combines the computational efficiency of MO theory and the chemical intuition of VB theory, and can derive the optimal electron-localized state self-consistently. Besides, the binding energy can be decomposed into several physically meaningful terms based on BLW method (called BLW-EDA).^123^ We note that there are a range of EDA schemes, including the symmetry-adapted perturbation theory (SAPT),^124^ EDA-NOCV,^125,126^ and the Kitaura and Morokuma (KM) scheme^127,128^ that have been developed and extensively applied to the studies on the nature of chemical bonds.^129^ While the nature of π-hole and σ-hole interactions can be probed with the ALMO^80–82^ as mentioned in a recent review,^12^ this method is actually the same as the BLW method.

In this work, we intended to elucidate the impacts of π resonance on the strengths of anion–π interactions, by examining the changes of the bonding strengths after quenching the π-orbital mixing between conjugated rings and substituents. The contribution of resonance to each energy component was clarified by means of the BLW-EDA approach. Both the RA and RI phenomena and the dominating role of the electrostatic interaction were further confirmed using an empirical point charges (EPC) model, by replacing the anion or all atoms of the aromatic monomer with point charge(s). Key factors for the “π-hole” were illuminated by inspecting the evolution of ESP along the bonding direction. Finally, promising anion receptors were proposed based on our updated understanding of resonance in anion–π interactions.

## 2. Methodology and computational details

### 2.1 Block-localized wave function (BLW) method

MO theory is featured by Slater determinants composed of delocalized and orthogonal MOs, which lead to the high computational efficiency. In contrast, VB theory uses localized and nonorthogonal orbitals to construct HLSP functions and each HLSP function is a linear combination of 2^*n*^ (*n* is the number of chemical bonds) Slater determinants. While the computational cost is much high in *ab initio* VB theory, resonance can be quantified with the energy difference between the ground state and the most stable VB structure. The essence of the BLW method lies in the partition of the whole molecule into several functional groups (fragments or blocks), and all orbitals are block-localized. In other words, orbitals are expanded only in one block. Orbitals belonging to the same block are constrained to be orthogonal (a MO characteristics), but orbitals belonging to different blocks are non-orthogonal (a VB characteristics). The final electron-localized state is defined with one Slater determinant composed of block-localized MOs that are self-consistently optimized. In this way, the BLW method is the simplest variant of VB theory, but it retains the high computational efficiency of MO theory.

In this work, the diabatic state, in which the π electrons of the substituents on the ring are strictly localized, is expressed as1
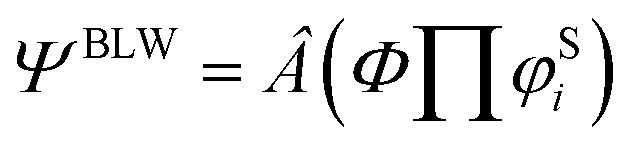
where {*φ*^S^_*i*_} corresponds to the occupied π orbitals of substituents, and *Φ* denotes the Hartree product of all remaining orbitals. Geometrical optimization and vibrational frequency calculations of diabatic states are available at the Hartree–Fock (HF) and DFT levels.^116^

### 2.2 BLW energy decomposition (BLW-ED) analysis

In the BLW-ED analyses, both the anion and its receptor can be conveniently treated as two blocks respectively, and their binding energy (Δ*E*_b_) can be decomposed into five physically meaningful terms, including deformation (Δ*E*_def_), frozen (Δ*E*_F_), polarization (Δ*E*_pol_), charge transfer (Δ*E*_CT_), and dispersion correction (Δ*E*_disp_) energies as

2 Δ*E*_b_ = Δ*E*_def_ + Δ*E*_F_ + Δ*E*_pol_ + Δ*E*_CT_ + Δ*E*_disp_ = Δ*E*_def_ + Δ*E*_int_

where Δ*E*_int_ is the energy change from the distorted and infinitely separated monomers to the formation of complex. The basis set superposition error (BSSE) correction is computed using the counterpoise method of Boys and Bernardi,^130^ and included in the charge transfer energy as only in this steps, orbitals are expanded to the whole system. The dispersion correction is assessed with Grimme's dispersion correction (D3) approach^131,132^ and defined as the difference between the complex and the sum of distorted monomers, *e.g.*

3 Δ*E*_disp_ = *E*^DFT^_disp_ − *E*^A^_disp_ − *E*^B^_disp_

We note that the electron correlation itself is not an interaction term, as it has been largely included in the frozen term when DFT is adopted.

### 2.3 Empirical point charges (EPC) model

To inspect the role of electrostatic attraction explicitly, the EPC model was proposed to evaluate the interaction energy with an anion (halide in this work) replaced by a point charge (PC). Obviously, resonance within the receptor still contributes in this model. The interaction energy between the receptor and the PC can decomposed into electrostatic and polarization terms:

4 Δ*E*_int_ = *E*(*M*^Pol^ + PC) − *E*(*M*^0^) − *E*(PC) = [*E*(*M*^0^ + PC) − *E*(*M*^0^) − *E*(PC)] + [*E*(*M*^Pol^ + PC) − *E*(*M*^0^ + PC)] = Δ*E*_eles_ + Δ*E*_pol_

where *E*(*M*^Pol^ + PC) is the total energy of the complex of the polarized receptor with the PC, *E*(*M*^0^) and *E*(PC) represent the energies of the free and distorted receptor and the PC of certain arrangement, respectively. *E*(*M*^0^ + PC) denotes the energy of system constructed by the distorted yet unpolarized receptor and the PC. Δ*E*_eles_ are Δ*E*_pol_ are the electrostatic interaction and the polarization energy, respectively. Since there is π resonance allowed in the receptor in computations, this EPC scheme is thus called resonance contributing (RC) scheme.

The interaction energy can be alternatively computed with another scheme, by replacing each atom of the aromatic ring with a PC. In this case, the resonance is absent, as the PC values were derived from the diabatic wavefunction with the Natural Population Analysis (NPA).^133^ Only the electrostatic and polarization interactions between the anion, and a group of local-dipoles are left in this scheme, which thus is the simplest resonance free (RF) scheme.

## 3. Results and discussion

**Scheme 1:**
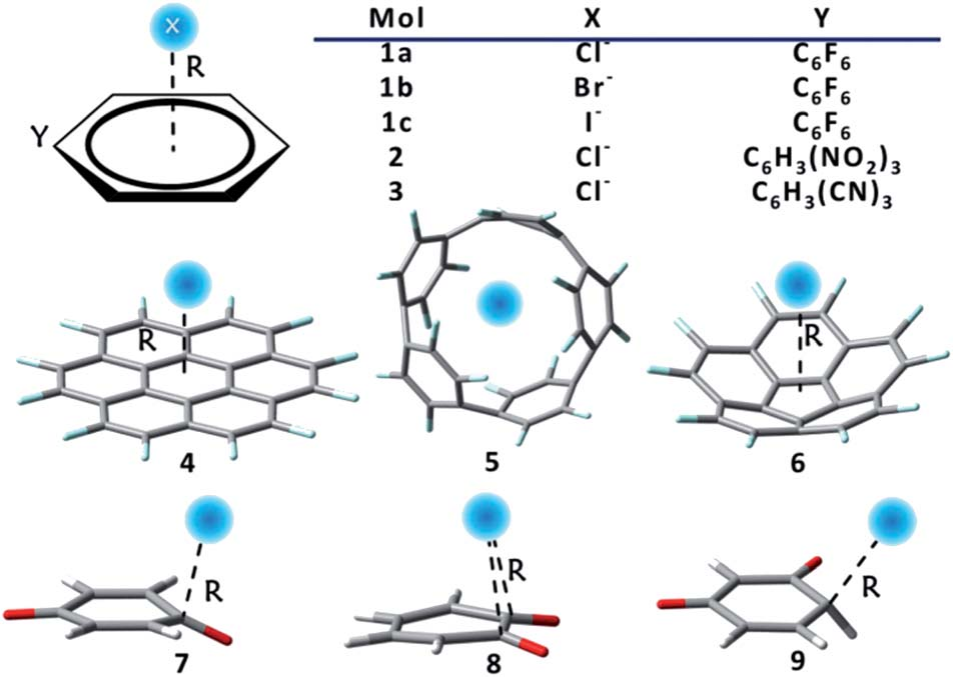
Complexes studied in this work.

Two groups of complexes (Scheme 1) were studied in this work, including the extensively investigated prototypes, or substituted benzenes as receptors for halide ions, which form group 1, and systems with macrocyclic or new anion receptors which form group 2. The latter are constructed according to our improved understanding of RI and RA features. Numbers in Scheme 1 denote different anion receptors, and a, b or c stands for Cl^−^, Br^−^ or I^−^ respectively. Group 1 consists of complexes 1, 2 and 3, where hexafluorobenzene, 1,3,5-trinitrobenzene, and 1,3,5-tricyanobenzene were used as anion receptors where fluorine is the only π-electron-donating substituent. Group 2 is composed of complexes 4–9. The M062X functional^134^ was employed, as it has been proved to be a reliable method for non-covalent interactions.^135–137^ Computations were improved with the Grimme's dispersion correction.^131,132^ Pivotal observations were also confirmed using different functionals, and HF method as well. Full geometry optimizations were carried out, and all optimal structures were proved to be actual minima based on the analyses of harmonic frequencies for all complexes using the GAMESS (US) program.^138^ The cc-pVDZ basis set was adopted in the optimization and frequency calculations of group 2 species, but the cc-pVTZ basis set was used for all the rest calculations. Specifically, the small-core relativistic pseudopotential basis set cc-pVDZ-PP or cc-pVTZ-PP^139,140^ were employed for iodine. For systems in group 1, geometry optimizations and vibrational frequency calculations were also carried out using the spin component-scaled second-order Møller–Plesset perturbation theory (SCS-MP2),^141–145^ together with different DFTs for benchmarks, as shown in Table S1.† Sequences of binding strengths obtained with all DFTs remain unchanged, and consistent with the results at the SCS-MP2/aug-cc-pVTZ (-PP for iodine) level. Intriguingly, the overall attractions and the order of binding energies are also reproduced at the HF level, in spite of the underestimation of the binding strengths due to the missing of the electron correlations. Notably, the binding energy in complex 1c is −50.8 kJ mol^−1^ using M062X-D3, only 0.7 kJ mol^−1^ lower than the value observed by Anstöter *et al.* in their combined spectral and state-of-the-art computational investigation.^79^ It should be noted, however, that the analyses of group 1 were carried out at the geometries obtained from constrained optimizations, with the anion receptors constrained to planar, aiming at the rigorous separation of the π-subspace. This constraint is actually insignificant as the reduction in binding energies is less than 1 kJ mol^−1^ (Table S2†).

We first examined the impacts of π resonance between the substituent groups and the central benzene ring on the binding distances and energies with the halide anions in group 1. Table 1 compiles the bond distances (*R*) and energy components computed at optimal geometries of both the localized (BLW with the resonance from the substituent groups “turned off”) and delocalized (regular DFT with the resonance from the substituent groups “turned on”) states. According to the regular DFT results, frozen energy, in which the electrostatic interaction is included, is the key player, and polarization is the second important stabilizing factor, with both charge transfer and dispersion contribute slightly to the overall attraction. Bond strength decreases as the halide anion grows heavier (1a > 1b > 1c), mainly due to the reduced stabilizing frozen and polarization interactions, along with the elongated binding distances. Both the electrostatics and polarization decay fast with the stretching of the binding distance. Notably, both 1,3,5-trinitrobenzene (2), and 1,3,5-tricyanobenzene (3), with π-electron withdrawing substituents, attract chloride more intensely than hexafluorobenzene (1a), in which the fluorine, conversely, tends to enrich the π-cloud. The enhanced attraction in complexes 2 and 3 mainly stems from the frozen energy term, implying a possible RA characteristic in complexes 2 and 3, in contrast to the RI feature in complex 1a.

**Table 1:** Key geometrical factor (Å) and energy components (kJ mol^−1^) computed at the optimal geometries of the electron-delocalized states (DFT) and the electron-localized states (BLW)

Complex	*R*	Δ*E*_def_	Δ*E*_F_	Δ*E*_pol_	Δ*E*_CT_	Δ*E*_disp_	Δ*E*_int_	Δ*E*_b_
**DFT**								
1a	3.107	0.8	−36.2	−23.3	−3.5	−0.1	−63.2	−62.3
1b	3.301	0.6	−33.3	−18.7	−4.2	−0.1	−56.4	−55.8
1c	3.523	0.5	−29.8	−15.4	−5.5	−0.1	−50.8	−50.3
2	2.604	1.5	−67.4	−31.3	−5.7	−0.4	−104.8	−103.3
3	2.503	0.2	−54.9	−30.9	−2.6	−0.4	−88.8	−88.6

**BLW**								
1a	3.045	0.0	−56.6	−23.7	−4.4	−0.1	−84.9	−84.8
1b	3.234	0.1	−50.5	−19.3	−5.1	−0.1	−75.0	−74.9
1c	3.453	0.2	−44.3	−16.1	−6.6	−0.1	−67.1	−66.9
2	3.081	0.9	−59.6	−27.1	−3.2	−0.5	−90.4	−89.5
3	3.093	0.9	−53.2	−27.7	−1.8	−0.4	−83.1	−82.2

The most significant finding from Table 1 comes from the comparison of BLW and DFT states, which is the direct evidence for the RA and RI phenomena. With the deactivation of the π conjugation from the halogen atoms to the benzene rings, the binding energy of hexahalobenzenes (1a, 1b and 1c) with chloride increases by 33–36% with the shortening of the bonding distances by 0.06–0.07 Å. Energy decomposition analyses (Table 1) show that the energy changes are solely due to the increasing electrostatic attraction (from the frozen energy term), with polarization, charge transfer and dispersion energy components barely changed from the DFT to the BLW states. This is a strong evidence for the RI phenomenon where resonance weakens the binding to anions, and in accord with the understanding that halogen atoms are π electron donors, though they (particularly fluorine) can draw electrons *via* σ induction. In contrast, both nitro and cyano groups are π electron acceptors. With the deactivation of their conjugation with benzene rings, the binding with chloride in 2 and 3 notably weakens with the obvious elongation of the bonding distances. Thus, 2 and 3 are the examples for the RA phenomenon where resonance enhances the binding to anions. The study of group 1 reveals a role of thumb for modulating the anion–π interactions, *i.e.*, increasing the π electron density in the conjugated ring would reduce the capability of binding anions, whereas removing the π electron density in the conjugated ring would enhance the π-hole and increase the capability of binding anions. The difference in flowing direction of π-electrons in 1–3 was also supported by the hyperconjugation evaluated with the NBO method (Table S3†).

As Table 1 shows that the RI or RA phenomena is solely reflected in the frozen energy term which consists of electrostatic, Pauli repulsion and DFT electron correlation, we further examined the characteristic of the frozen energy term using different functionals together with the HF method (Table 2). While it is obvious that different functionals result in different values for the frozen energy term since the electron correlation is considered at different magnitudes, it is interesting to note that the ΔΔ*E*_F_ term, which stands for the change of the frozen energy from the diabatic (BLW) to the adiabatic (DFT) state, is rather stable with different methods. Alternatively, we also evaluated the frozen energy term using a strictly localized model, in which not only the π electrons of all substituents are isolated, but the six π electrons are also strictly localized between two adjacent carbons on the 6-membered ring (*e.g.*, the Kekulé structure). It turned out that the frozen energy is almost invariant by this further localization scheme (Table S4†), which means that the anion–π interaction is insensitive to the resonance within the aromatic ring.

**Table 2:** Frozen energies (in kJ mol^−1^) in the localized (BLW) and delocalized (DFT) states, and the corresponding differences (ΔΔ*E*_F_ = DFT − BLW, in kJ mol^−1^) calculated using different methods with cc-pVTZ basis set

Complex	States	M062X	B3LYP	CAMB3LYP	ωB97X	HF
1a	BLW	−56.6	−36.0	−43.5	−52.6	−31.2
	DFT	−36.2	−18.1	−24.3	−31.4	−14.7
	ΔΔ*E*_F_	20.4	17.9	19.2	21.2	16.5
1b	BLW	−50.5	−29.4	−36.6	−46.4	−24.9
	DFT	−33.3	−14.4	−20.4	−28.8	−11.5
	ΔΔ*E*_F_	17.2	15.0	16.2	17.6	13.4
1c	BLW	−44.3	−22.7	−29.7	−39.5	−18.4
	DFT	−29.8	−10.7	−16.6	−24.8	−7.6
	ΔΔ*E*_F_	14.5	12.0	13.1	14.7	10.8
2	BLW	−59.6	−35.0	−43.7	−55.2	−40.4
	DFT	−67.4	−43.6	−51.3	−62.1	−45.7
	ΔΔ*E*_F_	−7.8	−8.6	−7.6	−6.9	−5.3
3	BLW	−53.2	−32.2	−39.1	−50.9	−27.9
	DFT	−54.9	−34.1	−41.1	−52.7	−29.4
	ΔΔ*E*_F_	−1.7	−1.9	−2.0	−1.8	−1.5

**Fig. 1:**
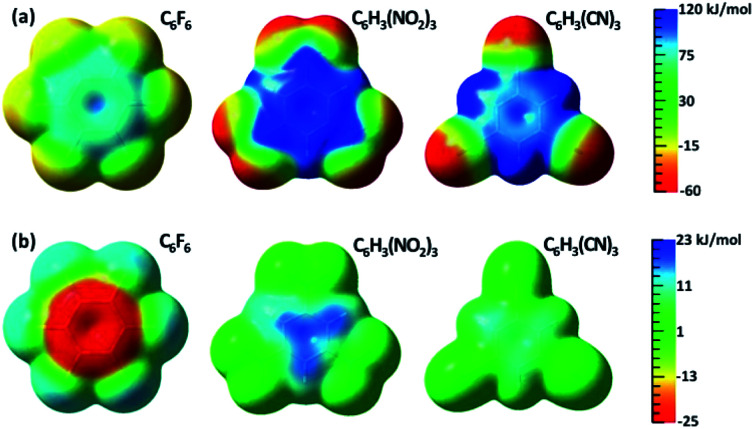
(a) ESP maps of free anion receptors calculated with DFT; (b) differences in ESP between the electron adiabatic (DFT) and diabatic (BLW) states mapped on an isodensity surface (0.001 eÅ^−3^).

The changes of electrostatic interactions involved in the frozen energy term due to the resonance from the substituent groups to the aromatic benzene ring can be intuitively demonstrated using the variations in ESPs as shown in Fig. 1b, with the ESPs maps of the adiabatic (DFT) states as references in Fig. 1a. π-Holes in the aromatic rings were found in all substituted benzenes of group 1. Importantly, the most significant variation in ESP was observed in C_6_F_6_, which is turned to be less positive due to π electron movement from fluorine atoms to the central ring. Differently, the ESP of 1,3,5-trinitrobenzene becomes much more positive, and the π-hole in 1,3,5-tricyanobenzene is slightly enhanced by the π resonance as well.

**Fig. 2:**
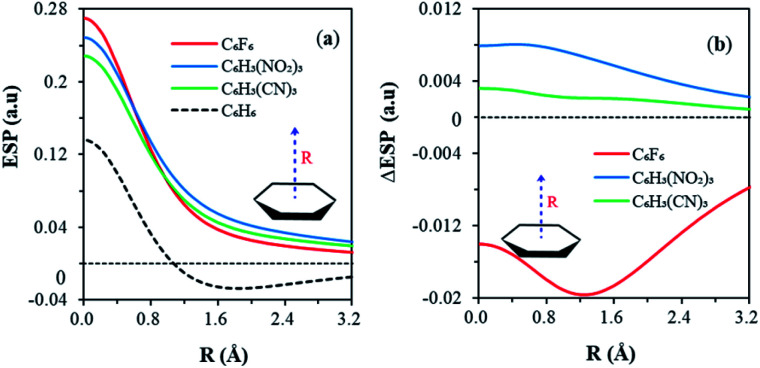
(a) Variations of ESP (in au) in the electron delocalized states of anion receptors 1a, 2, 3 and benzene, along the vertical distance (*R* in Å) above the centroid, and (b) variations of ESP due to resonance along the bonding direction.

Fig. 2 shows the variations of ESP along the bonding direction in order to help understand and examine the π-hole concept. Maximum ESP values (positive) are observed in the centroid of each aromatic ring, in the order of C_6_F_6_ > C_6_H_3_(NO_2_)_3_ > C_6_H_3_(CN)_3_ (Fig. 2a). All ESPs decrease monotonically along the bonding direction and stay above zero for all anion receptors, forming the π-hole. Specifically, the C_6_F_6_ curve goes down the most steeply, because its π-cloud is enriched by fluorine atoms and results in an extra shielding on the centroid. For comparison, the evolution of the ESP of benzene was also displayed together in Fig. 2a. Similar decreasing trend was observed beginning at the centroid. Notably, ESP falls into the negative region at the distance of about 1 Å from the centroid, in sharp contrast to other substituted benzenes. This comes from the different electronegativities of hydrogen and halogens. For each C–X (X = F, Cl, I) σ bond, there are σ holes on the two ends of the bond. Thus, in the hexahalobenzene, the ring centroid is the merging point of six σ holes due to the six C–X σ bonds. But for C–H bonds in benzene, such σ holes are less obvious. At last, all ESPs tend to converge to zero in long range, making the whole curve of benzene non-monotone. Therefore, the key difference in ESP between benzene and anion receptors is ruled by the ring center, where the σ-holes merge. The different ESP distributions in the RA and RI phenomena can also be directly exhibited by the variations of ESP along the bonding direction due to the π resonance, as shown in Fig. 2b. The C_6_F_6_ curve with the RI characteristics lies below the horizontal axis, while curves of both C_6_H_3_(NO_2_)_3_ and C_6_H_3_(CN)_3_ with the RA characteristics stay above zero all the way. The variations of ESP decrease monotonously, and approach zero gradually for C_6_H_3_(NO_2_)_3_ and C_6_H_3_(CN)_3_. Differently, a minimum point was found on the C_6_F_6_ curve, implying an additional shielding of ESP on the centroid, possibly caused by the π-electrons donated by fluorine atoms.

To further explore the role of π resonance in π–anion interactions, we first simplified the chloride anion with a point charge and used this empirical point charge (EPC) model to perform energy decomposition analyses in the cases where π resonance in receptors between substituents and the benzene ring is allowed (resonance contributing or RC scheme). Table 3 summarized the results. The comparison of Tables 1 and 3 show that the interaction energies can be well reproduced by simply replacing the anion with a point charge (RC scheme), with a relative error less than 7% at the M062X-D3 level. The electrostatic interactions are more stabilizing than the polarization interactions in the RC scheme, which is consistent with the BLW-EDA results in Table 1. In addition, the estimated electrostatic energy in Table 3 is very close to the frozen energy derived from the BLW-ED calculation in Table 1, with a difference less than 5.0 kJ mol^−1^. The similarity between the electrostatic energies in Table 3 and the frozen energies in Table 1 strongly suggests that the Pauli exchange repulsion and the electron correlation components in the frozen energy term are either both insignificant or roughly offset each other.

**Table 3:** Energy decomposition analysis (in kJ mol^−1^) using the EPC model with the M062X-D3 method

Complex	RF scheme			RC scheme		
	Δ*E*_eles_	Δ*E*_pol_	Δ*E*_int_	Δ*E*_eles_	Δ*E*_pol_	Δ*E*_int_
1a	−133.7	−7.1	−140.9	−36.1	−28.6	−64.7
1b	−117.4	−8.6	−126.0	−32.2	−23.4	−55.6
1c	−101.7	−9.5	−111.2	−28.5	−18.8	−47.3
2	−70.5	−0.6	−71.1	−72.0	−37.1	−109.2
3	0.2	−0.2	0.0	−56.3	−37.3	−93.6

We continued to simplify each atom of the anion receptor with a PC whose value is gotten from the NPA calculations of the diabatic (BLW) state. The subsequent simplified EPC model is the resonance free (RF) scheme. Compared with the above RC scheme, interaction energies in the RF scheme increase in the complexes of hexahalobenzenes (RI cases), while decrease in complexes 2 and 3 (RA cases). In addition, the differences among interaction energies are largely governed by the electrostatics, which is consistent with the RA and RI features evidenced by the above ESP analyses. Interestingly, all energy components are close to zero in the RF scheme of complex 3, which shows the most significant RA phenomenon among all complexes. However, it should be emphasized that this very crude EPC model is just a simplified approach for understanding the role of electrostatic and polarization interactions, not an accurate computational method.

The above EPC model simplified the anion–π interaction as an attraction between a negative point charge (anion) and a group of local dipoles (C–F σ bonds for example), which is further influenced by resonance. Consequently, two strategies to strengthen the anion–π interaction can be envisioned. One is to increase the magnitudes and/or numbers of local dipoles,^146^ and the other is to either diminish the π electron delocalization from substituents to the ring or enhance the π delocalization from the ring to substituents. For the first strategy, perfluorocoronene^147,148^ (complex 4) is an appropriate candidate, since the number of C–F bonds is doubled compared with C_6_F_6_. Indeed, a considerable increment (68–77%) in binding energies of perfluorocoronene with halide anions can be found by comparing 4 in Table 4 with 1 in Table 1. Meanwhile, fluorinated [5]cycloparaphenylene (F-[5]CPP, complex 5), as one of the smallest nanohoop with the suitable size for halides, has also been tested. The binding energy of complex 5a reaches −205.5 kJ mol^−1^, or more than two times higher than the binding strength in 1a. Analogous to the complexes with C_6_F_6_, the overall attractions in all complexes constructed with perfluorocoronene and F-[5]CPP are dominated by the frozen energy term, and considerably strengthened by the polarization interaction, with slight contributions from both charge transfer and dispersion interactions. The electrostatics nature revealed by the BLW-EDA results is supported by the ESPs shown in Fig. S1 (see ESI†). In addition, a binding site outside the hoop of F-[5]CPP was tested (Fig. S2†) and proved to be less attractive than the centroid by the BLW-EDA results (Table S5†).

**Table 4:** Energy contributions to the formation of optimal structures at M06-2X-D3/cc-pVDZ level of theory using the BLW energy decomposition analysis at M06-2X-D3/cc-pVTZ level (kJ mol^−1^)

	Δ*E*_def_	Δ*E*_F_	Δ*E*_pol_	Δ*E*_CT_	Δ*E*_disp_	Δ*E*_int_	Δ*E*_b_
4a	1.5	−48.6	−48.2	−8.6	−1.0	−106.3	−104.9
4b	1.4	−48.5	−39.5	−8.6	−1.0	−97.6	−96.2
4c	1.2	−49.2	−31.8	−8.3	−1.0	−90.3	−89.1
5a	5.7	−119	−85.9	−5.7	−0.7	−211.2	−205.5
5b	10.7	−92.5	−77.9	−16.8	−0.6	−187.8	−177.2
5c	31.7	−65.6	−66.8	−23.9	−0.5	−156.8	−125.2
6a	4.1	−78.3	−48.3	−4.0	−0.5	−131.1	−127.0
6b	3.4	−74.2	−40.8	−5.9	−0.5	−121.4	−118.0
6c	2.1	−69.3	−33.8	−8.1	−0.4	−111.7	−109.6
7a	15.4	−4.4	−36.3	−46.2	−0.2	−87.0	−71.6
7b	13.2	−8.7	−26.5	−37.8	−0.2	−73.3	−60.0
7c	12.1	−9.6	−20.5	−34.0	−0.2	−64.3	−52.2
8a	10.6	3.6	−36.2	−54.2	−0.2	−86.9	−76.3
8b	9.8	0.5	−26.8	−47.4	−0.2	−73.8	−64.0
8c	9.5	−0.4	−20.9	−44.3	−0.1	−65.8	−56.3
9a	124.9	394.4	−380.2	−451.1	−0.2	−437.1	−312.2
9b	110.8	346.0	−288.3	−442.7	−0.2	−385.2	−274.4
9c	99.7	293.5	−195.0	−447.1	−0.2	−348.8	−249.1

The second strategy, in which the resonance from the substituent groups is reduced by curving the conjugated structure, was testified by exploring the anion–π complexes formed with fluorinated corannulene (F-CEE, complex 6). This was inspired by an excellent review by Haupt and Lentz, who summarized the experimental investigations of modified corannulene with electron-withdrawing substituents.^149^

Obviously, F-CEE could be one ideal protype of anion receptor because of three facts. Firstly, a positive ESP is set on both the concave and convex sides, mainly by the fluoro-substitutions (Fig. S1†). Secondly, the electron density is unequally distributed on two sides, because its bowl shape leads to a more positively charged concave side. At last, the delocalization of π electrons on fluorine can be impaired by the curvature, leading to an inhibition of the RI phenomenon. Table 4 shows that the anion–π bonding is doubly strengthened by replacing C_6_F_6_ (1) with F-CEE (6). Most importantly, the average contribution from each fluoro-substitution to the binding energy is the highest among all cases tested in this work, suggesting an extra stability gained by curving. The hypothetic flat structure of F-CEE (Fig. S2†), in which both the inhibition of RI phenomenon and the electrophilicity caused by curvity are absent, was also tried for comparison. Computations showed that and the binding of the flat F-CEE with anions is significantly reduced (about 40 kJ mol^−1^ shown in Table S5†). The binding on the convex side of 6 was also examined, and the binding energy is even lower than the coplanar structure (Table S5†). There could be more modified bucky bowls, nano hoops and even nano tubes as candidates for anion receptors for our future studies.

The RA phenomenon also reminds us to re-examine substituents with strong π-electron withdrawing capabilities. Notably, Kozuch theoretically proposed a genuine and extreme receptor, cyclohexanehexone.^14^ Rosokha *et al.* conducted experimental investigation of complexes formed with halogenated *p*-benzoquinones.^83^ Considering that benzoquinones can be good candidates for testing since the oxygen attracts both σ and π electrons strongly, here we studied *p*-, *o*- and *m*-benzoquinones (complexes 7–9 see Scheme 1). For complexes formed with *p*-benzoquinone (7a, 7b and 7c), enhanced binding can be observed compared with systems formed with C_6_F_6_, but the binding sites are shifted off the principal axis, forming the Meisenheimer structures.^150^ Surprisingly, the overall attraction turns out to be dominated by the charge transfer interaction, and strengthened by remarkable polarization interactions, indicating a characteristic of covalency. Both the charge transfer and the binding energies get higher from *p*-benzoquinone to *o*-benzoquinone (complexes 8a, 8b and 8c), with the frozen energy becoming repulsive obviously due to the increasing Pauli exchange repulsion. The latter is reflected by the shortened bonding distances in the complexes of *o*-benzoquinone with halides, where halides hang on the midpoints of substituted carbons. In contrast to both *p*- and *o*-benzoquinones, *m*-benzoquinone can form a covalent bond with a halide. As listed in Table 4, this is evidenced by the short C–X distances (for comparison, typical covalent bond lengths are 1.77, 1.94 and 2.14 Å for C–Cl, C–Br and C–I bonds) and the very high but offsetting frozen and charge transfer energy terms.

## 4. Conclusions

In this work, we explored how π resonance in anion receptors influences anion–π interactions, as it has been known that substituents to a conjugated cyclic ring alter its binding to anions. Based on the BLW method which can derive electron-localized diabatic states self-consistently, we demonstrated that the deactivation of the π resonance between substituents and the benzene ring can either enhance the binding to anions (a resonance-inhibited or RI phenomenon when substituents are π electron donors such as halogens), or inhibit the binding to anions (a resonance-assisted or RA phenomenon when substituents are π electron acceptors such as nitro and cyano). The subsequent energy decomposition analyses showed that the governing factor is the frozen energy term, in which the electrostatic interaction is involved, while all the other energy components are insensitive to resonance. This governing role of the frozen energy was also confirmed using different theoretical methods. The differences between the RA and RI characteristics are not only reflected by their different electrostatic interactions included in the frozen term, but also exhibited by the differences in ESP maps between the adiabatic and diabatic states visually: the π-hole is diminished by resonance in anion receptors with π electron-donating substituents, but strengthened in aromatic rings with π electron-withdrawing groups.

The anion–π interaction was further simplified as an attraction between a negative point charge (anion) and a group of local dipoles, affected by the enriched/diminished π-cloud due to resonance, using an empirical point charge model. While in the resonance contributing or RC scheme, the interaction energies at DFT level can be well reproduced by replacing the anion with a point charge, the interaction energies turn to be more attractive in RI cases but less stabilizing in RA complexes in the resonance free or RF scheme, in which each atom of the receptor is replaced with a point charge.

Based on the BLW and EPC computations, we hypothesized that a better anion receptor can be designed with increased magnitudes and/or number of local dipoles and reduced π electron density in the conjugated ring. To this end, we tested perfluorocoronene with more local dipoles compared with the prototypical hexafluorobenzene, and demonstrated its remarkably enhanced binding to anions. Nevertheless, increasing the number of fluorine groups as substituents in perfluorocoronene also enhances the RI phenomenon at the same time, and this can be confirmed by the reduced binding energy per fluorine in perfluorocoronene compared with the value in hexafluorobenzene. A solution for this dilemma is to bend the cyclic receptor to impair the π conjugation from the substituents to the cyclic ring. Both fluorinated [5]cycloparaphenylene (F-[5]CPP) and fluorinated corannulene (F-CEE) are “curved” aromatic receptors, and computations show their remarkably enhanced binding capabilities for anion. For instance, the average binding energy in F-CEE contributed by each fluorine is increased by about 20%, compared with C_6_F_6_. Finally, *p*-, *o*- and *m*-benzoquinones were all revisited to confirm the RA phenomenon. Different from the rest cases studied in this work, the anion–π interactions with benzoquinones show considerable magnitude of covalency with binding sites shifting away from the ring centers.

## Conflicts of interest

There are no conflicts to declare.

## Supplementary Material

RA-010-D0RA07877H-s001
