# Supplementary material for: Resonance-assisted/impaired anion–π interaction: towards the design of novel anion receptors
Source: RSC Adv. 2020 Oct 1;10(59):36181–91. doi: 10.1039/d0ra07877h (PMC9056982; doi:10.1039/d0ra07877h)
Supplement: RA-010-D0RA07877H-s001 [file RA-010-D0RA07877H-s001.pdf]

This journal is © The Royal Society of Chemistry 2020

1. Additional Figures 1

Figure S1. ESP of free and optimized anion acceptors in group 2 mapped on an isodensity surface (0.001 eÅ^{-3}). 1

Figure S2. Different bonding sites tested for complexes in group 2. 2

2. Additional Tables....................2

Table S1. Binding energies (kJ/mol) and key geometrical parameter (Å) of fully optimized geometries using different methods with cc-pVTZ basis set. 3

Table S2. Differences in key geometrical factor and energy components, between the constrained optimization with the ring planished and fully optimized geometries and using M062X-D3 with cc-pVTZ (-PP) basis set. 3

Table S3. NBO analysis of restricted optimized monomers. 3

Table S4. Frozen energies (in kJ/mol) evaluated with the Lewis structure using different methods with cc-pVTZ basis set at the optimal geometries of the electron localized state (BLW state). 4

Table S5. Energy decomposition results (in kJ/mol) of complexes in group with different binding sites. 4

Table S6. Cartesian coordinate (fully optimized geometries at M062x-D3/cc-pVTZ level of theory) and charges (NPA results of the Lewis structure) for the EPC model analysis. 4

3. The xyz coordinates of complexes and monomers optimized at M062x-D3/cc-pVTZ level of theory (in Å). 7

4. The xyz coordinates of complexes and monomers optimized at M062x-D3/cc-pVDZ level of theory (in Å). 21

5. The xyz coordinates of complexes and monomers optimized at M062X-D3/cc-pVTZ level of theory (in Å) with the arometic ring planished....................52

6. The xyz coordinates of complexs optimized at BLW(M062x-D3)/cc-pVTZ level of theory with the π electrons on Fluorine atoms localized (in Å). 60

7. The xyz coordinates of complexes and monomers optimized at BLW(M062x-D3)/cc-pVTZ level of theory with the π electrons on fluorine atoms and C-C bonds both localized (in Å). 65

8. The xyz coordinates of complexes and monomers optimized at B3LYP-D3/cc-pVTZ level of theory (in Å). 70

9. The xyz coordinates of all complexes and monomers optimized at CAMB3LYP-D3/cc-pVTZ level of theory (in Å)....................74

10. The xyz coordinates of complexes and monomers optimized at HF/cc-pVTZ level of theory (in Å). .79

11. The xyz coordinates of all complexes and monomers optimized at ωB97X/cc-pVTZ level of theory (in Å). 84

12. The xyz coordinates of complexes and monomers optimized at SCSMP2/cc-pVTZ level of theory (in Å). 89

1. Additional Figures

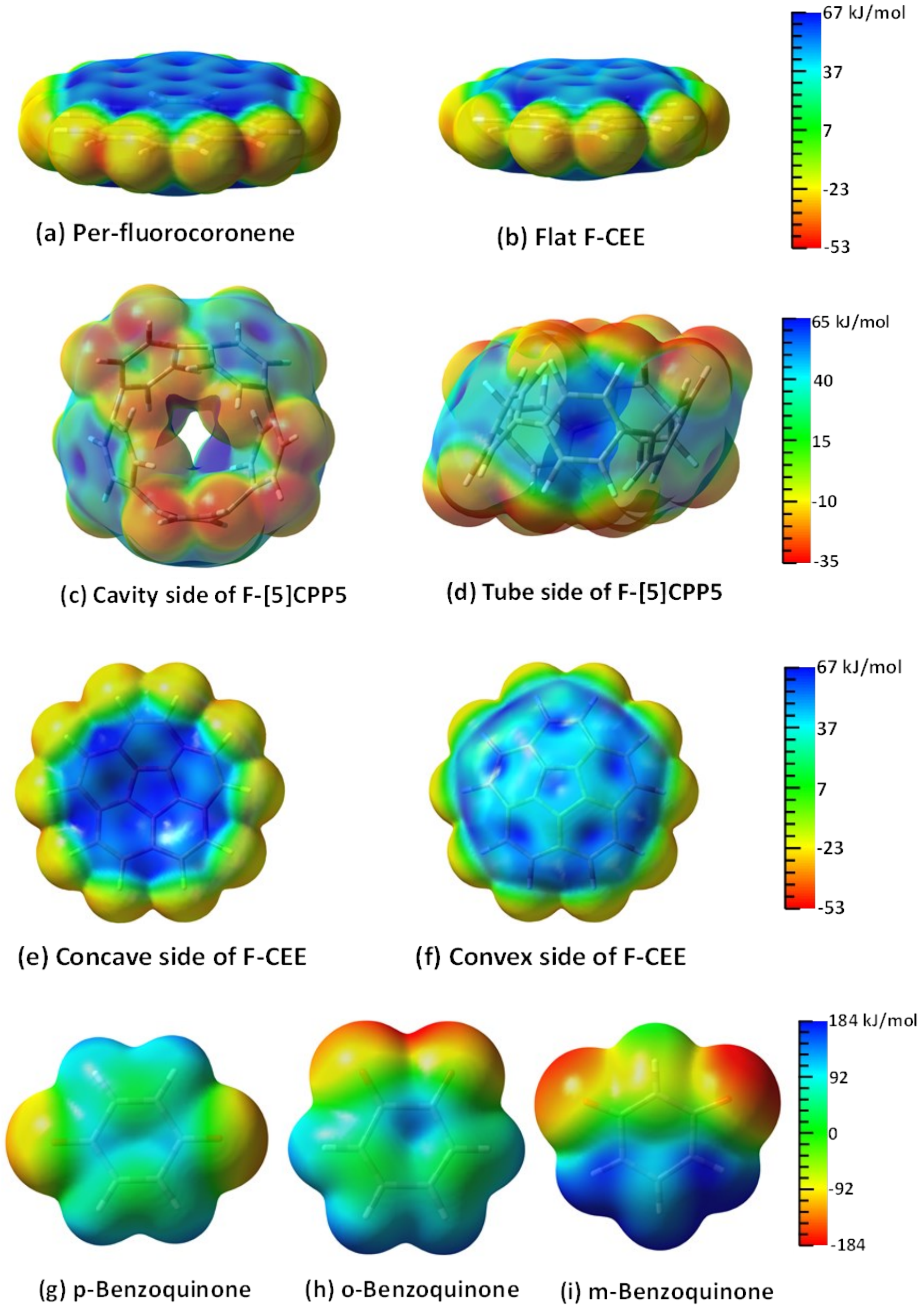

Figure S1. ESP of free and optimized anion acceptors in group 2 mapped on an isodensity surface (0.001 eÅ^{-3}).

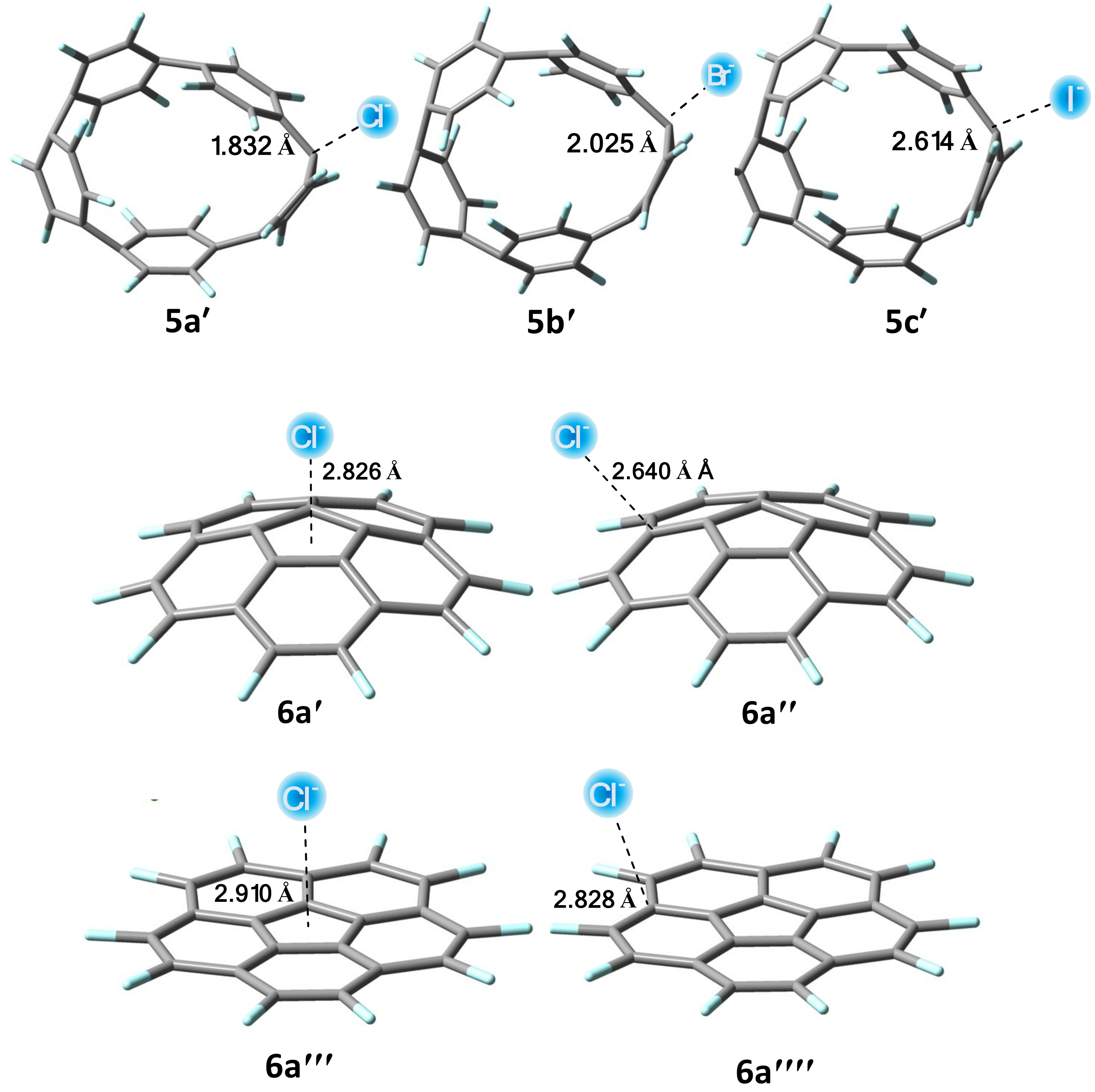

Figure S2. Different bonding sites tested for complexes in group 2.

2. Additional Tables

Table S1. Binding energies (kJ/mol) and key geometrical parameter (Å) of fully optimized geometries using different methods with cc-pVTZ basis set.

	SCSMP2		M062X-D3		B3LYP		CAMB3LYP		ωB97X		HF	
	ΔE_b	R	ΔE_b	R	ΔE_b	R	ΔE_b	R	ΔE_b	R	ΔE_b	R
1a	-52.4	3.115	-62.5	3.078	-56.0	3.150	-57.4	3.129	-57.9	3.162	-42.0	3.357
1b	-47.2	3.267	-55.9	3.259	-51.1	3.318	-51.7	3.300	-51.2	3.349	-35.7	3.580
1c	-42.2	3.481	-50.5	3.507	-47.2	3.529	-46.7	3.516	-45.0	3.616	-29.9	3.889
2	-85.2	2.622	-104.1	2.594	-125.9	2.306	-99.4	2.632	-99.3	2.660	-82.8	2.814
3	-77.8	2.530	-89.5	2.514	-105.3	2.346	-85.6	2.552	-87.6	2.577	-67.6	2.752

Table S2. Differences in key geometrical factor and energy components, between the constrained optimization with the ring planished and fully optimized geometries and using M062X-D3 with cc-pVTZ (-PP) basis set.

	ΔR	$\Delta\Delta E_{def}$	$\Delta\Delta E_F$	$\Delta\Delta E_{pol}$	$\Delta\Delta E_{CT}$	$\Delta\Delta E_{disp}$	$\Delta\Delta E_{int}$	$\Delta\Delta E_b$
1a	0.029	-0.1	0.1	-0.1	0.2	0.0	0.2	0.1
1b	0.042	0.0	0.1	-0.1	0.2	0.0	0.2	0.1
1c	0.016	0.1	0.0	-0.1	0.2	0.0	0.1	0.2
2	0.010	-0.7	1.6	-0.1	0.2	0.0	1.6	0.9
3	-0.011	-0.8	1.9	-0.2	0.0	0.0	1.7	0.9

Table S3. NBO analysis of restricted optimized monomers.

C_6F_6		$C_6H_3(NO_2)_3$		$C_6H_3(CN)_3$	
LP(F)→π*	22.94	π(N=O)→π*	3.49	π(C≡N)→π*	8.49
π→LP*(F)	0.78	π→π*(N=O)	23.42	π→π*(C≡N)	17.18

Table S4. Frozen energies (in kJ/mol) evaluated with the Lewis structure using different methods with cc-pVTZ basis set at the optimal geometries of the electron localized state (BLW state).

Complex	M062x	B3LYP	CAMB3LYP	ωB97x	HF
1a	-56.3	-36.2	-43.6	-52.6	-33.4
1b	-50.3	-29.8	-36.8	-46.6	-27.1
1c	-43.8	-22.7	-29.6	-39.4	-19.9
2	-59.2	-35.0	-43.8	-55.1	-42.0
3	-52.6	-32.2	-39	-50.6	-29.5

Table S5. Energy decomposition results (in kJ/mol) of complexes in group with different binding sites.

	R	ΔE_{def}	ΔE_{F}	ΔE_{pol}	ΔE_{CT}	ΔE_{disp}	ΔE_{int}	ΔE_{b}
5a′	1.832	148.9	635.8	-454.3	-484.8	-0.6	-303.9	-154.9
5b′	2.025	131.9	493.9	-279.3	-459.4	-0.7	-245.4	-113.6
5c′	2.614	41.6	115.9	-64.3	-175.4	-0.8	-124.6	-83.0
6a′	2.826	2.7	-21.4	-47.8	-19.2	-0.9	-89.3	-86.6
6a″	2.640	6.5	11.0	-56.2	-45.9	-0.6	-91.7	-85.2
6a‴	2.910	1.7	-41.7	-45.7	-12.1	-0.7	-100.3	-98.6
6a⁗	2.828	2.0	-16.1	-49.3	-23.7	-0.7	-89.8	-87.7

Table S6. Cartesian coordinate (fully optimized geometries at M062x-D3/cc-pVTZ level of theory) and charges (NPA results of the Lewis structure) for the EPC model analysis.

Atom	x	y	z	Charge
Complex 1a				
C	0.00084	1.38010	-0.02083	0.316
C	-1.19334	0.68881	-0.02000	0.314
C	-1.19190	-0.69101	-0.02069	0.316
C	0.00381	-1.37969	-0.02226	0.316

C	1.19804	-0.68843	-0.02307	0.314
C	1.19652	0.69138	-0.02235	0.316
F	0.00530	-2.71041	-0.00164	-0.317
F	-2.34360	-1.35759	0.00168	-0.314
F	-2.34651	1.35275	0.00349	-0.315
F	-0.00057	2.71066	0.00189	-0.317
F	2.34832	1.35785	-0.00137	-0.314
F	2.35131	-1.35245	-0.00322	-0.315
CL	0.00177	0.00804	3.07839	-1.000
Complex 1b				
C	0.00167	1.38180	-0.03688	0.316
C	-1.19295	0.69011	-0.03379	0.314
C	-1.19148	-0.69019	-0.03253	0.316
C	0.00465	-1.37918	-0.03463	0.316
C	1.19938	-0.68767	-0.03780	0.314
C	1.19777	0.69269	-0.03881	0.316
F	0.00635	-2.70890	-0.01323	-0.317
F	-2.34225	-1.35631	-0.00863	-0.314
F	-2.34519	1.35386	-0.01154	-0.315
F	0.00035	2.71163	-0.01843	-0.317
F	2.34891	1.35885	-0.02236	-0.314
F	2.35192	-1.35124	-0.02022	-0.315
BR	-0.00914	-0.00546	3.25885	-1.000
Complex 1c				
C	0.00098	1.38167	-0.00828	0.316
C	-1.19401	0.69002	-0.00736	0.314
C	-1.19254	-0.69072	-0.00702	0.316

C	0.00398	-1.37971	-0.00754	0.316
C	1.19901	-0.68813	-0.00841	0.314
C	1.19749	0.69263	-0.00869	0.316
F	0.00544	-2.70891	0.01171	-0.317
F	-2.34293	-1.35655	0.01265	-0.314
F	-2.34585	1.35338	0.01172	-0.315
F	-0.00042	2.71093	0.00936	-0.317
F	2.34797	1.35851	0.00872	-0.314
F	2.35092	-1.35147	0.00963	-0.315
I	-0.00448	-0.00282	3.50660	-1.000
Complex 2				
C	0.98880	0.98843	-0.42080	-0.186
C	-0.35146	1.31169	-0.43375	0.0597
C	-1.35050	0.36158	-0.41959	-0.190
C	-0.96038	-0.96079	-0.43449	0.0594
C	0.36198	-1.35098	-0.42214	-0.189
C	1.31204	-0.35189	-0.43492	0.0566
H	1.75000	1.74970	-0.38257	0.292
H	0.64064	-2.39087	-0.38490	0.292
H	-2.39032	0.64025	-0.37952	0.293
N	-1.99997	-2.00046	-0.41884	0.555
O	-3.15459	-1.63733	-0.42657	-0.360
O	-1.63688	-3.15515	-0.41964	-0.356
N	-0.73191	2.73184	-0.41751	0.557
O	-1.91339	2.99485	-0.42383	-0.360
O	0.15979	3.55036	-0.41982	-0.360
N	2.73219	-0.73238	-0.41902	0.557

O	3.55069	0.15935	-0.42366	-0.360
O	2.99526	-1.91388	-0.42235	-0.358
CL	-0.00199	0.00569	2.59353	-1.000
Complex 3				
C	0.98608	0.98426	-0.55127	-0.117
C	1.33774	-0.35817	-0.55922	-0.143
C	0.36192	-1.34489	-0.55025	-0.118
C	-0.97639	-0.97831	-0.55588	-0.139
C	-1.34290	0.36011	-0.54634	-0.120
C	-0.35626	1.33591	-0.55644	-0.142
H	1.74910	1.74730	-0.52281	0.250
H	-2.38507	0.63935	-0.51315	0.251
H	0.64120	-2.38718	-0.52088	0.250
C	-0.72721	2.72083	-0.56035	0.268
N	-1.02357	3.82890	-0.60487	-0.258
C	2.72261	-0.72924	-0.56624	0.275
N	3.83050	-1.02594	-0.61233	-0.264
C	-1.99018	-1.99214	-0.55929	0.269
N	-2.80078	-2.80362	-0.60361	-0.262
CL	-0.02679	0.00285	2.51399	-1.000

3. The xyz coordinates of complexes and monomers optimized at M062x-D3/cc-pVTZ level of theory (in Å).

(1) $Cl^{-}\cdots C_6F_6$

C	6.0	0.0008449479	1.3800971456	-0.0208318656
C	6.0	-1.1933397359	0.6888057058	-0.0199963848
C	6.0	-1.1918975581	-0.6910053777	-0.0206880575
C	6.0	0.0038053836	-1.3796906460	-0.0222609596

C	6.0	1.1980368826	-0.6884343347	-0.0230734221
C	6.0	1.1965247809	0.6913789058	-0.0223492549
F	9.0	0.0053043834	-2.7104107191	-0.0016443933
F	9.0	-2.3435978686	-1.3575851854	0.0016772166
F	9.0	-2.3465050557	1.3527497866	0.0034856021
F	9.0	-0.0005718128	2.7106580887	0.0018888102
F	9.0	2.3483153130	1.3578506812	-0.0013725513
F	9.0	2.3513146821	-1.3524542362	-0.0032238839
CL	17.0	0.0017656574	0.0080391855	3.0783871442

(2) $Br^{-}\cdots C_6F_6$

C	6.0	0.0016664796	1.3817994534	-0.0368796124
C	6.0	-1.1929486481	0.6901125318	-0.0337922096
C	6.0	-1.1914777768	-0.6901944079	-0.0325265233
C	6.0	0.0046491673	-1.3791771172	-0.0346306984
C	6.0	1.1993770314	-0.6876652925	-0.0377988746
C	6.0	1.1977724136	0.6926928535	-0.0388128536
F	9.0	0.0063501310	-2.7088977049	-0.0132297197
F	9.0	-2.3422458168	-1.3563052702	-0.0086320852
F	9.0	-2.3451909349	1.3538583782	-0.0115358034
F	9.0	0.0003469624	2.7116302810	-0.0184309823
F	9.0	2.3489139363	1.3588523481	-0.0223637538
F	9.0	2.3519241537	-1.3512430398	-0.0202158293
BR	35.0	-0.0091370989	-0.0054640134	3.2588469456

(3) $I^{-}\cdots C_6F_6$

C	6.0	0.0009816613	1.3816726616	-0.0082817036
C	6.0	-1.1940064841	0.6900179129	-0.0073595700
C	6.0	-1.1925410424	-0.6907210478	-0.0070173131

C	6.0	0.0039795981	-1.3797080353	-0.0075389067
C	6.0	1.1990094462	-0.6881282609	-0.0084087334
C	6.0	1.1974852388	0.6926291410	-0.0086904576
F	9.0	0.0054429911	-2.7089131688	0.0117137362
F	9.0	-2.3429281990	-1.3565535464	0.0126479961
F	9.0	-2.3458457250	1.3533777047	0.0117157270
F	9.0	-0.0004226925	2.7109324386	0.0093583319
F	9.0	2.3479702495	1.3585050622	0.0087153317
F	9.0	2.3509243637	-1.3514720004	0.0096260299
I	53.0	-0.0044784057	-0.0028248616	3.5066015210

(4) $Cl^{-}\cdots C_6H_3(NO_2)_3$

C	6.0	0.9887973987	0.9884348836	-0.4208016023
C	6.0	-0.3514592698	1.3116890417	-0.4337481181
C	6.0	-1.3504965881	0.3615763775	-0.4195873007
C	6.0	-0.9603783048	-0.9607920944	-0.4344946344
C	6.0	0.3619847359	-1.3509786739	-0.4221434463
C	6.0	1.3120418263	-0.3518901466	-0.4349212683
H	1.0	1.7500049946	1.7496971173	-0.3825740045
H	1.0	0.6406366435	-2.3908727794	-0.3848969056
H	1.0	-2.3903226702	0.6402527545	-0.3795208594
N	7.0	-1.9999660279	-2.0004560197	-0.4188382488
O	8.0	-3.1545948990	-1.6373288464	-0.4265654376
O	8.0	-1.6368806142	-3.1551511984	-0.4196404754
N	7.0	-0.7319130207	2.7318378105	-0.4175144349
O	8.0	-1.9133933490	2.9948494027	-0.4238343504
O	8.0	0.1597874169	3.5503574568	-0.4198191476
N	7.0	2.7321948185	-0.7323840658	-0.4190215510

O	8.0	3.5506881132	0.1593493004	-0.4236586434
O	8.0	2.9952630526	-1.9138772188	-0.4223542254
CL	17.0	-0.0019942568	0.0056868983	2.5935346542

(5) $Br^{-}\cdots C_6H_3(NO_2)_3$

C	6.0	0.9880315065	0.9875423647	-0.4122761906
C	6.0	-0.3525154781	1.3107323226	-0.4261700246
C	6.0	-1.3518841145	0.3606815900	-0.4151460619
C	6.0	-0.9614049043	-0.9619227389	-0.4291592074
C	6.0	0.3611970045	-1.3524038056	-0.4166896806
C	6.0	1.3112447772	-0.3530564728	-0.4270228624
H	1.0	1.7492876779	1.7488651572	-0.3721281624
H	1.0	0.6399360157	-2.3924704148	-0.3802803543
H	1.0	-2.3918861666	0.6394486667	-0.3776189132
N	7.0	-2.0017457508	-2.0022560163	-0.4158609887
O	8.0	-3.1557280334	-1.6382940291	-0.4218186089
O	8.0	-1.6377645533	-3.1562682635	-0.4195659198
N	7.0	-0.7333370950	2.7318833240	-0.4079582226
O	8.0	-1.9147497558	2.9934808704	-0.4104421568
O	8.0	0.1589212852	3.5490622619	-0.4121441189
N	7.0	2.7324366823	-0.7338744386	-0.4098001013
O	8.0	3.5494999931	0.1584973390	-0.4155358997
O	8.0	2.9939388396	-1.9153298288	-0.4110120763
BR	35.0	0.0165220699	0.0256821119	2.7918295501

(6) $I^{-}\cdots C_6H_3(NO_2)_3$

C	6.0	0.9889104567	0.9892131351	-0.4164290808
C	6.0	-0.3519736760	1.3122702635	-0.4294303074
C	6.0	-1.3516398308	0.3620608926	-0.4181497757

C	6.0	-0.9610626830	-0.9607366432	-0.4303729270
C	6.0	0.3617481914	-1.3512956526	-0.4189833786
C	6.0	1.3119913652	-0.3516558841	-0.4296770380
H	1.0	1.7503189415	1.7506602284	-0.3790434936
H	1.0	0.6404606171	-2.3914559594	-0.3841918648
H	1.0	-2.3917836872	0.6407991696	-0.3823772149
N	7.0	-2.0018415716	-2.0015111478	-0.4198740423
O	8.0	-3.1551713784	-1.6363610596	-0.4269658496
O	8.0	-1.6367291619	-3.1548439433	-0.4245236811
N	7.0	-0.7328726157	2.7339415005	-0.4198885591
O	8.0	-1.9142056640	2.9946707277	-0.4257532419
O	8.0	0.1597963399	3.5504028337	-0.4273482291
N	7.0	2.7337099383	-0.7325674166	-0.4185105892
O	8.0	3.5499979950	0.1602606474	-0.4253878962
O	8.0	2.9942264108	-1.9139303256	-0.4228404285
I	53.0	0.0061200129	0.0000786340	3.0109475977

(7) $Cl^{-}\cdots C_6H_3(CN)_3$

C	6.0	0.9860788083	0.9842563647	-0.5512741485
C	6.0	1.3377408364	-0.3581695864	-0.5592237036
C	6.0	0.3619233277	-1.3448890062	-0.5502482584
C	6.0	-0.9763874364	-0.9783072106	-0.5558845518
C	6.0	-1.3429045729	0.3601108460	-0.5463384934
C	6.0	-0.3562643234	1.3359081655	-0.5564390241
H	1.0	1.7490974947	1.7472960941	-0.5228111606
H	1.0	-2.3850719210	0.6393471982	-0.5131506571
H	1.0	0.6412001774	-2.3871803147	-0.5208818663
C	6.0	-0.7272092526	2.7208277805	-0.5603506103

N	7.0	-1.0235668466	3.8288965272	-0.6048678477
C	6.0	2.7226117794	-0.7292384661	-0.5662419611
N	7.0	3.8304983924	-1.0259431278	-0.6123291551
C	6.0	-1.9901763296	-1.9921434804	-0.5592900747
N	7.0	-2.8007825485	-2.8036198432	-0.6036107889
CL	17.0	-0.0267875858	0.0028480588	2.5139949416

(8) $Br^{-}\cdots C_6H_3(CN)_3$

C	6.0	0.9848851132	0.9846255503	-0.5600036723
C	6.0	1.3366052842	-0.3581147071	-0.5673955199
C	6.0	0.3606534166	-1.3451428372	-0.5602655507
C	6.0	-0.9780462361	-0.9783941396	-0.5667265029
C	6.0	-1.3448974420	0.3603219068	-0.5585453691
C	6.0	-0.3578960859	1.3362450662	-0.5667710236
H	1.0	1.7478808968	1.7476669765	-0.5317636255
H	1.0	-2.3871927188	0.6395609313	-0.5285109322
H	1.0	0.6399579302	-2.3874385842	-0.5322425906
C	6.0	-0.7289907587	2.7213652476	-0.5761327605
N	7.0	-1.0255502500	3.8289287235	-0.6263449742
C	6.0	2.7217151244	-0.7292300849	-0.5764423942
N	7.0	3.8292524582	-1.0259061415	-0.6258450605
C	6.0	-1.9918975718	-1.9924782122	-0.5759373382
N	7.0	-2.8030243780	-2.8028543719	-0.6259670137
BR	35.0	-0.0034547823	0.0008446763	2.7099469679

(9) $I^{-}\cdots C_6H_3(CN)_3$

C	6.0	0.9849791446	0.9850336121	-0.5733550657
C	6.0	1.3366748285	-0.3580117672	-0.5800030754
C	6.0	0.3605548637	-1.3452401686	-0.5732282198

C	6.0	-0.9784086179	-0.9783379650	-0.5797555038
C	6.0	-1.3453419531	0.3606246032	-0.5729270913
C	6.0	-0.3580690551	1.3367060440	-0.5799747710
H	1.0	1.7480121029	1.7480461335	-0.5453667740
H	1.0	-2.3876355154	0.6399226079	-0.5444887155
H	1.0	0.6398752709	-2.3875330646	-0.5451680797
C	6.0	-0.7292157928	2.7219180748	-0.5912859855
N	7.0	-1.0258091291	3.8292046690	-0.6435571424
C	6.0	2.7218839125	-0.7291407722	-0.5909348458
N	7.0	3.8291993406	-1.0258269878	-0.6427535001
C	6.0	-1.9924448627	-1.9923655384	-0.5905877187
N	7.0	-2.8030123512	-2.8030447616	-0.6422063791
I	53.0	-0.0012421864	-0.0019547191	2.9266455077

(10) $Cl^{-}\cdots$1,2-quinone

C	6.0	-0.8483242866	-1.1210775780	0.1445188291
C	6.0	0.6899603048	-1.1176640229	0.1348536112
C	6.0	1.3433270609	0.2102524386	0.0615906503
C	6.0	0.6406887333	1.3283926712	-0.1455694199
C	6.0	-0.8138536378	1.3251292283	-0.1358387685
C	6.0	-1.5085121112	0.2037186859	0.0801796174
H	1.0	-2.5890586678	0.1911236136	0.1364243349
H	1.0	-1.3290752140	2.2714332969	-0.2468493360
H	1.0	1.1501390811	2.2769860708	-0.2640563127
H	1.0	2.4245442356	0.2026187676	0.1036237640
O	8.0	-1.4749351283	-2.1475080594	0.0224690885
O	8.0	1.3195562737	-2.1414027665	0.0051956288
CL	17.0	-0.0634947438	-0.5655084559	2.7460139723

(11) $Br^{-}\cdots$1,2-quinone

C	6.0	-0.8563621492	-1.1280891492	0.1507546094
C	6.0	0.6850523958	-1.1262792576	0.1513213430
C	6.0	1.3407638906	0.1987612886	0.0847381407
C	6.0	0.6381605950	1.3164015764	-0.1302718137
C	6.0	-0.8155788746	1.3150035950	-0.1293656883
C	6.0	-1.5151696617	0.1956478070	0.0863018263
H	1.0	-2.5956459201	0.1859416018	0.1398073272
H	1.0	-1.3281966279	2.2623177319	-0.2418856562
H	1.0	1.1481402604	2.2649386478	-0.2443295882
H	1.0	2.4213220773	0.1918088741	0.1366825633
O	8.0	-1.4779881514	-2.1569830854	0.0307246246
O	8.0	1.3093342445	-2.1539742525	0.0344601149
BR	35.0	-0.0928701787	-0.4990014880	2.9826272528

(12) $I^{-}\cdots$1,2-quinone

C	6.0	-0.8591317282	-1.1482441761	0.1736653268
C	6.0	0.6840658095	-1.1465162653	0.1626495083
C	6.0	1.3389868380	0.1751059004	0.0562005159
C	6.0	0.6335254173	1.2849390277	-0.1944554991
C	6.0	-0.8184685443	1.2829653919	-0.1858725009
C	6.0	-1.5182829163	0.1714889675	0.0733768114
H	1.0	-2.5983084136	0.1647369777	0.1329702415
H	1.0	-1.3314976129	2.2261694773	-0.3260103449
H	1.0	1.1427441990	2.2294629281	-0.3396916569
H	1.0	2.4195903028	0.1707516524	0.1038523191
O	8.0	-1.4787359747	-2.1826672630	0.0986664543
O	8.0	1.3047078563	-2.1793970683	0.0754243313

I	53.0	-0.0582333329	-0.3523016604	3.2199783673

(13) $Cl^{-}\cdots$1,3-quinone

C	6.0	-0.7150038959	-1.0147261287	0.5456735341
C	6.0	0.8138309468	-1.0164367135	0.3218530269
C	6.0	1.3973356693	0.1917278597	-0.0905951736
C	6.0	0.6844852605	1.3919626923	-0.3135625141
C	6.0	-0.7965108709	1.3544569334	-0.0819787381
C	6.0	-1.4398836250	0.2655818409	0.3152189613
H	1.0	-2.5099439402	0.2635987955	0.4911134702
H	1.0	-1.3125971340	2.2905954198	-0.2597410488
O	8.0	1.1701034469	2.4698615283	-0.6876981470
H	1.0	2.4652612539	0.1979407849	-0.2636186969
H	1.0	-1.1255347548	-1.8149843511	-0.0687808961
O	8.0	1.3860458867	-2.0993177191	0.4889746718
CL	17.0	-1.0766263433	-1.5637670520	2.2456972095

(14) $Br^{-}\cdots$1,3-quinone

C	6.0	-0.7241238340	-1.0009779598	0.5818228511
C	6.0	0.7959069163	-1.0317767862	0.3243271879
C	6.0	1.3925650927	0.1741221310	-0.0743616618
C	6.0	0.6901955164	1.3818019693	-0.2938780990
C	6.0	-0.7936014301	1.3522806473	-0.0825169387
C	6.0	-1.4467815190	0.2698070078	0.3193685853
H	1.0	-2.5196150855	0.2734126190	0.4752394694
H	1.0	-1.3050243846	2.2864294596	-0.2824588562
O	8.0	1.1875883908	2.4576640541	-0.6575965659
H	1.0	2.4592360416	0.1693399467	-0.2544476492
H	1.0	-1.1675890118	-1.8414184660	0.0530581011

O	8.0	1.3513775194	-2.1280027057	0.4614907100
BR	35.0	-1.0591723121	-1.4961880271	2.4815179219

(15) I^{-}···1,3-quinone

C	6.0	-0.7129089410	-0.9918209924	0.6178355768
C	6.0	0.7981584962	-1.0395395102	0.3355848975
C	6.0	1.3893857491	0.1537764160	-0.1066677253
C	6.0	0.6818992461	1.3518316329	-0.3645656066
C	6.0	-0.8005854961	1.3236807081	-0.1516987352
C	6.0	-1.4469871910	0.2537944476	0.2971546426
H	1.0	-2.5208748900	0.2565556202	0.4449160415
H	1.0	-1.3190821462	2.2428307253	-0.3968620290
O	8.0	1.1760569641	2.4148876635	-0.7679503710
H	1.0	2.4536961809	0.1438362963	-0.2997273619
H	1.0	-1.1731135136	-1.8766925644	0.1865960020
O	8.0	1.3552067938	-2.1328008585	0.4968604229
I	53.0	-1.0198893524	-1.4038456946	2.7592781196

(16) Cl^{-}···1,4-quinone

C	6.0	-0.0200465418	1.4654239085	-0.0566957512
C	6.0	-1.2889024731	0.7442669011	-0.2722163028
C	6.0	-1.2759547692	-0.7152012103	-0.0559607071
C	6.0	-0.1455830665	-1.3645884512	0.2238256208
C	6.0	1.1659779226	-0.6696022869	0.1626915984
C	6.0	1.1090062951	0.8137038757	0.2228984612
H	1.0	-0.1021994339	-2.4298755649	0.4032847796
H	1.0	-2.2388981274	-1.2071022794	-0.0978242924
O	8.0	-2.3116490486	1.3330584733	-0.5851205960
H	1.0	-0.0777042468	2.5451893186	-0.0991334440

H	1.0	2.0523760954	1.3107361965	0.4016389571
O	8.0	2.1804000819	-1.2539904523	-0.1584782667
CL	17.0	0.9831773121	-0.5620194285	2.8610879433

(17) Br^-···1,4-quinone

C	6.0	-0.0130634685	1.4639199200	-0.0802958054
C	6.0	-1.2826361798	0.7434526241	-0.2982683011
C	6.0	-1.2734217337	-0.7152344397	-0.0751283818
C	6.0	-0.1450304034	-1.3665880476	0.2076375160
C	6.0	1.1662310735	-0.6717494298	0.1445395058
C	6.0	1.1150259299	0.8117985769	0.2017017707
H	1.0	-0.1038899962	-2.4314921590	0.3896530496
H	1.0	-2.2372174683	-1.2052570229	-0.1167653459
O	8.0	-2.3013927053	1.3322344227	-0.6207658696
H	1.0	-0.0692343834	2.5435029689	-0.1263824976
H	1.0	2.0591045267	1.3080614082	0.3788567922
O	8.0	2.1860417237	-1.2621532876	-0.1416737831
BR	35.0	0.9294830847	-0.5404965341	3.0868893501

(18) I^-···1,4-quinone

C	6.0	-0.0152314509	1.4628719042	-0.0983094057
C	6.0	-1.2825831116	0.7403401663	-0.3203141265
C	6.0	-1.2709450239	-0.7183466758	-0.0968919516
C	6.0	-0.1416473692	-1.3689819617	0.1853755924
C	6.0	1.1677170505	-0.6702422931	0.1258579543
C	6.0	1.1144672861	0.8129883919	0.1841896672
H	1.0	-0.0990766970	-2.4347815542	0.3621603266
H	1.0	-2.2334840961	-1.2103951252	-0.1413881118
O	8.0	-2.3009663790	1.3265149904	-0.6475982541

H	1.0	-0.0728716289	2.5423355081	-0.1441129645
H	1.0	2.0576213299	1.3114872816	0.3598042782
O	8.0	2.1927564481	-1.2604315834	-0.1403388959
I	53.0	0.9142436421	-0.5233600492	3.3215638914

(19) C_6F_6

C	6.0	0.0003955695	1.3853113786	-0.0124602972
C	6.0	-1.1977572603	0.6916989010	-0.0115586899
C	6.0	-1.1962936376	-0.6928697133	-0.0117903278
C	6.0	0.0033745131	-1.3838264209	-0.0122601534
C	6.0	1.2017100082	-0.6901762640	-0.0123624816
C	6.0	1.2001752794	0.6942121982	-0.0131709959
F	9.0	0.0048906918	-2.7082209959	-0.0126155141
F	9.0	-2.3426675251	-1.3562158945	-0.0114759222
F	9.0	-2.3456524064	1.3524675846	-0.0106164588
F	9.0	-0.0009642481	2.7096869727	-0.0125578794
F	9.0	2.3465386852	1.3576644771	-0.0146364733
F	9.0	2.3494566313	-1.3511824375	-0.0117592757

(20) $C_6H_3(NO_2)_3$

C	6.0	0.9909951450	0.9909917210	-0.4216000000
C	6.0	-0.3519880071	1.3136208272	-0.4216000000
C	6.0	-1.3537357388	0.3627233971	-0.4216000000
C	6.0	-0.9616566318	-0.9616541718	-0.4216000000
C	6.0	0.3627228082	-1.3537368832	-0.4216000000
C	6.0	1.3136164901	-0.3519947520	-0.4216000000
H	1.0	1.7540478800	1.7540418163	-0.4216000000
H	1.0	0.6420351328	-2.3960768439	-0.4216000000
H	1.0	-2.3960766544	0.6420283928	-0.4216000000

N	7.0	-2.0089375121	-2.0089342630	-0.4216000000
O	8.0	-3.1561278844	-1.6346027095	-0.4216000000
O	8.0	-1.6346078477	-3.1561262763	-0.4216000000
N	7.0	-0.7352980351	2.7442167205	-0.4216000000
O	8.0	-1.9159541985	2.9937019216	-0.4216000000
O	8.0	0.1625106766	3.5505360050	-0.4216000000
N	7.0	2.7442143404	-0.7352985337	-0.4216000000
O	8.0	3.5505296860	0.1625141474	-0.4216000000
O	8.0	2.9937103507	-1.9159505153	-0.4216000000

(21) $C_6H_3(CN)_3$

C	6.0	0.9867187980	0.9867146684	-0.5636842100
C	6.0	1.3390704877	-0.3587895713	-0.5636842100
C	6.0	0.3611586555	-1.3478406398	-0.5636842100
C	6.0	-0.9802614815	-0.9802617909	-0.5636842100
C	6.0	-1.3478380319	0.3611624018	-0.5636842100
C	6.0	-0.3587875653	1.3390712332	-0.5636842100
H	1.0	1.7505502131	1.7505381817	-0.5636842100
H	1.0	-2.3912357921	0.6407549632	-0.5636842100
H	1.0	0.6407349622	-2.3912419826	-0.5636842100
C	6.0	-0.7301906098	2.7250588720	-0.5636842100
N	7.0	-1.0270434930	3.8326542586	-0.5636842100
C	6.0	2.7250501737	-0.7302229436	-0.5636842100
N	7.0	3.8326707514	-1.0270041653	-0.5636842100
C	6.0	-1.9948923583	-1.9948652162	-0.5636842100
N	7.0	-2.8057047097	-2.8057282692	-0.5636842100

(22) 1,2-quinone

C	6.0	-0.8650033837	-1.1423137239	0.1197766957

C	6.0	0.6892962535	-1.1405150074	0.1101691827
C	6.0	1.3597305457	0.1652274275	-0.0311592828
C	6.0	0.6407914680	1.2852806963	-0.1444392216
C	6.0	-0.8252122313	1.2835780542	-0.1352371794
C	6.0	-1.5401777243	0.1619127915	-0.0130562333
H	1.0	-2.6213699655	0.1538586800	-0.0056506310
H	1.0	-1.3261731345	2.2379359941	-0.2324048071
H	1.0	1.1382338300	2.2408193412	-0.2478442488
H	1.0	2.4409618847	0.1597525111	-0.0372552589
O	8.0	-1.4606861739	-2.1799868051	0.2323391737
O	8.0	1.2888038642	-2.1767544092	0.2155373175

(23) 1,3-quinone

C	6.0	-0.6903077950	-0.9378672471	0.3759367740
C	6.0	0.7851328023	-1.0396247218	0.2230674114
C	6.0	1.4307278450	0.1514927573	-0.1444914147
C	6.0	0.7041962871	1.3424780578	-0.3454833432
C	6.0	-0.7987905898	1.3318285768	-0.1606526944
C	6.0	-1.4722809069	0.2064506495	0.1940858509
H	1.0	-2.5439954439	0.1825572066	0.3297668862
H	1.0	-1.2835940303	2.2850092184	-0.3334482442
O	8.0	1.1456700185	2.4454310600	-0.6666031853
H	1.0	2.5012033004	0.1707391742	-0.2784354716
H	1.0	-1.1561354836	-1.8774709442	0.6588334127
O	8.0	1.2590252485	-2.1606842031	0.4388997725

(24) 1,4-quinone

C	6.0	0.0592666815	1.4326975731	-0.0123617490
C	6.0	-1.2415872991	0.7169897130	-0.0117673955

C	6.0	-1.2078493283	-0.7674667936	-0.0116188576
C	6.0	-0.0553707155	-1.4312806413	-0.0121008985
C	6.0	1.2454882682	-0.7155297356	-0.0129006226
C	6.0	1.2117422666	0.7688917715	-0.0129699325
H	1.0	0.0028632527	-2.5116603537	-0.0119348554
H	1.0	-2.1715430344	-1.2593175018	-0.0110545338
O	8.0	-2.2893280094	1.3202035870	-0.0113343298
H	1.0	0.0009354347	2.5130764570	-0.0122848622
H	1.0	2.1754045578	1.2607979765	-0.0134403725
O	8.0	2.2931842260	-1.3188522661	-0.0134960598

4. The xyz coordinates of complexes and monomers optimized at M062x-D3/cc-pVDZ level of theory (in Å).

(1) Cl^- is inside the fluorinated [5] cycloparaphenylene

C	6.0	3.4950097644	0.0086566748	1.1464013441
C	6.0	0.7362451717	2.6980795513	0.9704565168
C	6.0	3.3100570916	1.3814235237	1.0357304611
C	6.0	2.8331932664	2.1310064906	2.1261688717
C	6.0	-0.6369449534	2.8461107817	1.1259255139
C	6.0	-1.2025718495	3.2697443865	2.3244004192
C	6.0	1.6473118552	3.0258936668	1.9786754589
C	6.0	-3.4147008901	2.1927060533	1.7526514480
C	6.0	-2.4357624142	2.5195433023	2.7023501355
C	6.0	-2.3645933661	-1.5425107683	0.9292898275
C	6.0	-3.9254825264	0.9061296417	1.6572070812
C	6.0	-3.5100814684	-0.1084443447	2.5273023572
C	6.0	-1.3197878587	-2.4548988972	0.8368736825
C	6.0	-1.0499190149	-3.3568702756	1.8689634510

C	6.0	-3.1858254231	-1.4877765830	2.0601743556
C	6.0	1.4512505511	-3.5806350503	1.3152275699
C	6.0	0.3926176505	-3.4749816308	2.2317561403
C	6.0	2.6473953290	-2.8982689022	1.4889625707
C	6.0	2.8338791678	-1.9785377726	2.5464072093
C	6.0	3.4915370893	-0.6476484031	2.3881370436
C	6.0	3.5944944490	0.2389629248	3.4751517512
C	6.0	1.0797643099	3.7668410349	3.0406248751
C	6.0	3.1441178195	1.5490237752	3.3660228495
C	6.0	-0.2933630615	3.8410716167	3.2293226097
C	6.0	-2.2632459926	1.5949217323	3.7351211670
C	6.0	-3.0941807155	-2.5789443532	2.9318696616
C	6.0	-2.7895991866	0.3109599150	3.6504450955
C	6.0	-2.0621176847	-3.5005867601	2.8290547905
C	6.0	0.7216885254	-2.8404315997	3.4254033969
C	6.0	1.8839785277	-2.0914025569	3.5657781202
F	9.0	1.8783140698	-1.2942634521	4.6241911035
F	9.0	-0.1858456803	-2.6599779310	4.3794596138
F	9.0	1.2842283066	-4.2826980302	0.1884358512
F	9.0	3.5931642169	-3.0665520701	0.5583141524
F	9.0	3.5355386546	-0.6699121766	0.0000201856
F	9.0	3.4212061001	1.9208578849	-0.1803534479
F	9.0	1.0765912096	1.9793023782	-0.0894413628
F	9.0	-1.3770389552	2.2731254510	0.1822821753
F	9.0	-3.7233321212	3.0685064567	0.7894585455
F	9.0	-4.6882350828	0.6033539586	0.6008166591
F	9.0	-2.3818980960	-0.5596203971	0.0353724491

F	9.0	-0.4315256838	-2.2609643762	-0.1311846605
F	9.0	3.9487563347	-0.1906315764	4.6884516604
F	9.0	2.8896418139	2.1770844830	4.5136309597
F	9.0	1.8597411558	4.3441287333	3.9612004318
F	9.0	-0.7447583025	4.3974183642	4.3594495473
F	9.0	-1.3728764060	1.8215646237	4.6939989329
F	9.0	-2.3558780415	-0.5735571152	4.5421050633
F	9.0	-3.9020240858	-2.6475792273	3.9958804534
F	9.0	-1.9371925197	-4.4195103349	3.7933850413
CL	17.0	0.0653630200	0.0123837195	2.2789181707

(2) Br^- is inside the fluorinated [5] cycloparaphenylene

C	6.0	3.4950504993	0.0145096768	1.1455039722
C	6.0	0.7523148271	2.7792688905	0.9658146449
C	6.0	3.3077198397	1.3886796036	1.0404952856
C	6.0	2.8315138249	2.1379444542	2.1328997594
C	6.0	-0.6255473986	2.9202224639	1.1136645489
C	6.0	-1.2069773285	3.2957027688	2.3203389571
C	6.0	1.6561934481	3.0539034354	1.9957343178
C	6.0	-3.4004839410	2.1894797058	1.7010182672
C	6.0	-2.4448423725	2.5371851985	2.6712343010
C	6.0	-2.3951023128	-1.5925242718	0.9316524481
C	6.0	-3.9078136467	0.9016125165	1.6126604818
C	6.0	-3.5187608945	-0.1037350365	2.5099037407
C	6.0	-1.3486749136	-2.5080250141	0.8474426036
C	6.0	-1.0491569982	-3.3745223934	1.9008125374
C	6.0	-3.1916850332	-1.4945144271	2.0761572826
C	6.0	1.4446751008	-3.5823069645	1.2951601250

C	6.0	0.4010225311	-3.4998831109	2.2352769160
C	6.0	2.6418266847	-2.8982183515	1.4549147163
C	6.0	2.8551707062	-1.9999006261	2.5288473331
C	6.0	3.4934257424	-0.6545606472	2.3816069384
C	6.0	3.5956456300	0.2313300995	3.4707242884
C	6.0	1.0745788923	3.7645890215	3.0736185134
C	6.0	3.1461334081	1.5435804091	3.3670619875
C	6.0	-0.3004689979	3.8407833766	3.2480871175
C	6.0	-2.3136097228	1.6302856100	3.7247540974
C	6.0	-3.0800520548	-2.5646529193	2.9756236269
C	6.0	-2.8384316146	0.3420868700	3.6467668275
C	6.0	-2.0499389286	-3.4888329243	2.8810199162
C	6.0	0.7677498236	-2.9040936838	3.4376265222
C	6.0	1.9371534936	-2.1588692929	3.5704014050
F	9.0	1.9534467053	-1.3935636572	4.6525600882
F	9.0	-0.1125142866	-2.7426901685	4.4202505831
F	9.0	1.2648838740	-4.2738769826	0.1641602607
F	9.0	3.5658489445	-3.0524273094	0.5005330402
F	9.0	3.5432205249	-0.6534002537	-0.0069024383
F	9.0	3.4324724081	1.9285107051	-0.1739558668
F	9.0	1.0974521313	2.0959389708	-0.1164252736
F	9.0	-1.3503572678	2.3719058414	0.1435249042
F	9.0	-3.7077595096	3.0567692684	0.7298358297
F	9.0	-4.6618453899	0.5904183964	0.5525264181
F	9.0	-2.4237138174	-0.6410664779	0.0042768578
F	9.0	-0.4837614025	-2.3350334253	-0.1459427453
F	9.0	3.9624539423	-0.1921397236	4.6823735638

F	9.0	2.9021049887	2.1650144155	4.5205229363
F	9.0	1.8418419454	4.3192081773	4.0180874796
F	9.0	-0.7582475144	4.3831779739	4.3821817339
F	9.0	-1.4530714528	1.8653654928	4.7088397555
F	9.0	-2.4247926802	-0.5211087523	4.5687531984
F	9.0	-3.8837218044	-2.6208103519	4.0434282579
F	9.0	-1.9285776357	-4.3997472485	3.8533090815
BR	35.0	0.0623130731	0.0146272119	2.2874861561

(3) I^- is inside the fluorinated [5] cycloparaphenylene

C	6.0	3.5850848431	-0.0146434063	1.1488112235
C	6.0	0.7016004919	2.5720246175	0.9690388504
C	6.0	3.3205486077	1.3421711766	1.0454605296
C	6.0	2.8121471960	2.0738181083	2.1354325588
C	6.0	-0.6697082264	2.7610756445	1.0717019351
C	6.0	-1.2771874936	3.2710506699	2.2212618197
C	6.0	1.6007974715	2.9369903143	1.9768185736
C	6.0	-3.4768690274	2.2305917496	1.5409649375
C	6.0	-2.5917540455	2.6371937517	2.5591730195
C	6.0	-2.3130533269	-1.4392413274	1.0140862448
C	6.0	-3.9750560874	0.9391644623	1.4968986184
C	6.0	-3.6445763036	-0.0065391741	2.4845042915
C	6.0	-1.2534112844	-2.3352144721	0.9378769102
C	6.0	-0.9653733595	-3.2423103571	1.9606241723
C	6.0	-3.1640828752	-1.3765520842	2.1233326035
C	6.0	1.4686139863	-3.5207093436	1.1976154099
C	6.0	0.4844188732	-3.4971549822	2.2115143789
C	6.0	2.6902364915	-2.8882093947	1.3369693379

C	6.0	3.0040559167	-2.0938681690	2.4801422598
C	6.0	3.5851922830	-0.7075215098	2.3829116380
C	6.0	3.6322180254	0.1851935345	3.4729643516
C	6.0	1.0102156605	3.7136467884	2.9953332261
C	6.0	3.1425654756	1.4915884289	3.3633736362
C	6.0	-0.3715787112	3.8472810762	3.1289830113
C	6.0	-2.6243335201	1.8390731717	3.7065831238
C	6.0	-3.0247807067	-2.4455902519	3.0217167759
C	6.0	-3.1454082314	0.5410452855	3.6707525343
C	6.0	-1.9735432472	-3.3550846870	2.9351160938
C	6.0	0.9854602378	-3.1118781710	3.4471053248
C	6.0	2.2039291091	-2.4274709936	3.5777338758
F	9.0	2.3858405717	-1.9027061453	4.7791168411
F	9.0	0.2118440018	-3.0982993547	4.5238491046
F	9.0	1.2035609260	-4.1185042497	0.0298572796
F	9.0	3.5521383512	-3.0146959296	0.3222055037
F	9.0	3.7346150339	-0.6534516236	-0.0127681039
F	9.0	3.4107331261	1.8929558400	-0.1683006338
F	9.0	1.0529564303	1.7813166282	-0.0401677737
F	9.0	-1.3818832145	2.1581160472	0.1212966913
F	9.0	-3.6901749316	3.0261937921	0.4868486942
F	9.0	-4.6285610032	0.5517337191	0.3959322571
F	9.0	-2.3196172136	-0.4803594596	0.0901975383
F	9.0	-0.3796732758	-2.1159979264	-0.0420263196
F	9.0	3.9750901715	-0.2005185952	4.7007179950
F	9.0	2.8448834862	2.0931030090	4.5108243902
F	9.0	1.7681864360	4.2828822161	3.9363247179

F	9.0	-0.8281701008	4.4903881432	4.2077207412
F	9.0	-1.9172604017	2.1764756034	4.7764712929
F	9.0	-2.8946606750	-0.2365826764	4.7153376529
F	9.0	-3.8655796520	-2.5624299974	4.0537037998
F	9.0	-1.8961589891	-4.3007677320	3.8760350083
I	53.0	0.0718141052	0.0228247756	3.1682693566

(4) Cl^- is outside the fluorinated [5] cycloparaphenylene

C	6.0	2.2455269082	-0.2348833215	1.1862059922
C	6.0	0.8792489544	3.7430149604	1.0280057766
C	6.0	2.1267621040	1.1422104225	1.0361534182
C	6.0	2.4067582200	2.0204113839	2.0852946905
C	6.0	-0.4251697929	3.3995172658	1.1425287319
C	6.0	-1.0400107281	3.0627436883	2.3967424575
C	6.0	1.8535183920	3.4705263670	2.1265327005
C	6.0	-3.0421562404	1.7102885388	1.5941781135
C	6.0	-1.9946452094	1.9903352965	2.5153247087
C	6.0	-2.5157264919	-2.2663014749	0.9211043332
C	6.0	-3.6664141158	0.4760526321	1.5227383760
C	6.0	-3.4105440058	-0.5550667139	2.4373709388
C	6.0	-1.5618438133	-3.2698085960	0.9305285207
C	6.0	-1.3434776934	-4.0722400421	2.0685581703
C	6.0	-3.2749427656	-1.9855772959	2.0660867017
C	6.0	1.1574872749	-4.1318437366	1.6145264041
C	6.0	0.0888065496	-4.0698317143	2.5178556633
C	6.0	2.2019209916	-3.2095644960	1.6699803004
C	6.0	2.2396426005	-2.2047343810	2.6417191501
C	6.0	2.7068320332	-0.8220259401	2.3658633199

C	6.0	3.1594577953	0.0638415289	3.3489124923
C	6.0	1.0975432867	3.7363117396	3.4051884395
C	6.0	3.0041413401	1.4370228282	3.2103381667
C	6.0	-0.2187473935	3.4576087416	3.5092356505
C	6.0	-1.8797116578	1.0145772147	3.5340228025
C	6.0	-3.3239154915	-3.0183822927	3.0103332207
C	6.0	-2.5610046256	-0.1821636212	3.4987538309
C	6.0	-2.3877252194	-4.0515199675	3.0032222608
C	6.0	0.3304171525	-3.2958919562	3.6685698325
C	6.0	1.3599816048	-2.3706299601	3.7159713347
F	9.0	1.3301788233	-1.4868587895	4.7090182082
F	9.0	-0.5729649161	-3.2162224982	4.6424765164
F	9.0	1.0962687740	-4.9440330548	0.5582479425
F	9.0	3.0755920876	-3.1967122559	0.6634994341
F	9.0	1.7651633222	-0.9986865163	0.2005822845
F	9.0	1.6392713123	1.5561207929	-0.1316784200
F	9.0	1.3942578149	4.0779855700	-0.1737675471
F	9.0	-1.1294904105	3.2993000100	0.0036628707
F	9.0	-3.4263114872	2.6381111843	0.7085729590
F	9.0	-4.5045547766	0.2436746156	0.5029331121
F	9.0	-2.4590482319	-1.3891421602	-0.0793140306
F	9.0	-0.6577135300	-3.2274749674	-0.0505576099
F	9.0	3.6753113038	-0.4132187726	4.4848364070
F	9.0	3.3741836305	2.2066560532	4.2326494618
F	9.0	1.7793482944	4.2428251572	4.4491419911
F	9.0	-0.8035464631	3.6053632479	4.7136114836
F	9.0	-0.9383022926	1.1224192053	4.4817460017

F	9.0	-2.1935100993	-1.1019841068	4.4036816903
F	9.0	-4.1104443178	-2.9059140755	4.0817041933
F	9.0	-2.3563519505	-4.8728998735	4.0543510064
CL	17.0	3.2774224191	4.6182153484	2.0056925198

(5) Br^- is outside the fluorinated [5] cycloparaphenylene

C	6.0	3.3433406472	-0.2241482074	1.2077259431
C	6.0	0.5791789700	2.4864644716	1.0396090867
C	6.0	3.1627247727	1.1501126852	1.0795833715
C	6.0	2.6799186952	1.9017050610	2.1758241800
C	6.0	-0.7885394032	2.5638600237	1.2132238620
C	6.0	-1.3811999259	2.9563143004	2.4193824795
C	6.0	1.5094580729	2.8197362532	2.0486645362
C	6.0	-3.5851634412	1.8707502998	1.8297427011
C	6.0	-2.5828205515	2.1562804317	2.7672098669
C	6.0	-2.6086700374	-1.8659764407	0.9669366806
C	6.0	-4.1364473480	0.5945501362	1.7235059887
C	6.0	-3.7580865660	-0.4368812042	2.5852935686
C	6.0	-1.5590511066	-2.7664065274	0.8790475878
C	6.0	-1.2745734267	-3.6581120098	1.9239506444
C	6.0	-3.4335507155	-1.8142859905	2.1044527546
C	6.0	1.2467073816	-3.8455924951	1.4243999851
C	6.0	0.1699799876	-3.7185776764	2.3072852373
C	6.0	2.4368967493	-3.1346499793	1.5973088156
C	6.0	2.5815388001	-2.1560534057	2.6017250510
C	6.0	3.3352894470	-0.8798241704	2.4508789245
C	6.0	3.4365212496	0.0113903088	3.5366629642
C	6.0	0.9012453010	3.4897133461	3.1496620348

C	6.0	3.0296027376	1.3330219820	3.4188193873
C	6.0	-0.4719511402	3.5088505055	3.3398567732
C	6.0	-2.4553836337	1.2240906505	3.8072466104
C	6.0	-3.3476895382	-2.9141280699	2.9603827903
C	6.0	-3.0234315037	-0.0360977748	3.7159729354
C	6.0	-2.2911677411	-3.8222379267	2.8682384937
C	6.0	0.4528838914	-2.9922539525	3.4696241382
C	6.0	1.5904387190	-2.2156261139	3.5976248728
F	9.0	1.5532501606	-1.3634686762	4.6191667555
F	9.0	-0.4901096401	-2.7920847080	4.3917856765
F	9.0	1.1095703504	-4.5736624644	0.3152929167
F	9.0	3.3956285344	-3.3293517291	0.6937143442
F	9.0	3.3520400070	-0.9254749367	0.0710064820
F	9.0	3.2837370662	1.6531194036	-0.1366796215
F	9.0	0.9500081007	1.8633976549	-0.0805445866
F	9.0	-1.5253299962	1.9823257353	0.2574056400
F	9.0	-3.8776880756	2.7521297247	0.8747254021
F	9.0	-4.8882618015	0.3209000339	0.6561163531
F	9.0	-2.6323613959	-0.8798279590	0.0729280801
F	9.0	-0.6604317022	-2.5661969384	-0.0834672233
F	9.0	3.7329918491	-0.4408862903	4.7570831553
F	9.0	2.8297975827	1.9820732204	4.5647949927
F	9.0	1.6486805185	4.0699516303	4.0810124203
F	9.0	-0.9383507408	4.0003833649	4.4946133036
F	9.0	-1.5666638604	1.4207833788	4.7795904069
F	9.0	-2.6197387521	-0.9446539355	4.6074042969
F	9.0	-4.1435085536	-2.9922130680	4.0261998305

F	9.0	-2.1402752222	-4.7009978612	3.8588717317
BR	35.0	3.3357854978	4.7192883390	1.2020745979

(6) I^- is outside the fluorinated [5] cycloparaphenylene

C	6.0	3.4036444831	-0.1007526095	1.1434713949
C	6.0	0.6343226248	2.2687771268	1.0635470533
C	6.0	3.3152514974	1.2664038424	1.0348357986
C	6.0	2.9117794690	2.0984045300	2.1389399369
C	6.0	-0.7430416081	2.3136293855	1.2150266605
C	6.0	-1.3569577059	2.7730068914	2.3816692800
C	6.0	1.5184014963	2.6907754817	2.0698358814
C	6.0	-3.6098693646	1.8005528104	1.7643865591
C	6.0	-2.6250443894	2.0679440610	2.7199963158
C	6.0	-2.7723874283	-1.9229143882	0.9673245187
C	6.0	-4.2332313271	0.5543737068	1.6930091893
C	6.0	-3.8946405220	-0.4757226166	2.5753806438
C	6.0	-1.6789944052	-2.7707463060	0.9033162373
C	6.0	-1.3332124830	-3.5917103941	1.9860219467
C	6.0	-3.5927250387	-1.8661839016	2.1095166371
C	6.0	1.1680416755	-3.7221966485	1.4292876503
C	6.0	0.1143723725	-3.6074526816	2.3426155488
C	6.0	2.3421166248	-2.9837593946	1.5503959570
C	6.0	2.5011941327	-1.9707803545	2.5253944066
C	6.0	3.3553814656	-0.7861753334	2.3835369110
C	6.0	3.5157477572	0.1105171084	3.4798573304
C	6.0	0.8908854412	3.3649144026	3.1372514910
C	6.0	3.2748437555	1.4521115362	3.3878410656
C	6.0	-0.4937113652	3.3769177390	3.3002252382

C	6.0	-2.5529359056	1.1600101833	3.7834392245
C	6.0	-3.4473182590	-2.9335721923	3.0001440608
C	6.0	-3.1710046996	-0.0781840076	3.7140706826
C	6.0	-2.3383775742	-3.7790748988	2.9395197652
C	6.0	0.4060483992	-2.8183011742	3.4659661871
C	6.0	1.5324419788	-2.0234858398	3.5493419825
F	9.0	1.5260932500	-1.1504496190	4.5589710977
F	9.0	-0.5323522918	-2.6055727781	4.3961147003
F	9.0	1.0175195111	-4.4957696289	0.3493880672
F	9.0	3.2905852201	-3.1983169602	0.6353959238
F	9.0	3.3773073084	-0.7869782163	-0.0066842145
F	9.0	3.4199328045	1.8171596652	-0.1799553710
F	9.0	1.0368637514	1.6386792533	-0.0381872149
F	9.0	-1.4611936517	1.6887799278	0.2757705330
F	9.0	-3.8158766920	2.6556175513	0.7640475233
F	9.0	-4.9973730943	0.2973861086	0.6314123030
F	9.0	-2.8410740213	-0.9689815628	0.0385100278
F	9.0	-0.8088710477	-2.5660144093	-0.0833196244
F	9.0	3.8162006767	-0.3849376392	4.6894867129
F	9.0	3.3348756877	2.1753263093	4.5110884638
F	9.0	1.6043263718	3.9328944927	4.1005742551
F	9.0	-0.9914966357	3.8936624636	4.4266184200
F	9.0	-1.6707648850	1.3442028476	4.7622630781
F	9.0	-2.8184511132	-0.9709578050	4.6380372538
F	9.0	-4.2420775495	-3.0228566389	4.0668388316
F	9.0	-2.1408563598	-4.6128967276	3.9604363412
I	53.0	4.3224309323	4.2942197318	1.9809645572

(7) Cl^- is on the center of the fluorinated Coronene

C	6.0	1.2961098741	3.5921286618	-0.1476430721
C	6.0	0.0513166140	2.9148105633	-0.1015341170
C	6.0	0.0490024827	1.5028383946	-0.0589461371
C	6.0	1.2818418086	0.7876246880	-0.0697466476
C	6.0	2.5063136202	1.4906320325	-0.1231883409
C	6.0	2.4763700652	2.9077752921	-0.1580971457
C	6.0	-1.1858034111	0.7938280001	0.0045541546
C	6.0	1.2798989111	-0.6367261155	-0.0169737838
C	6.0	0.0450131088	-1.3459028169	0.0465838733
C	6.0	-1.1879013543	-0.6305041735	0.0574221684
C	6.0	0.0431324426	-2.7584484786	0.1046544712
C	6.0	1.2858809920	-3.4413044213	0.1014115171
C	6.0	2.4680109280	-2.7623357608	0.0414472037
C	6.0	2.5024875569	-1.3461727443	-0.0204085353
C	6.0	3.7147401736	-0.6123333228	-0.0708605977
C	6.0	3.7162032503	0.7512449571	-0.1201101543
C	6.0	-1.1905718969	3.5984510072	-0.0732762734
C	6.0	-2.3727261429	2.9200038090	-0.0127363452
C	6.0	-2.4077131507	1.5030323603	0.0246544316
C	6.0	-3.6189203128	0.7698412041	0.1016541494
C	6.0	-3.6213630406	-0.5937454070	0.1513028485
C	6.0	-2.4120121401	-1.3337285094	0.1281392227
C	6.0	-1.2009796025	-3.4350378998	0.1767548903
C	6.0	-2.3810857162	-2.7500568871	0.1882708043
F	9.0	3.6033967469	-3.4671858350	0.0377423852
F	9.0	1.3240686894	-4.7763602060	0.1529454685

F	9.0	-1.2426590384	-4.7699139586	0.2310891788
F	9.0	-3.5181705103	-3.4492614753	0.2534524264
F	9.0	-4.7987157930	-1.2231828712	0.2180705601
F	9.0	-4.7941955739	1.4055425343	0.1227164690
F	9.0	-3.5079994125	3.6252542608	0.0049157266
F	9.0	-1.2282025458	4.9338440483	-0.1117836836
F	9.0	1.3382419980	4.9273068571	-0.1880454808
F	9.0	3.6142886199	3.6071801057	-0.2083023324
F	9.0	4.8938723656	1.3808333137	-0.1712149322
F	9.0	4.8906559039	-1.2479267080	-0.0763734423
CL	17.0	0.1281734903	0.3539555011	2.8880288495

(8) Br^- is on the center of the fluorinated Coronene

C	6.0	1.2961747907	3.5913402131	-0.1481942160
C	6.0	0.0514601436	2.9142150156	-0.1083277650
C	6.0	0.0488297394	1.5015544519	-0.0749815785
C	6.0	1.2816019198	0.7863590306	-0.0859751946
C	6.0	2.5066522265	1.4898203258	-0.1314781697
C	6.0	2.4766960984	2.9067892374	-0.1592924534
C	6.0	-1.1859712832	0.7925257875	-0.0122842059
C	6.0	1.2796195249	-0.6379557609	-0.0330812324
C	6.0	0.0448044470	-1.3471138326	0.0318610278
C	6.0	-1.1880651735	-0.6317132911	0.0417893504
C	6.0	0.0432625673	-2.7596642701	0.0984173421
C	6.0	1.2859624597	-3.4420935157	0.0979416568
C	6.0	2.4682492765	-2.7630331445	0.0359379082
C	6.0	2.5027278507	-1.3473323284	-0.0305112941
C	6.0	3.7147040081	-0.6134143123	-0.0787320868

C	6.0	3.7162336915	0.7505165952	-0.1269070152
C	6.0	-1.1900380350	3.5975976676	-0.0755670990
C	6.0	-2.3725173807	2.9189155504	-0.0168048410
C	6.0	-2.4078964490	1.5021583957	0.0147111904
C	6.0	-3.6186314793	0.7691635502	0.0945328334
C	6.0	-3.6211094971	-0.5946619385	0.1453874535
C	6.0	-2.4122308112	-1.3347767059	0.1201917089
C	6.0	-1.2005360020	-3.4357199633	0.1757851533
C	6.0	-2.3808629187	-2.7505656078	0.1863717281
F	9.0	3.6031861330	-3.4677019601	0.0358599196
F	9.0	1.3244062545	-4.7764169900	0.1553503309
F	9.0	-1.2420365263	-4.7698158803	0.2371167194
F	9.0	-3.5172108024	-3.4493758932	0.2576949399
F	9.0	-4.7978686836	-1.2236568828	0.2161183658
F	9.0	-4.7934454233	1.4045266586	0.1185325261
F	9.0	-3.5070365656	3.6241150855	0.0062292451
F	9.0	-1.2276822096	4.9325836417	-0.1070734129
F	9.0	1.3386305806	4.9261314585	-0.1808149114
F	9.0	3.6142194977	3.6062785489	-0.2024829032
F	9.0	4.8936098487	1.3797820631	-0.1744546174
F	9.0	4.8902703194	-1.2485865229	-0.0822033616
BR	35.0	0.1218378628	0.3852255232	3.0659067363

(9) I^- is on the center of the fluorinated Coronene

C	6.0	1.2956152348	3.5913520732	-0.1627404436
C	6.0	0.0511358077	2.9145678123	-0.1218763496
C	6.0	0.0483017284	1.5010237552	-0.0964773042
C	6.0	1.2810394907	0.7858706871	-0.1070076546

C	6.0	2.5067647656	1.4899427334	-0.1443476541
C	6.0	2.4764665235	2.9064949707	-0.1737969035
C	6.0	-1.1864464946	0.7921381073	-0.0321684225
C	6.0	1.2792617367	-0.6383679432	-0.0520261427
C	6.0	0.0443887314	-1.3474477070	0.0116791559
C	6.0	-1.1884457538	-0.6320916707	0.0216743539
C	6.0	0.0430524338	-2.7599948915	0.0863995252
C	6.0	1.2857070001	-3.4417940074	0.0910127549
C	6.0	2.4684858096	-2.7626655420	0.0314031410
C	6.0	2.5030536284	-1.3474141945	-0.0384568641
C	6.0	3.7146944766	-0.6130900314	-0.0785977977
C	6.0	3.7162789685	0.7511496554	-0.1294225401
C	6.0	-1.1897606354	3.5979848873	-0.0801908050
C	6.0	-2.3725138284	2.9190868362	-0.0188630205
C	6.0	-2.4083708119	1.5025448406	0.0059431804
C	6.0	-3.6185757161	0.7697744077	0.0901716189
C	6.0	-3.6208771601	-0.5945295844	0.1392478541
C	6.0	-2.4127176789	-1.3349355158	0.1086717715
C	6.0	-1.2003980447	-3.4354261820	0.1684670263
C	6.0	-2.3811014676	-2.7502150210	0.1795483722
F	9.0	3.6029334292	-3.4666541395	0.0370740788
F	9.0	1.3243446822	-4.7751718577	0.1516087231
F	9.0	-1.2418685425	-4.7684801763	0.2352564159
F	9.0	-3.5165926961	-3.4482799663	0.2570973550
F	9.0	-4.7967850526	-1.2227257432	0.2143535144
F	9.0	-4.7924214624	1.4049232295	0.1204222822
F	9.0	-3.5057342225	3.6243605495	0.0151400584

F	9.0	-1.2271594449	4.9323748022	-0.1032399490
F	9.0	1.3381925906	4.9252689579	-0.1955826768
F	9.0	3.6131465357	3.6056937189	-0.2168736755
F	9.0	4.8931694255	1.3804243757	-0.1672607606
F	9.0	4.8899018647	-1.2471512791	-0.0695283969
I	53.0	0.1238341485	0.3774590532	3.3298559571

(10) Cl^- is on side of the fluorinated Coronene

C	6.0	1.2425532743	3.5068461189	0.0621132512
C	6.0	0.0004582420	2.8219616806	0.0554698618
C	6.0	0.0004475542	1.4116200658	0.0157944013
C	6.0	1.2347025964	0.6976711045	0.0132829740
C	6.0	2.4600861689	1.4088358573	0.0117604035
C	6.0	2.4242310058	2.8249803740	0.0223004402
C	6.0	-1.2339621687	0.6978230961	0.0141466182
C	6.0	1.2367386513	-0.7283984362	0.0006543148
C	6.0	0.0002562582	-1.4442127576	-0.0016527790
C	6.0	-1.2362152302	-0.7282022466	0.0013264807
C	6.0	0.0001218026	-2.8613409035	-0.0136216283
C	6.0	1.2452820983	-3.5398648303	-0.0184226087
C	6.0	2.4278273227	-2.8565334629	-0.0163645126
C	6.0	2.4643321151	-1.4388914981	-0.0074422833
C	6.0	3.6719499949	-0.6994768880	-0.0111980962
C	6.0	3.6682541129	0.6678739828	-0.0030944343
C	6.0	-1.2416393636	3.5071074678	0.0608334453
C	6.0	-2.4233257890	2.8253582644	0.0212659318
C	6.0	-2.4591959984	1.4092512249	0.0131683000
C	6.0	-3.6674703203	0.6684292140	0.0015450370

C	6.0	-3.6713871267	-0.6989322333	-0.0065266275
C	6.0	-2.4639080249	-1.4385197885	-0.0060565151
C	6.0	-1.2451392793	-3.5396933815	-0.0205455775
C	6.0	-2.4275301317	-2.8561597917	-0.0177908123
F	9.0	3.5630692643	-3.5601450022	-0.0239475206
F	9.0	1.2857501801	-4.8759625203	-0.0278353705
F	9.0	-1.2857754437	-4.8757790534	-0.0336050139
F	9.0	-3.5629053552	-3.5595411914	-0.0282582861
F	9.0	-4.8502750253	-1.3306977654	-0.0171568101
F	9.0	-4.8451804218	1.2948821544	-0.0052823933
F	9.0	-3.5635814593	3.5227325000	0.0174381362
F	9.0	-1.2803256172	4.8301115156	0.0596078942
F	9.0	1.2816753531	4.8298507393	0.0639936712
F	9.0	3.5645899582	3.5222039933	0.0201003482
F	9.0	4.8460475211	1.2942387160	-0.0137636293
F	9.0	4.8507019888	-1.3314300048	-0.0257691118
CL	17.0	-0.0112587197	3.4597981859	2.8586549576

(11) Cl^{-} is in the fluorinated Corannulene

C	6.0	1.2057279930	-0.0000011006	-0.0098557394
C	6.0	0.3726697720	1.1468996524	-0.0103725563
C	6.0	-0.9754729278	0.7087240118	-0.0103129775
C	6.0	-0.9754891326	-0.7087830579	-0.0104049745
C	6.0	0.3726723112	-1.1469022696	-0.0102967029
C	6.0	2.4654987737	0.0000318210	0.5594629228
C	6.0	0.7618963195	2.3447880565	0.5595452316
C	6.0	-1.9945993580	1.4490160113	0.5594054423
C	6.0	-1.9945364998	-1.4490215974	0.5595583691

C	6.0	0.7619527450	-2.3447736867	0.5596083359
C	6.0	2.9494495376	1.2913345452	0.9464873300
C	6.0	-0.3166989340	3.2041965271	0.9462243804
C	6.0	-3.1450935363	0.6890342555	0.9466820255
C	6.0	-1.6272327571	-2.7783906559	0.9462581566
C	6.0	2.1395461372	-2.4062466757	0.9465820129
C	6.0	2.1394683650	2.4062252874	0.9465380509
C	6.0	-1.6272845145	2.7783484723	0.9461688427
C	6.0	-3.1450579514	-0.6890643779	0.9468101873
C	6.0	-0.3166263374	-3.2041975412	0.9462600932
C	6.0	2.9494469090	-1.2912944323	0.9463778117
F	9.0	4.1929720128	1.4181397816	1.4232992850
F	9.0	2.6440937627	3.5496556368	1.4236742466
F	9.0	-0.0531122889	4.4260499954	1.4230221162
F	9.0	-2.5585343437	3.6122298133	1.4227365131
F	9.0	-4.2253630190	1.3175957742	1.4237764439
F	9.0	-4.2253081664	-1.3175869252	1.4239965837
F	9.0	-2.5585916412	-3.6122794343	1.4226303047
F	9.0	-0.0531846240	-4.4261733972	1.4228420168
F	9.0	2.6440971497	-3.5497576223	1.4235844751
F	9.0	4.1930162558	-1.4182559314	1.4230575123
CL	17.0	-0.0003219822	0.0004590337	2.9967256503

(12) Br^- is in the fluorinated Corannulene

C	6.0	1.2057467884	-0.0000150616	-0.0091698775
C	6.0	0.3727614654	1.1468013785	-0.0094660542
C	6.0	-0.9753101221	0.7087570841	-0.0098729563
C	6.0	-0.9752940021	-0.7086981179	-0.0098457053

C	6.0	0.3727614172	-1.1468171841	-0.0096085331
C	6.0	2.4673658085	-0.0000202165	0.5563945596
C	6.0	0.7626389314	2.3464831024	0.5565602809
C	6.0	-1.9959509119	1.4501128470	0.5560988922
C	6.0	-1.9958596363	-1.4501088381	0.5561899609
C	6.0	0.7627097603	-2.3465931734	0.5561871143
C	6.0	2.9522882945	1.2928250421	0.9388232512
C	6.0	-0.3170400075	3.2068740735	0.9396604597
C	6.0	-3.1475578605	0.6893029719	0.9402517007
C	6.0	-1.6282322495	-2.7810655738	0.9387970021
C	6.0	2.1416733870	-2.4080422762	0.9400710620
C	6.0	2.1419542930	2.4082096189	0.9391408603
C	6.0	-1.6282185717	2.7809035641	0.9392379973
C	6.0	-3.1475360996	-0.6893410224	0.9401426537
C	6.0	-0.3170973778	-3.2071525586	0.9386090141
C	6.0	2.9519498315	-1.2926185841	0.9400648249
F	9.0	4.1967606256	1.4210373936	1.4109789725
F	9.0	2.6478885152	3.5520498313	1.4120623522
F	9.0	-0.0543363628	4.4295258192	1.4130684473
F	9.0	-2.5593285650	3.6159331109	1.4122362582
F	9.0	-4.2285314909	1.3172869833	1.4146700590
F	9.0	-4.2283827957	-1.3173166652	1.4148728841
F	9.0	-2.5594423115	-3.6161841280	1.4114483475
F	9.0	-0.0545183895	-4.4300507453	1.4114833618
F	9.0	2.6472896119	-3.5515689729	1.4141229382
F	9.0	4.1958468392	-1.4204817741	1.4138673923
BR	35.0	-0.0069987847	-0.0000278864	3.1829938695

(13) I^- is inside the fluorinated Corannulene

C	6.0	1.2051526013	-0.0000160297	0.0013443955
C	6.0	0.3724540182	1.1462847802	0.0013659546
C	6.0	-0.9750369498	0.7083546793	0.0013616555
C	6.0	-0.9750566596	-0.7084211875	0.0013364365
C	6.0	0.3724458692	-1.1463067325	0.0014690737
C	6.0	2.4720063565	0.0000027784	0.5545349553
C	6.0	0.7639142394	2.3506560623	0.5555065222
C	6.0	-1.9997127528	1.4527030946	0.5553012059
C	6.0	-1.9997168025	-1.4526940217	0.5554126385
C	6.0	0.7638854889	-2.3506426710	0.5556945208
C	6.0	1.2051526013	-0.0000160297	0.0013443955
C	6.0	0.3724540182	1.1462847802	0.0013659546
C	6.0	-0.9750369498	0.7083546793	0.0013616555
C	6.0	-0.9750566596	-0.7084211875	0.0013364365
C	6.0	0.3724458692	-1.1463067325	0.0014690737
C	6.0	2.4720063565	0.0000027784	0.5545349553
C	6.0	0.7639142394	2.3506560623	0.5555065222
C	6.0	-1.9997127528	1.4527030946	0.5553012059
C	6.0	-1.9997168025	-1.4526940217	0.5554126385
C	6.0	0.7638854889	-2.3506426710	0.5556945208
C	6.0	2.9579999848	1.2961021202	0.9281328652
C	6.0	-0.3186193857	3.2137432195	0.9281760858
C	6.0	-3.1548761847	0.6900464919	0.9287599534
C	6.0	-1.6310957720	-2.7872185399	0.9282589188
C	6.0	2.1468330026	-2.4126070791	0.9285730041
C	6.0	2.1467811919	2.4125637381	0.9286767890

C	6.0	-1.6310976908	2.7872421571	0.9280925529
C	6.0	-3.1548889455	-0.6900360635	0.9288506788
C	6.0	-0.3186133884	-3.2137124654	0.9284765942
C	6.0	2.9580330974	-1.2961317806	0.9279683401
F	9.0	4.2047039869	1.4275790129	1.3905400407
F	9.0	2.6567063129	3.5574608955	1.3917710970
F	9.0	-0.0585241323	4.4401427605	1.3903347247
F	9.0	-2.5622807792	3.6267432464	1.3901148169
F	9.0	-4.2404606493	1.3167041345	1.3914236137
F	9.0	-4.2404575369	-1.3166909778	1.3915485298
F	9.0	-2.5622989182	-3.6267420970	1.3902056707
F	9.0	-0.0585041662	-4.4400482364	1.3907885921
F	9.0	2.6567863255	-3.5575461320	1.3915419524
F	9.0	4.2048870370	-1.4277084389	1.3899625184
I	53.0	-0.0013487685	0.0001932514	3.4645466930

(14) Cl^- is outside the fluorinated Corannulene

C	6.0	1.2048184717	-0.0000049184	0.0617412411
C	6.0	0.3723141093	1.1461542693	0.0616757004
C	6.0	-0.9749352195	0.7082854363	0.0620209407
C	6.0	-0.9749597271	-0.7083161483	0.0617954843
C	6.0	0.3723065975	-1.1461514857	0.0617189809
C	6.0	2.4798662061	0.0000281459	0.5851658897
C	6.0	0.7663644146	2.3586772752	0.5854435089
C	6.0	-2.0062985329	1.4575319829	0.5862011540
C	6.0	-2.0063820176	-1.4576565008	0.5857301978
C	6.0	0.7662865616	-2.3586065036	0.5856918007
C	6.0	2.9723231118	1.3066055510	0.9342960297

C	6.0	-0.3240146260	3.2307767218	0.9347253938
C	6.0	-3.1726607188	0.6900761847	0.9354693586
C	6.0	-1.6369457794	-2.8043385900	0.9344355375
C	6.0	2.1609758895	-2.4232873727	0.9350654907
C	6.0	2.1611612822	2.4234640754	0.9344594398
C	6.0	-1.6367547520	2.8041272575	0.9351082949
C	6.0	-3.1727731478	-0.6903005404	0.9350456736
C	6.0	-0.3241381200	-3.2308192638	0.9346112647
C	6.0	2.9721676352	-1.3064692504	0.9347388446
F	9.0	4.2319229817	1.4451826085	1.3680843906
F	9.0	2.6819768336	3.5784343776	1.3687501575
F	9.0	-0.0667436213	4.4716418988	1.3682462459
F	9.0	-2.5740255220	3.6567488352	1.3692148024
F	9.0	-4.2729462976	1.3183645495	1.3696164432
F	9.0	-4.2732697958	-1.3185694414	1.3687422683
F	9.0	-2.5743161129	-3.6571574586	1.3679401908
F	9.0	-0.0668556014	-4.4716052090	1.3682987625
F	9.0	2.6816759068	-3.5781450289	1.3698144517
F	9.0	4.2316567699	-1.4449842483	1.3688456776
CL	17.0	0.0022028504	0.0003127603	-2.8260735273

(15) Cl^- is side the fluorinated Corannulene

C	6.0	1.1220194004	-0.0024583043	0.0341007843
C	6.0	0.2791043042	1.1336119877	0.0180327815
C	6.0	-1.0579219480	0.6745883783	0.0743083802
C	6.0	-1.0425869650	-0.7409359615	0.0990768079
C	6.0	0.3087373607	-1.1606661744	0.0740635258
C	6.0	2.4122399927	0.0150039996	0.5357589513

C	6.0	0.6757061074	2.3834893947	0.4658345461
C	6.0	-2.0936167284	1.4145259401	0.6189478662
C	6.0	-2.0625588348	-1.5082760642	0.6366571140
C	6.0	0.7322163529	-2.3764017042	0.5847167464
C	6.0	2.8840771919	1.3326599124	0.8763550436
C	6.0	-0.4228515013	3.2158961606	0.9035049623
C	6.0	-3.2280577037	0.6328274821	1.0074592376
C	6.0	-1.6639287916	-2.8496639335	0.9719105710
C	6.0	2.1245822186	-2.4155285894	0.9106622726
C	6.0	2.0663948750	2.4426677848	0.8581687214
C	6.0	-1.7243396530	2.7643052882	0.9607834191
C	6.0	-3.2202537367	-0.7555031848	1.0101916207
C	6.0	-0.3442228600	-3.2595531014	0.9474335318
C	6.0	2.9171650894	-1.2758100748	0.8932855896
F	9.0	4.1409347431	1.4786885361	1.3209178211
F	9.0	2.5672782208	3.5825000718	1.3137174874
F	9.0	-0.1747600449	4.4343482063	1.3633514229
F	9.0	-2.6614323023	3.5926923343	1.4448552846
F	9.0	-4.3205678085	1.2385746041	1.4866138863
F	9.0	-4.3050832972	-1.3867286906	1.4819989759
F	9.0	-2.5787804943	-3.7195668852	1.4185024832
F	9.0	-0.0680849122	-4.4991323044	1.3719192588
F	9.0	2.6770865017	-3.5549006308	1.3516855711
F	9.0	4.1763521680	-1.4005555056	1.3281076168
CL	17.0	1.1476161751	4.0263868279	-1.5463907713

(16) Cl^- is on the center of the flat fluorinated Corannulene

C	6.0	1.1869616911	0.0000071086	0.0000000000

C	6.0	0.3668251690	1.1292696701	0.0000000000
C	6.0	-0.9604480973	0.6978213615	0.0000000000
C	6.0	-0.9604447857	-0.6978238368	0.0000000000
C	6.0	0.3668276475	-1.1292641094	0.0000000000
C	6.0	2.5504964676	0.0000000366	0.0000000000
C	6.0	0.7879715213	2.4262635797	0.0000000000
C	6.0	-2.0637414023	1.4992181624	0.0000000000
C	6.0	-2.0637415838	-1.4992137096	0.0000000000
C	6.0	0.7879685570	-2.4262624069	0.0000000000
C	6.0	3.0548489397	1.3565855421	0.0000000000
C	6.0	-0.3463293301	3.3250976469	0.0000000000
C	6.0	-3.2692251712	0.6984788930	0.0000000000
C	6.0	-1.6748398845	-2.8931911750	0.0000000000
C	6.0	2.2339474501	-2.4868858615	0.0000000000
C	6.0	2.2339504507	2.4868867485	0.0000000000
C	6.0	-1.6748371092	2.8931938079	0.0000000000
C	6.0	-3.2692260097	-0.6984763904	0.0000000000
C	6.0	-0.3463323789	-3.3250946050	0.0000000000
C	6.0	3.0548465781	-1.3565860805	0.0000000000
F	9.0	-0.1440164334	-4.6492331524	0.0000000000
F	9.0	2.8485430801	-3.6770953623	0.0000000000
F	9.0	4.3767296498	-1.5736496046	0.0000000000
F	9.0	4.3767328636	1.5736411348	0.0000000000
F	9.0	2.8485555051	3.6770932283	0.0000000000
F	9.0	-0.1440151448	4.6492362009	0.0000000000
F	9.0	-2.6168981193	3.8454684284	0.0000000000
F	9.0	-4.4660493038	1.3001027930	0.0000000000

F	9.0	-4.4660548587	-1.3000927104	0.0000000000
F	9.0	-2.6169004754	-3.8454643135	0.0000000000
CL	17.0	0.0078945169	-0.0000310375	2.9101727401

(17) Cl^- is on side of the flat fluorinated Corannulene

C	6.0	1.1783691691	0.0651141191	0.0000000000
C	6.0	0.3392021562	1.1806483005	0.0000000000
C	6.0	-0.9814433846	0.7317519151	0.0000000000
C	6.0	-0.9603218877	-0.6663595378	0.0000000000
C	6.0	0.3732571081	-1.0751813346	0.0000000000
C	6.0	2.5417624707	0.0856462148	0.0000000000
C	6.0	0.7439929247	2.4877786047	0.0000000000
C	6.0	-2.1015292505	1.5194646257	0.0000000000
C	6.0	-2.0560406419	-1.4876071324	0.0000000000
C	6.0	0.8172733882	-2.3694699363	0.0000000000
C	6.0	3.0296339410	1.4526152471	0.0000000000
C	6.0	-0.4042883996	3.3640205286	0.0000000000
C	6.0	-3.2943715607	0.6963981049	0.0000000000
C	6.0	-1.6455964730	-2.8710010330	0.0000000000
C	6.0	2.2647673917	-2.4074307438	0.0000000000
C	6.0	2.1897264142	2.5692172216	0.0000000000
C	6.0	-1.7331788809	2.9145524335	0.0000000000
C	6.0	-3.2731887298	-0.7012572111	0.0000000000
C	6.0	-0.3037848068	-3.2801148072	0.0000000000
C	6.0	3.0705539076	-1.2659461385	0.0000000000
F	9.0	-0.0898216961	-4.6006802016	0.0000000000
F	9.0	2.8899406413	-3.5932840899	0.0000000000
F	9.0	4.3802428140	-1.4649916065	0.0000000000

F	9.0	4.3326255208	1.6914042840	0.0000000000
F	9.0	2.7782402603	3.7735849080	0.0000000000
F	9.0	-0.2308943351	4.6905772878	0.0000000000
F	9.0	-2.6869768910	3.8564476772	0.0000000000
F	9.0	-4.5010466011	1.2777171685	0.0000000000
F	9.0	-4.4613886110	-1.3194064794	0.0000000000
F	9.0	-2.5700757422	-3.8417245899	0.0000000000
CL	17.0	3.3815775422	0.0971619510	2.7002130259

(18) fluorinated [5] cycloparaphenylene

C	6.0	3.6105736957	0.0475890623	1.1335281645
C	6.0	0.7666791684	2.5367054804	1.0177912763
C	6.0	3.4162652870	1.4225199077	1.0365241049
C	6.0	2.9259970990	2.1456505273	2.1415786451
C	6.0	-0.6021768839	2.6811127835	1.1607227899
C	6.0	-1.1772527408	3.1824481693	2.3319485557
C	6.0	1.6785992360	2.9574611735	2.0017118273
C	6.0	-3.4263854030	2.1806772745	1.7637503064
C	6.0	-2.4416897388	2.4790237475	2.7096709322
C	6.0	-2.4300482085	-1.5457438892	0.9137581738
C	6.0	-3.9775759341	0.9001440388	1.6725425644
C	6.0	-3.5723864785	-0.1223191290	2.5332431750
C	6.0	-1.3706413712	-2.4341143138	0.8202693663
C	6.0	-1.0743016300	-3.3224265896	1.8636363760
C	6.0	-3.2485762598	-1.5028134221	2.0563831723
C	6.0	1.4446040115	-3.5358101162	1.3404782641
C	6.0	0.3748929675	-3.3802837327	2.2285488133
C	6.0	2.6534472756	-2.8536493921	1.5106422125

C	6.0	2.8275549819	-1.8942554227	2.5271585774
C	6.0	3.5787082349	-0.6111552920	2.3745303266
C	6.0	3.7052113807	0.2572004233	3.4757791247
C	6.0	1.1028087263	3.7347255360	3.0260297990
C	6.0	3.2626667590	1.5737389376	3.3802448160
C	6.0	-0.2816749338	3.8003593587	3.2114784761
C	6.0	-2.2976792256	1.5538209902	3.7529839033
C	6.0	-3.1445436635	-2.5962595865	2.9188426554
C	6.0	-2.8483790944	0.2850050229	3.6677463442
C	6.0	-2.0785720861	-3.4940174584	2.8201310909
C	6.0	0.6727532452	-2.6647839665	3.3917416256
C	6.0	1.8313607363	-1.9190732269	3.5189187467
F	9.0	1.8082278870	-1.0524430924	4.5285225761
F	9.0	-0.2631651146	-2.4354762958	4.3107057521
F	9.0	1.2858828399	-4.2533201156	0.2332715179
F	9.0	3.5972759585	-3.0472679839	0.5966091357
F	9.0	3.6220468263	-0.6308861060	-0.0092232632
F	9.0	3.4691076746	1.9745548086	-0.1692311140
F	9.0	1.1243672767	1.7570026751	0.0004398693
F	9.0	-1.3447616354	2.0582122829	0.2477015296
F	9.0	-3.6727043948	3.0345389528	0.7761556416
F	9.0	-4.7214922071	0.6165185175	0.6093134244
F	9.0	-2.4689923210	-0.5595487030	0.0201080375
F	9.0	-0.4776854967	-2.2241994199	-0.1449184270
F	9.0	4.0093734364	-0.2110388595	4.6798232044
F	9.0	2.9802272475	2.1866254479	4.5251319664
F	9.0	1.8686279850	4.3311117569	3.9324904671

F	9.0	-0.7335150788	4.3678552743	4.3248753072
F	9.0	-1.3956964923	1.7675288686	4.7088042194
F	9.0	-2.4331224907	-0.6120780052	4.5596419554
F	9.0	-3.9261438125	-2.6730513781	3.9902146603
F	9.0	-1.9054581710	-4.3628012608	3.8103506843

(19) fluorinated Coronene

C	6.0	1.2447088268	3.5231730611	0.0000000000
C	6.0	0.0000407060	2.8426585474	0.0000000000
C	6.0	0.0000382519	1.4265502059	0.0000000000
C	6.0	1.2359696605	0.7135969399	0.0000000000
C	6.0	2.4625554726	1.4215563876	-0.0000000000
C	6.0	2.4292192753	2.8397005472	0.0000000000
C	6.0	-1.2359284197	0.7136132227	0.0000000000
C	6.0	1.2357550320	-0.7133024284	0.0000000000
C	6.0	0.0000336005	-1.4267034154	0.0000000000
C	6.0	-1.2357729650	-0.7133014198	0.0000000000
C	6.0	0.0000048882	-2.8428965001	-0.0000000000
C	6.0	1.2448098126	-3.5231547320	0.0000000000
C	6.0	2.4293215261	-2.8397262204	0.0000000000
C	6.0	2.4621639757	-1.4215985464	-0.0000000000
C	6.0	3.6738213295	-0.6838659568	0.0000000000
C	6.0	3.6741082299	0.6836690137	-0.0000000000
C	6.0	-1.2447439986	3.5231104439	-0.0000000000
C	6.0	-2.4292899083	2.8397464345	0.0000000000
C	6.0	-2.4625140586	1.4215897435	0.0000000000
C	6.0	-3.6740605767	0.6837019162	0.0000000000
C	6.0	-3.6738454732	-0.6838328896	0.0000000000

C	6.0	-2.4621789593	-1.4215634274	0.0000000000
C	6.0	-1.2448131609	-3.5231493281	0.0000000000
C	6.0	-2.4292973099	-2.8396916549	-0.0000000000
F	9.0	3.5608998080	-3.5366587237	-0.0000000000
F	9.0	1.2828196304	-4.8516693002	0.0000000000
F	9.0	-1.2828096553	-4.8516698730	0.0000000000
F	9.0	-3.5608724033	-3.5366275853	0.0000000000
F	9.0	-4.8434679076	-1.3149917600	0.0000000000
F	9.0	-4.8436716190	1.3148559995	-0.0000000000
F	9.0	-3.5607724366	3.5368417211	-0.0000000000
F	9.0	-1.2825812576	4.8516529220	0.0000000000
F	9.0	1.2825439463	4.8516946310	-0.0000000000
F	9.0	3.5606568487	3.5368899248	0.0000000000
F	9.0	4.8437095446	1.3148357104	-0.0000000000
F	9.0	4.8434397441	-1.3150336109	0.0000000000

(20) fluorinated Corannulene

C	6.0	1.2037844486	-0.0000038661	0.0701227374
C	6.0	0.3721059681	1.1451050849	0.0697151515
C	6.0	-0.9738771455	0.7076304818	0.0698511394
C	6.0	-0.9739431795	-0.7076557967	0.0698312848
C	6.0	0.3720629613	-1.1450669325	0.0698243029
C	6.0	2.4841703527	0.0000004577	0.5893066865
C	6.0	0.7677165768	2.3626806711	0.5894421571
C	6.0	-2.0096094689	1.4600342074	0.5896717979
C	6.0	-2.0096976844	-1.4600408182	0.5896335069
C	6.0	0.7676696303	-2.3626687130	0.5895007359
C	6.0	2.9753241636	1.3053426245	0.9381921024

C	6.0	-0.3219758624	3.2332849175	0.9379278880
C	6.0	-3.1743258710	0.6929720933	0.9385453596
C	6.0	-1.6399256481	-2.8049619278	0.9380461139
C	6.0	2.1608926676	-2.4266470522	0.9381706942
C	6.0	2.1609013690	2.4265825554	0.9382152536
C	6.0	-1.6399451031	2.8050269895	0.9379775114
C	6.0	-3.1743462357	-0.6928587616	0.9384830540
C	6.0	-0.3219917843	-3.2332941431	0.9380583266
C	6.0	2.9752733962	-1.3053617013	0.9381495491
F	9.0	4.2237949307	1.4432471033	1.3762156897
F	9.0	2.6777108578	3.5712463120	1.3764978675
F	9.0	-0.0675018479	4.4634568873	1.3755844415
F	9.0	-2.5687499007	3.6508385260	1.3756543305
F	9.0	-4.2653693692	1.3152206008	1.3767117979
F	9.0	-4.2654043310	-1.3151884930	1.3764327599
F	9.0	-2.5687321349	-3.6507166498	1.3757359125
F	9.0	-0.0674806194	-4.4633875132	1.3759018958
F	9.0	2.6776996201	-3.5713615896	1.3764929987
F	9.0	4.2237692733	-1.4434556306	1.3761783428

(21) flat fluorinated Corannulene

C	6.0	1.1874264467	0.0000080887	0.0000000000
C	6.0	0.3671465454	1.1294619145	0.0000000000
C	6.0	-0.9603781039	0.6979491373	0.0000000000
C	6.0	-0.9603757274	-0.6979541306	0.0000000000
C	6.0	0.3671495221	-1.1294550359	0.0000000000
C	6.0	2.5541055911	-0.0000027812	0.0000000000
C	6.0	0.7893151636	2.4294190456	0.0000000000

C	6.0	-2.0661835899	1.5012091834	0.0000000000
C	6.0	-2.0661846906	-1.5012108561	0.0000000000
C	6.0	0.7893125330	-2.4294137990	0.0000000000
C	6.0	3.0601575864	1.3573345183	0.0000000000
C	6.0	-0.3452277326	3.3300734216	0.0000000000
C	6.0	-3.2734635263	0.7007579489	0.0000000000
C	6.0	-1.6780889983	-2.8968252967	0.0000000000
C	6.0	2.2365850374	-2.4913644219	0.0000000000
C	6.0	2.2365882780	2.4913585997	0.0000000000
C	6.0	-1.6780939746	2.8968219841	0.0000000000
C	6.0	-3.2734614440	-0.7007547686	0.0000000000
C	6.0	-0.3452221453	-3.3300725680	0.0000000000
C	6.0	3.0601543649	-1.3573409389	0.0000000000
F	9.0	-0.1433831496	-4.6453897133	0.0000000000
F	9.0	2.8460349216	-3.6742780814	0.0000000000
F	9.0	4.3734944254	-1.5720333779	0.0000000000
F	9.0	4.3734971639	1.5720241840	0.0000000000
F	9.0	2.8460457714	3.6742696647	0.0000000000
F	9.0	-0.1433764420	4.6453867887	0.0000000000
F	9.0	-2.6148412368	3.8419157410	0.0000000000
F	9.0	-4.4619418372	1.2993528365	0.0000000000
F	9.0	-4.4619436732	-1.2993434336	0.0000000000
F	9.0	-2.6148470792	-3.8419038838	0.0000000000

5. The xyz coordinates of complexes and monomers optimized at M062X-D3/cc-pVTZ level of theory (in Å) with the arometic ring planished.

(1) Cl^{-}···C6F6

C	6.0	0.0006334837	1.3806223133	0.0000000000

C	6.0	-1.1935078698	0.6893168312	0.0000000000
C	6.0	-1.1920500007	-0.6904518441	0.0000000000
C	6.0	0.0036277006	-1.3790843710	0.0000000000
C	6.0	1.1978161726	-0.6878489121	0.0000000000
C	6.0	1.1962800598	0.6919209702	0.0000000000
F	9.0	0.0050925892	-2.7100330201	0.0000000000
F	9.0	-2.3440540233	-1.3571362152	0.0000000000
F	9.0	-2.3469907249	1.3534730633	0.0000000000
F	9.0	-0.0007724089	2.7115385156	0.0000000000
F	9.0	2.3483388562	1.3585100317	0.0000000000
F	9.0	2.3512955593	-1.3519722257	0.0000000000
CL	17.0	0.0042906062	0.0011438632	3.1072052392

(2) $Br^{-}\cdots C_6F_6$

C	6.0	0.0006371459	1.3811088833	0.0000000000
C	6.0	-1.1940048016	0.6896161801	0.0000000000
C	6.0	-1.1925752458	-0.6906798186	0.0000000000
C	6.0	0.0035131975	-1.3796468390	0.0000000000
C	6.0	1.1981698392	-0.6881797498	0.0000000000
C	6.0	1.1967228738	0.6921160212	0.0000000000
F	9.0	0.0050303840	-2.7096715409	0.0000000000
F	9.0	-2.3437427039	-1.3569409739	0.0000000000
F	9.0	-2.3467251383	1.3532265204	0.0000000000
F	9.0	-0.0008926885	2.7111161502	0.0000000000
F	9.0	2.3479361441	1.3583578934	0.0000000000
F	9.0	2.3509108705	-1.3517666747	0.0000000000
BR	35.0	0.0005911230	0.0001579483	3.3011170315

(3) $I^{-}\cdots C_6F_6$

C	6.0	0.0006010450	1.3815363954	0.0000000000
C	6.0	-1.1943347369	0.6897784710	0.0000000000
C	6.0	-1.1929219890	-0.6909018910	0.0000000000
C	6.0	0.0035366317	-1.3800193209	0.0000000000
C	6.0	1.1985327325	-0.6883491495	0.0000000000
C	6.0	1.1970277384	0.6923421492	0.0000000000
F	9.0	0.0050584598	-2.7094081632	0.0000000000
F	9.0	-2.3435316852	-1.3568599108	0.0000000000
F	9.0	-2.3464746267	1.3531313977	0.0000000000
F	9.0	-0.0008238475	2.7109022941	0.0000000000
F	9.0	2.3477331022	1.3582072665	0.0000000000
F	9.0	2.3507023145	-1.3516640252	0.0000000000
I	53.0	0.0004658611	0.0001184866	3.5230819895

(4) $Cl^{-}\cdots C_6H_3(NO_2)_3$

C	6.0	0.9889095460	0.9888859058	-0.4216000000
C	6.0	-0.3514263889	1.3117679982	-0.4216000000
C	6.0	-1.3507579223	0.3619841212	-0.4216000000
C	6.0	-0.9602024860	-0.9602251703	-0.4216000000
C	6.0	0.3620062402	-1.3507832303	-0.4216000000
C	6.0	1.3117906279	-0.3514536384	-0.4216000000
H	1.0	1.7506048128	1.7505841452	-0.4216000000
H	1.0	0.6407898603	-2.3912790209	-0.4216000000
H	1.0	-2.3912540297	0.6407639905	-0.4216000000
N	7.0	-2.0000959964	-2.0001092498	-0.4216000000
O	8.0	-3.1547509558	-1.6371083945	-0.4216000000
O	8.0	-1.6371018480	-3.1547663232	-0.4216000000
N	7.0	-0.7320396866	2.7323058735	-0.4216000000

O	8.0	-1.9135066906	2.9952216785	-0.4216000000
O	8.0	0.1597691418	3.5506556657	-0.4216000000
N	7.0	2.7323320929	-0.7320604345	-0.4216000000
O	8.0	3.5506674170	0.1597627359	-0.4216000000
O	8.0	2.9952463309	-1.9135261586	-0.4216000000
CL	17.0	-0.0009800656	-0.0006204940	2.6038443350

(5) $Br^{-}\cdots C_6H_3(NO_2)_3$

C	6.0	0.9892550341	0.9892419497	-0.4216000000
C	6.0	-0.3513913067	1.3120609189	-0.4216000000
C	6.0	-1.3510177790	0.3621756688	-0.4216000000
C	6.0	-0.9602494506	-0.9602604292	-0.4216000000
C	6.0	0.3621848808	-1.3510345067	-0.4216000000
C	6.0	1.3120673665	-0.3514075575	-0.4216000000
H	1.0	1.7510619457	1.7510458896	-0.4216000000
H	1.0	0.6410189356	-2.3916781605	-0.4216000000
H	1.0	-2.3916636696	0.6409980216	-0.4216000000
N	7.0	-2.0009064112	-2.0009101116	-0.4216000000
O	8.0	-3.1548104598	-1.6368208520	-0.4216000000
O	8.0	-1.6368248464	-3.1548168708	-0.4216000000
N	7.0	-0.7323206156	2.7336475750	-0.4216000000
O	8.0	-1.9137016905	2.9951384724	-0.4216000000
O	8.0	0.1601020243	3.5507495736	-0.4216000000
N	7.0	2.7336519893	-0.7323273526	-0.4216000000
O	8.0	3.5507626122	0.1600887124	-0.4216000000
O	8.0	2.9951704683	-1.9137015566	-0.4216000000
BR	35.0	-0.0023890276	-0.0021893845	2.7961849505

(6) $I^{-}\cdots C_6H_3(NO_2)_3$

C	6.0	0.9897364675	0.9897365922	-0.4216000000
C	6.0	-0.3511281613	1.3126772547	-0.4216000000
C	6.0	-1.3508997635	0.3625488454	-0.4216000000
C	6.0	-0.9601430529	-0.9601428703	-0.4216000000
C	6.0	0.3625486881	-1.3508996204	-0.4216000000
C	6.0	1.3126771459	-0.3511280297	-0.4216000000
H	1.0	1.7515883469	1.7515885504	-0.4216000000
H	1.0	0.6414062416	-2.3916118491	-0.4216000000
H	1.0	-2.3916119858	0.6414064267	-0.4216000000
N	7.0	-2.0010337908	-2.0010338299	-0.4216000000
O	8.0	-3.1543899855	-1.6360461379	-0.4216000000
O	8.0	-1.6360462649	-3.1543900280	-0.4216000000
N	7.0	-0.7321068822	2.7345587950	-0.4216000000
O	8.0	-1.9134381638	2.9951304720	-0.4216000000
O	8.0	0.1607376489	3.5508570169	-0.4216000000
N	7.0	2.7345587446	-0.7321068682	-0.4216000000
O	8.0	3.5508566439	0.1607379614	-0.4216000000
O	8.0	2.9951300825	-1.9134381915	-0.4216000000
I	53.0	-0.0084419590	-0.0084444898	3.0166619538

(7) $Cl^{-}\cdots C_6H_3(CN)_3$

C	6.0	0.9844000921	0.9844156868	-0.5636842100
C	6.0	1.3360011801	-0.3580079249	-0.5636842100
C	6.0	0.3602942090	-1.3447593175	-0.5636842100
C	6.0	-0.9780914864	-0.9780829742	-0.5636842100
C	6.0	-1.3447748188	0.3602933432	-0.5636842100
C	6.0	-0.3580203501	1.3360109176	-0.5636842100
H	1.0	1.7476902027	1.7477021139	-0.5636842100

H	1.0	-2.3874392578	0.6396843659	-0.5636842100
H	1.0	0.6396757227	-2.3874263726	-0.5636842100
C	6.0	-0.7291502610	2.7209115879	-0.5636842100
N	7.0	-1.0261240744	3.8296176496	-0.5636842100
C	6.0	2.7209068282	-0.7291222789	-0.5636842100
N	7.0	3.8296001532	-1.0261641667	-0.5636842100
C	6.0	-1.9919040978	-1.9919068121	-0.5636842100
N	7.0	-2.8035166998	-2.8035374376	-0.5636842100
CL	17.0	0.0004526581	0.0003716196	2.5034267559

(8) $Br^{-}\cdots C_6H_3(CN)_3$

C	6.0	0.9847495223	0.9847640950	-0.5636842100
C	6.0	1.3364304426	-0.3580101385	-0.5636842100
C	6.0	0.3604729374	-1.3449979859	-0.5636842100
C	6.0	-0.9782486595	-0.9782356790	-0.5636842100
C	6.0	-1.3450057041	0.3604760489	-0.5636842100
C	6.0	-0.3580205482	1.3364438801	-0.5636842100
H	1.0	1.7480545419	1.7480530722	-0.5636842100
H	1.0	-2.3876719353	0.6398993438	-0.5636842100
H	1.0	0.6399047817	-2.3876615640	-0.5636842100
C	6.0	-0.7291290914	2.7215507760	-0.5636842100
N	7.0	-1.0263136021	3.8300460572	-0.5636842100
C	6.0	2.7215403480	-0.7291168595	-0.5636842100
N	7.0	3.8300353875	-1.0262996362	-0.5636842100
C	6.0	-1.9922000107	-1.9922036842	-0.5636842100
N	7.0	-2.8037221515	-2.8036855962	-0.5636842100
BR	35.0	-0.0008762587	-0.0010221296	2.7084356276

(9) $I^{-}\cdots C_6H_3(CN)_3$

C	6.0	0.9851724144	0.9851726659	-0.5636842100
C	6.0	1.3368484811	-0.3579088919	-0.5636842100
C	6.0	0.3607415659	-1.3451809717	-0.5636842100
C	6.0	-0.9782614042	-0.9782615905	-0.5636842100
C	6.0	-1.3451810814	0.3607412869	-0.5636842100
C	6.0	-0.3579092052	1.3368484316	-0.5636842100
H	1.0	1.7484563172	1.7484567511	-0.5636842100
H	1.0	-2.3878054018	0.6401534101	-0.5636842100
H	1.0	0.6401539421	-2.3878052235	-0.5636842100
C	6.0	-0.7291408327	2.7220212797	-0.5636842100
N	7.0	-1.0262263980	3.8304173102	-0.5636842100
C	6.0	2.7220215384	-0.7291397365	-0.5636842100
N	7.0	3.8304168757	-1.0262278890	-0.5636842100
C	6.0	-1.9922915331	-1.9922921927	-0.5636842100
N	7.0	-2.8037141894	-2.8037135637	-0.5636842100
I	53.0	-0.0032810892	-0.0032810759	2.9392222765

(10) C_6F_6

C	6.0	0.0007211809	1.3853177688	0.0000000000
C	6.0	-1.1975586525	0.6917575964	0.0000000000
C	6.0	-1.1962336803	-0.6927132682	0.0000000000
C	6.0	0.0034751650	-1.3837816021	0.0000000000
C	6.0	1.2018279902	-0.6903277389	0.0000000000
C	6.0	1.2004036287	0.6941630702	0.0000000000
F	9.0	0.0052106572	-2.7082567203	0.0000000000
F	9.0	-2.3425254668	-1.3563538991	0.0000000000
F	9.0	-2.3454822755	1.3526134922	0.0000000000
F	9.0	-0.0007868264	2.7097641623	0.0000000000

F	9.0	2.3467866172	1.3577080574	0.0000000000
F	9.0	2.3498371153	-1.3510352438	0.0000000000

(11) $C_6H_3(NO_2)_3$

C	6.0	0.9909813586	0.9909719733	-0.4216000000
C	6.0	-0.3519982860	1.3136290811	-0.4216000000
C	6.0	-1.3537026603	0.3627237824	-0.4216000000
C	6.0	-0.9616650883	-0.9616813038	-0.4216000000
C	6.0	0.3627208423	-1.3537022400	-0.4216000000
C	6.0	1.3136456463	-0.3519979679	-0.4216000000
H	1.0	1.7540288778	1.7540369319	-0.4216000000
H	1.0	0.6420151259	-2.3960591490	-0.4216000000
H	1.0	-2.3960590708	0.6420243836	-0.4216000000
N	7.0	-2.0088504135	-2.0088487131	-0.4216000000
O	8.0	-3.1560720359	-1.6346042648	-0.4216000000
O	8.0	-1.6346306469	-3.1560715624	-0.4216000000
N	7.0	-0.7352831781	2.7441463162	-0.4216000000
O	8.0	-1.9159169338	2.9936428229	-0.4216000000
O	8.0	0.1624702666	3.5504941893	-0.4216000000
N	7.0	2.7441577582	-0.7352685440	-0.4216000000
O	8.0	3.5505162865	0.1624893917	-0.4216000000
O	8.0	2.9936421514	-1.9159251275	-0.4216000000

(12) $C_6H_3(CN)_3$

C	6.0	0.9867008171	0.9866818296	-0.5636842100
C	6.0	1.3390237700	-0.3588127525	-0.5636842100
C	6.0	0.3611338116	-1.3478403976	-0.5636842100
C	6.0	-0.9802734518	-0.9802654094	-0.5636842100
C	6.0	-1.3478293820	0.3611438820	-0.5636842100

C	6.0	-0.3587881282	1.3390245556	-0.5636842100
H	1.0	1.7505414073	1.7505115163	-0.5636842100
H	1.0	-2.3912403634	0.6407618320	-0.5636842100
H	1.0	0.6407344562	-2.3912557967	-0.5636842100
C	6.0	-0.7301694475	2.7249888872	-0.5636842100
N	7.0	-1.0269152906	3.8326325860	-0.5636842100
C	6.0	2.7249765101	-0.7302417761	-0.5636842100
N	7.0	3.8326626361	-1.0268004474	-0.5636842100
C	6.0	-1.9948687184	-1.9948481139	-0.5636842100
N	7.0	-2.8056886265	-2.8056803954	-0.5636842100

6. The xyz coordinates of complexs optimized at BLW(M062x-D3)/cc-pVTZ level of theory with the π electrons on Fluorine atoms localized (in Å).

(1) $Cl^{-}\cdots C_6F_6$

C	6.0	0.0000000000	0.0006357402	1.3734773984
C	6.0	0.0000000000	-1.1872574236	0.6857087674
C	6.0	0.0000000000	-1.1857342450	-0.6868887778
C	6.0	0.0000000000	0.0037349187	-1.3719175888
C	6.0	0.0000000000	1.1916599356	-0.6841997644
C	6.0	0.0000000000	1.1900828177	0.6883965471
F	9.0	0.0000000000	0.0051133122	-2.7372246571
F	9.0	0.0000000000	-2.3675823178	-1.3706941722
F	9.0	0.0000000000	-2.3705275569	1.3670738914
F	9.0	0.0000000000	-0.0007429398	2.7387642015
F	9.0	0.0000000000	2.3719630074	1.3721445923
F	9.0	0.0000000000	2.3749222959	-1.3655579638
CL	17.0	3.0446326335	0.0037324554	0.0009165259

(2) $Br^{-}\cdots C_6F_6$

C	6.0	0.0000000000	0.0005739149	1.3738771211
C	6.0	0.0000000000	-1.1876894106	0.6859471403
C	6.0	0.0000000000	-1.1862535290	-0.6870487568
C	6.0	0.0000000000	0.0035249142	-1.3723511009
C	6.0	0.0000000000	1.1918426486	-0.6844914788
C	6.0	0.0000000000	1.1903324980	0.6885154225
F	9.0	0.0000000000	0.0050595647	-2.7369036915
F	9.0	0.0000000000	-2.3673788557	-1.3705953560
F	9.0	0.0000000000	-2.3703559089	1.3668544182
F	9.0	0.0000000000	-0.0008622491	2.7384172287
F	9.0	0.0000000000	2.3715408308	1.3719709668
F	9.0	0.0000000000	2.3745384575	-1.3653661387
BR	35.0	3.2337699914	0.0006981250	-0.0000117749

(3) $I^{-}\cdots C_6F_6$

C	6.0	0.0000000000	0.0005927834	1.3741254864
C	6.0	0.0000000000	-1.1879260915	0.6860904011
C	6.0	0.0000000000	-1.1864706468	-0.6871816700
C	6.0	0.0000000000	0.0035337242	-1.3726280824
C	6.0	0.0000000000	1.1920728617	-0.6846281149
C	6.0	0.0000000000	1.1905900654	0.6886458101
F	9.0	0.0000000000	0.0050277076	-2.7367413759
F	9.0	0.0000000000	-2.3672423591	-1.3704591260
F	9.0	0.0000000000	-2.3702284051	1.3667456131
F	9.0	0.0000000000	-0.0009102967	2.7382281332
F	9.0	0.0000000000	2.3713993712	1.3718983124
F	9.0	0.0000000000	2.3743890885	-1.3652694212
I	53.0	3.4528983425	0.0007431971	-0.0000119659

(4) $Cl^{-}\cdots C_6H_3(NO_2)_3$

C	6.0	0.0000000000	0.9899342603	0.9892759353
C	6.0	0.0000000000	-0.3478769534	1.2983920787
C	6.0	0.0000000000	-1.3518894468	0.3618059271
C	6.0	0.0000000000	-0.9506788740	-0.9513307053
C	6.0	0.0000000000	0.3624580871	-1.3525644402
C	6.0	0.0000000000	1.2990665348	-0.3485378225
H	1.0	0.0000000000	1.7516620012	1.7510763867
H	1.0	0.0000000000	0.6412197126	-2.3931628477
H	1.0	0.0000000000	-2.3924771306	0.6406065083
N	7.0	0.0000000000	-2.0360500370	-2.0367154233
O	8.0	0.0000000000	-3.1789900469	-1.6635505533
O	8.0	0.0000000000	-1.6628707344	-3.1796770740
N	7.0	0.0000000000	-0.7450079396	2.7810243820
O	8.0	0.0000000000	-1.9213859878	3.0295026909
O	8.0	0.0000000000	0.1497040883	3.5842012139
N	7.0	0.0000000000	2.7817202895	-0.7456831275
O	8.0	0.0000000000	3.5848931732	0.1490246183
O	8.0	0.0000000000	3.0302089102	-1.9220760901
CL	17.0	3.0809432161	-0.0036399069	0.0083883425

(5) $Cl^{-}\cdots C_6H_3(CN)_3$

C	6.0	0.0000000000	1.2612899838	0.5852391134
C	6.0	0.0000000000	0.1222031209	1.3681498083
C	6.0	0.0000000000	-1.1376924291	0.7996868102
C	6.0	0.0000000000	-1.2461940505	-0.5782572490
C	6.0	0.0000000000	-0.1239336837	-1.3850768328
C	6.0	0.0000000000	1.1236502212	-0.7900879112

H	1.0	0.0000000000	2.2400264818	1.0394158934
H	1.0	0.0000000000	-0.2200105518	-2.4597582171
H	1.0	0.0000000000	-2.0203428573	1.4202787950
C	6.0	0.0000000000	2.3313892450	-1.6393474399
N	7.0	0.0000000000	3.2661687505	-2.2969119754
C	6.0	0.0000000000	0.2537121460	2.8387349686
N	7.0	0.0000000000	0.3555150706	3.9770799535
C	6.0	0.0000000000	-2.5854395791	-1.1997907115
N	7.0	0.0000000000	-3.6220362722	-1.6811187454
CL	17.0	3.0933859636	0.0015974038	0.0012737399

(6) C_6F_6

C	6.0	0.0000000000	0.0007314817	1.3774253597
C	6.0	0.0000000000	-1.1907115961	0.6878144960
C	6.0	0.0000000000	-1.1893295176	-0.6887707541
C	6.0	0.0000000000	0.0035266851	-1.3759247093
C	6.0	0.0000000000	1.1949900580	-0.6863403698
C	6.0	0.0000000000	1.1935787103	0.6902419998
F	9.0	0.0000000000	0.0051381892	-2.7357265908
F	9.0	0.0000000000	-2.3663031535	-1.3700378656
F	9.0	0.0000000000	-2.3693295073	1.3662517367
F	9.0	0.0000000000	-0.0008801811	2.7372107635
F	9.0	0.0000000000	2.3705899426	1.3714617835
F	9.0	0.0000000000	2.3736085192	-1.3647796479

(7) $C_6H_3(NO_2)_3$

C	6.0	0.0000000000	-1.0745599491	0.9005898996
C	6.0	0.0000000000	-1.2682191265	-0.4624353071
C	6.0	0.0000000000	-0.2426743056	-1.3809236308

C	6.0	0.0000000000	1.0345899308	-0.8670946745
C	6.0	0.0000000000	1.3173059507	0.4802762447
C	6.0	0.0000000000	0.2336758932	1.3294615586
H	1.0	0.0000000000	-1.9018245536	1.5939221850
H	1.0	0.0000000000	2.3313691741	0.8500681732
H	1.0	0.0000000000	-0.4294901876	-2.4440159151
N	7.0	0.0000000000	2.2136713744	-1.8554923466
O	8.0	0.0000000000	1.9313018812	-3.0218341130
O	8.0	0.0000000000	3.3128116596	-1.3738307824
N	7.0	0.0000000000	-2.7137286135	-0.9893807266
O	8.0	0.0000000000	-2.8461060511	-2.1820852384
O	8.0	0.0000000000	-3.5825731664	-0.1615797508
N	7.0	0.0000000000	0.4999888660	2.8447162956
O	8.0	0.0000000000	-0.4666776433	3.5558245578
O	8.0	0.0000000000	1.6511458667	3.1837395708

(8) $C_6H_3(CN)_3$

C	6.0	0.0000000000	1.2638054374	0.5864466653
C	6.0	0.0000000000	0.1226389779	1.3723996521
C	6.0	0.0000000000	-1.1398577962	0.8013054084
C	6.0	0.0000000000	-1.2499482660	-0.5799621057
C	6.0	0.0000000000	-0.1241118418	-1.3877252408
C	6.0	0.0000000000	1.1271483201	-0.7924428240
H	1.0	0.0000000000	2.2433694333	1.0409921114
H	1.0	0.0000000000	-0.2202608235	-2.4633116461
H	1.0	0.0000000000	-2.0232720818	1.4223909630
C	6.0	0.0000000000	2.3345139424	-1.6413320365
N	7.0	0.0000000000	3.2688957220	-2.2982393869

C	6.0	0.0000000000	0.2539438725	2.8424832260
N	7.0	0.0000000000	0.3564226306	3.9800598469
C	6.0	0.0000000000	-2.5887266088	-1.2012341753
N	7.0	0.0000000000	-3.6246579182	-1.6823204578

7. The xyz coordinates of complexes and monomers optimized at BLW(M062x-D3)/cc-pVTZ level of theory with the π electrons on fluorine atoms and C-C bonds both localized (in Å).

(1) $Cl^{-}\cdots C_6F_6$

C	6.0	0.0000000000	-0.0583214337	1.4042300891
C	6.0	0.0000000000	-1.1843431741	0.7526050190
C	6.0	0.0000000000	-1.1827650811	-0.7535177243
C	6.0	0.0000000000	-0.0554348591	-1.4028821165
C	6.0	0.0000000000	1.2481145379	-0.6484400697
C	6.0	0.0000000000	1.2468126217	0.6525335256
F	9.0	0.0000000000	0.0328587776	-2.7625257650
F	9.0	0.0000000000	-2.4030664791	-1.3595951666
F	9.0	0.0000000000	-2.4060790118	1.3558297426
F	9.0	0.0000000000	0.0267784659	2.7640557256
F	9.0	0.0000000000	2.3800798914	1.4089785493
F	9.0	0.0000000000	2.3831398318	-1.4022109321
CL	17.0	3.0859932664	0.0022259125	0.0009381229

(2) $Br^{-}\cdots C_6F_6$

C	6.0	0.0000000000	-0.0585581700	1.4045732087
C	6.0	0.0000000000	-1.1849440360	0.7527097094
C	6.0	0.0000000000	-1.1833571293	-0.7538124247
C	6.0	0.0000000000	-0.0556382093	-1.4033592461
C	6.0	0.0000000000	1.2482544275	-0.6487133414

C	6.0	0.0000000000	1.2469268514	0.6526754220
F	9.0	0.0000000000	0.0327252001	-2.7622731146
F	9.0	0.0000000000	-2.4031607402	-1.3593160596
F	9.0	0.0000000000	-2.4061234761	1.3554589857
F	9.0	0.0000000000	0.0266646371	2.7636785424
F	9.0	0.0000000000	2.3794683463	1.4089547092
F	9.0	0.0000000000	2.3825551856	-1.4023039395
BR	35.0	3.2743252257	0.0007581131	0.0005415485

(3) $I^{-}\cdots C_6F_6$

C	6.0	0.0000000000	-0.0585461355	1.4048279914
C	6.0	0.0000000000	-1.1851616419	0.7527885026
C	6.0	0.0000000000	-1.1835594601	-0.7538879829
C	6.0	0.0000000000	-0.0555774161	-1.4035657165
C	6.0	0.0000000000	1.2484496803	-0.6488306283
C	6.0	0.0000000000	1.2470922531	0.6528728603
F	9.0	0.0000000000	0.0330584563	-2.7619869788
F	9.0	0.0000000000	-2.4029965548	-1.3590120894
F	9.0	0.0000000000	-2.4059670104	1.3551671743
F	9.0	0.0000000000	0.0270194592	2.7634402440
F	9.0	0.0000000000	2.3791544223	1.4089712749
F	9.0	0.0000000000	2.3822154779	-1.4023543463
I	53.0	3.4852633728	0.0003894694	0.0003836949

(4) $Cl^{-}\cdots C_6H_3(NO_2)_3$

C	6.0	0.0000000000	0.9771683785	1.0470477874
C	6.0	0.0000000000	-0.2936162181	1.3438125020
C	6.0	0.0000000000	-1.3952761031	0.3227917705
C	6.0	0.0000000000	-1.0168214756	-0.9260851007

C	6.0	0.0000000000	0.4181621415	-1.3696367105
C	6.0	0.0000000000	1.3104894496	-0.4174525859
H	1.0	0.0000000000	1.7599812682	1.7860050928
H	1.0	0.0000000000	0.6668474309	-2.4170279670
H	1.0	0.0000000000	-2.4266933463	0.6310600499
N	7.0	0.0000000000	-2.0691578259	-2.0378934747
O	8.0	0.0000000000	-3.2218376529	-1.6990936554
O	8.0	0.0000000000	-1.6612317515	-3.1703082952
N	7.0	0.0000000000	-0.7302523434	2.8110891637
O	8.0	0.0000000000	-1.9149005669	3.0240758261
O	8.0	0.0000000000	0.1396375389	3.6398135101
N	7.0	0.0000000000	2.7995306661	-0.7730935612
O	8.0	0.0000000000	3.5763450984	0.1463368258
O	8.0	0.0000000000	3.0821564951	-1.9408116986
CL	17.0	3.1092074659	-0.0005311837	-0.0006294795

(5) $Cl^{-}\cdots C_6H_3(CN)_3$

C	6.0	0.0000000000	1.2734283546	0.6365660298
C	6.0	0.0000000000	0.1997372270	1.3906408261
C	6.0	0.0000000000	-1.1883039679	0.7845970701
C	6.0	0.0000000000	-1.3043140342	-0.5223045350
C	6.0	0.0000000000	-0.0854950446	-1.4212537683
C	6.0	0.0000000000	1.1044239424	-0.8684815779
H	1.0	0.0000000000	2.2719919320	1.0435834393
H	1.0	0.0000000000	-0.2327370668	-2.4894853040
H	1.0	0.0000000000	-2.0400340173	1.4459823773
C	6.0	0.0000000000	2.3369438260	-1.6752821223
N	7.0	0.0000000000	3.3063151027	-2.2810444649

C	6.0	0.0000000000	0.2828822768	2.8614605566
N	7.0	0.0000000000	0.3238964348	4.0038051925
C	6.0	0.0000000000	-2.6190947228	-1.1865631484
N	7.0	0.0000000000	-3.6281997954	-1.7235356548
CL	17.0	3.1216324493	-0.0015374474	0.0008250840

(6) C_6F_6

C	6.0	0.0000000000	-0.0592348867	1.4085427013
C	6.0	0.0000000000	-1.1881558373	0.7549486675
C	6.0	0.0000000000	-1.1864369278	-0.7563309639
C	6.0	0.0000000000	-0.0559567116	-1.4072245468
C	6.0	0.0000000000	1.2519856975	-0.6500847884
C	6.0	0.0000000000	1.2504210908	0.6543749631
F	9.0	0.0000000000	0.0342699344	-2.7611225606
F	9.0	0.0000000000	-2.4029205630	-1.3575013786
F	9.0	0.0000000000	-2.4057954866	1.3537674198
F	9.0	0.0000000000	0.0283791238	2.7626125137
F	9.0	0.0000000000	2.3778095929	1.4095306878
F	9.0	0.0000000000	2.3808165037	-1.4030824095

(7) $C_6H_3(NO_2)_3$

C	6.0	0.0000000000	-1.1304678306	0.8834059332
C	6.0	0.0000000000	-1.3181124658	-0.4113826696
C	6.0	0.0000000000	-0.1998128346	-1.4207969844
C	6.0	0.0000000000	1.0153193977	-0.9359148761
C	6.0	0.0000000000	1.3303204800	0.5372966249
C	6.0	0.0000000000	0.3028085397	1.3471880223
H	1.0	0.0000000000	-1.9360665925	1.6005333135
H	1.0	0.0000000000	2.3541425092	0.8765317686

H	1.0	0.0000000000	-0.4180065394	-2.4770494610
N	7.0	0.0000000000	2.2185755310	-1.8882607795
O	8.0	0.0000000000	1.9722223939	-3.0615014597
O	8.0	0.0000000000	3.3036834652	-1.3723117390
N	7.0	0.0000000000	-2.7445230090	-0.9771623907
O	8.0	0.0000000000	-2.8404788439	-2.1748404447
O	8.0	0.0000000000	-3.6373326500	-0.1770963176
N	7.0	0.0000000000	0.5259530350	2.8654005291
O	8.0	0.0000000000	-0.4634076328	3.5472074968
O	8.0	0.0000000000	1.6651900469	3.2386794336

(8) $C_6H_3(CN)_3$

C	6.0	0.0000000000	1.2771678129	0.6360676176
C	6.0	0.0000000000	0.2028118992	1.3946614756
C	6.0	0.0000000000	-1.1895445270	0.7877855815
C	6.0	0.0000000000	-1.3093136164	-0.5219265318
C	6.0	0.0000000000	-0.0875867337	-1.4243786381
C	6.0	0.0000000000	1.1065212281	-0.8732002632
H	1.0	0.0000000000	2.2777002901	1.0409020840
H	1.0	0.0000000000	-0.2371174631	-2.4933219253
H	1.0	0.0000000000	-2.0405079738	1.4517384462
C	6.0	0.0000000000	2.3398290401	-1.6777709586
N	7.0	0.0000000000	3.3120648348	-2.2775602392
C	6.0	0.0000000000	0.2832771670	2.8650482903
N	7.0	0.0000000000	0.3168126282	4.0069357695
C	6.0	0.0000000000	-2.6230696049	-1.1871653760
N	7.0	0.0000000000	-3.6291419812	-1.7283053325

8. The xyz coordinates of complexes and monomers optimized at B3LYP-D3/cc-pVTZ level of theory (in Å).

(1) $Cl^{-}\cdots C_6F_6$

C	6.0	0.0006844541	1.3840462753	-0.0271396464
C	6.0	-1.1969874647	0.6907658769	-0.0264930921
C	6.0	-1.1955388568	-0.6930909636	-0.0272146978
C	6.0	0.0036945896	-1.3837075750	-0.0289024501
C	6.0	1.2013936663	-0.6904604654	-0.0293862285
C	6.0	1.1998929242	0.6933954524	-0.0282813471
F	9.0	0.0051321480	-2.7221970138	-0.0077766823
F	9.0	-2.3538943574	-1.3636027082	-0.0039023345
F	9.0	-2.3567596906	1.3586742199	-0.0021986321
F	9.0	-0.0007094126	2.7223845888	-0.0042141505
F	9.0	2.3582575762	1.3638264274	-0.0058651061
F	9.0	2.3612635158	-1.3584607687	-0.0082550934
CL	17.0	0.0035709073	0.0084256541	3.1496274612

(2) $Br^{-}\cdots C_6F_6$

C	6.0	0.0005214459	1.3859688977	-0.0429447380
C	6.0	-1.1976087833	0.6924766814	-0.0413858674
C	6.0	-1.1961158142	-0.6918792856	-0.0391536066
C	6.0	0.0035227968	-1.3827235414	-0.0393203862
C	6.0	1.2016469222	-0.6892713839	-0.0407855380
C	6.0	1.2001592598	0.6950850688	-0.0424516774
F	9.0	0.0049758071	-2.7201606237	-0.0165437707
F	9.0	-2.3537401422	-1.3618170295	-0.0164920694
F	9.0	-2.3567240096	1.3600504047	-0.0211915669
F	9.0	-0.0009201204	2.7236319991	-0.0244015867

F	9.0	2.3578402076	1.3651632468	-0.0230126132
F	9.0	2.3607204702	-1.3566931784	-0.0200310123
BR	35.0	0.0057219599	-0.0098322560	3.3177124328

(3) $I^{-}\cdots C_6F_6$

C	6.0	0.0012526050	1.3859032029	-0.0593241790
C	6.0	-1.1971439894	0.6922685122	-0.0565427373
C	6.0	-1.1956329975	-0.6923889697	-0.0553909217
C	6.0	0.0042502051	-1.3834323971	-0.0567835473
C	6.0	1.2026386871	-0.6897952677	-0.0593890772
C	6.0	1.2011639230	0.6948753769	-0.0605746146
F	9.0	0.0057647532	-2.7205139750	-0.0360554868
F	9.0	-2.3528316943	-1.3621526802	-0.0328929247
F	9.0	-2.3558204264	1.3595839233	-0.0355452952
F	9.0	-0.0001473039	2.7231262293	-0.0412899691
F	9.0	2.3585498133	1.3647506010	-0.0437179808
F	9.0	2.3614667664	-1.3570636577	-0.0414447963
I	53.0	-0.0035103416	-0.0051618981	3.5289495302

(4) $Cl^{-}\cdots C_6H_3(NO_2)_3$

C	6.0	1.0674111403	0.9721286239	-0.4302282718
C	6.0	-0.2560066326	1.3070086987	-0.2842974801
C	6.0	-1.2675683724	0.3391515457	-0.0500113635
C	6.0	-0.8745142374	-1.0046109997	-0.2839068351
C	6.0	0.4393238361	-1.3754197855	-0.4300644100
C	6.0	1.4148290541	-0.3786211240	-0.4697488779
H	1.0	1.8194254294	1.7359463771	-0.5333574686
H	1.0	0.7093943027	-2.4127445228	-0.5330974924
H	1.0	-2.3007206432	0.6155245455	-0.1371996480

N	7.0	-1.9049900986	-2.0330199199	-0.3195149666
O	8.0	-3.0748043464	-1.6690940983	-0.3228304792
O	8.0	-1.5529616817	-3.2105459282	-0.3655542903
N	7.0	-0.6352623141	2.7126366430	-0.3207986794
O	8.0	-1.8304723184	2.9816164435	-0.3243999907
O	8.0	0.2577904116	3.5569418072	-0.3672951644
N	7.0	2.8046549262	-0.7503811408	-0.6362844898
O	8.0	3.6440305672	0.1472370555	-0.7036521374
O	8.0	3.0838656084	-1.9471418885	-0.7043137396
CL	17.0	-1.5434246314	0.4133876674	2.3061557847

(5) $Cl^{-}\cdots C_6H_3(CN)_3$

C	6.0	1.0818961979	0.9637201524	-0.6073907010
C	6.0	1.4492065407	-0.3862923575	-0.6734652919
C	6.0	0.4589230193	-1.3745056584	-0.6066001504
C	6.0	-0.8707289638	-1.0196086010	-0.4311198658
C	6.0	-1.2391153017	0.3301085700	-0.2506327759
C	6.0	-0.2480321274	1.3175219419	-0.4319604957
H	1.0	1.8358733708	1.7317572189	-0.7020822626
H	1.0	-2.2755444476	0.6062573818	-0.1899499338
H	1.0	0.7307776121	-2.4159123520	-0.7007671896
C	6.0	-0.6061696103	2.6982386314	-0.4177019352
N	7.0	-0.8727938836	3.8196580360	-0.4577581073
C	6.0	2.8131940038	-0.7497364642	-0.8584608051
N	7.0	3.9186870150	-1.0444868320	-1.0165501310
C	6.0	-1.8682861805	-2.0391548428	-0.4158885098
N	7.0	-2.6575739122	-2.8792235125	-0.4550324439
CL	17.0	-1.6503133331	0.4416586876	2.3464132389

(6) C_6F_6

C	6.0	0.0004144545	1.3897991577	-0.0125679445
C	6.0	-1.2017494831	0.6939062171	-0.0114973301
C	6.0	-1.2002393699	-0.6951254638	-0.0117293172
C	6.0	0.0034458296	-1.3883351035	-0.0124460523
C	6.0	1.2056180006	-0.6925027508	-0.0123278431
C	6.0	1.2041168414	0.6965477080	-0.0131400709
F	9.0	0.0048715798	-2.7202782130	-0.0130526014
F	9.0	-2.3529985622	-1.3623682554	-0.0112643374
F	9.0	-2.3559686671	1.3586326666	-0.0104054602
F	9.0	-0.0010235556	2.7217483605	-0.0129250955
F	9.0	2.3568928184	1.3637794097	-0.0143474224
F	9.0	2.3598264146	-1.3572539471	-0.0115609944

(7) $C_6H_3(NO_2)_3$

C	6.0	0.9914852083	0.9914835616	-0.4216000000
C	6.0	-0.3539105142	1.3207603179	-0.4216000000
C	6.0	-1.3544191560	0.3629057358	-0.4216000000
C	6.0	-0.9668863865	-0.9668867571	-0.4216000000
C	6.0	0.3629066064	-1.3544200253	-0.4216000000
C	6.0	1.3207571350	-0.3539129386	-0.4216000000
H	1.0	1.7538153679	1.7538107828	-0.4216000000
H	1.0	0.6419183656	-2.3957850358	-0.4216000000
H	1.0	-2.3957842780	0.6419153342	-0.4216000000
N	7.0	-2.0174598902	-2.0174593412	-0.4216000000
O	8.0	-3.1767547472	-1.6434329115	-0.4216000000
O	8.0	-1.6434347306	-3.1767531782	-0.4216000000
N	7.0	-0.7384054395	2.7558899930	-0.4216000000

O	8.0	-1.9293896483	3.0116703808	-0.4216000000
O	8.0	0.1651750081	3.5728304062	-0.4216000000
N	7.0	2.7558884284	-0.7384057046	-0.4216000000
O	8.0	3.5728265666	0.1651772978	-0.4216000000
O	8.0	3.0116721042	-1.9293879178	-0.4216000000

(8) $C_6H_3(CN)_3$

C	6.0	0.9888139477	0.9888074237	-0.5636842100
C	6.0	1.3455796727	-0.3605330118	-0.5636842100
C	6.0	0.3619421509	-1.3507091449	-0.5636842100
C	6.0	-0.9850095100	-0.9850304144	-0.5636842100
C	6.0	-1.3506966653	0.3619174277	-0.5636842100
C	6.0	-0.3605305422	1.3455626140	-0.5636842100
H	1.0	1.7525263816	1.7525249667	-0.5636842100
H	1.0	-2.3939504539	0.6414576987	-0.5636842100
H	1.0	0.6414878281	-2.3939618897	-0.5636842100
C	6.0	-0.7306859334	2.7269997279	-0.5636842100
N	7.0	-1.0288594848	3.8391858805	-0.5636842100
C	6.0	2.7270163821	-0.7306931903	-0.5636842100
N	7.0	3.8392235279	-1.0287922693	-0.5636842100
C	6.0	-1.9962607275	-1.9963294151	-0.5636842100
N	7.0	-2.8105965739	-2.8104064038	-0.5636842100

9. The xyz coordinates of all complexes and monomers optimized at CAMB3LYP-D3/cc-pVTZ level of theory (in Å).

(1) $Cl^{-}\cdots C_6F_6$

C	6.0	0.0012468907	1.3778669535	-0.0235286660
C	6.0	-1.1909818467	0.6877666142	-0.0221950959
C	6.0	-1.1895054735	-0.6897819830	-0.0226481313

C	6.0	0.0042348125	-1.3772851855	-0.0247408143
C	6.0	1.1964839798	-0.6871858774	-0.0262288941
C	6.0	1.1949989600	0.6903781833	-0.0254746768
F	9.0	0.0057025919	-2.7090823113	-0.0069593660
F	9.0	-2.3420474356	-1.3569042837	-0.0023468311
F	9.0	-2.3449273199	1.3523391573	-0.0012366377
F	9.0	-0.0001440046	2.7095556327	-0.0043781587
F	9.0	2.3476652800	1.3575020596	-0.0087590510
F	9.0	2.3506242647	-1.3518552361	-0.0103350459
CL	17.0	-0.0033506996	0.0066852765	3.1288293689

(2) $Br^{-}\cdots C_6F_6$

C	6.0	0.0007577748	1.3795088656	-0.0389474962
C	6.0	-1.1919062026	0.6891675425	-0.0371210760
C	6.0	-1.1903969554	-0.6888683587	-0.0355935724
C	6.0	0.0037533479	-1.3765908014	-0.0358873098
C	6.0	1.1964145095	-0.6862795004	-0.0377513625
C	6.0	1.1949160614	0.6917554984	-0.0392555827
F	9.0	0.0052225775	-2.7074274239	-0.0176442657
F	9.0	-2.3422599026	-1.3554948433	-0.0170694472
F	9.0	-2.3452377308	1.3534356620	-0.0203762615
F	9.0	-0.0006713959	2.7105294988	-0.0242930537
F	9.0	2.3468604366	1.3585118480	-0.0246382614
F	9.0	2.3497684129	-1.3504359501	-0.0214852417
BR	35.0	0.0027790665	-0.0078130376	3.3000609309

(3) $I^{-}\cdots C_6F_6$

C	6.0	0.0004960027	1.3792080528	-0.0547352397
C	6.0	-1.1924722303	0.6886483408	-0.0535658659

C	6.0	-1.1909056584	-0.6897780204	-0.0534968282
C	6.0	0.0036126590	-1.3776494576	-0.0542411139
C	6.0	1.1965722627	-0.6870793270	-0.0550707655
C	6.0	1.1950224162	0.6913440364	-0.0553429979
F	9.0	0.0049782674	-2.7079945197	-0.0395485253
F	9.0	-2.3423448244	-1.3561045707	-0.0379478740
F	9.0	-2.3452697165	1.3526435485	-0.0385261365
F	9.0	-0.0008424138	2.7095693709	-0.0408222390
F	9.0	2.3464814496	1.3576687627	-0.0415942527
F	9.0	2.3493796064	-1.3510614780	-0.0408882539
I	53.0	0.0052921797	0.0005842613	3.5157780925

(4) $Cl^{-}\cdots C_6H_3(NO_2)_3$

C	6.0	0.9846605029	0.9852785079	-0.4202649810
C	6.0	-0.3519093823	1.3126724027	-0.4343039430
C	6.0	-1.3456906624	0.3608753629	-0.4210580341
C	6.0	-0.9609368723	-0.9602513578	-0.4341614084
C	6.0	0.3602427977	-1.3450163308	-0.4191416230
C	6.0	1.3120132809	-0.3512274282	-0.4336841699
H	1.0	1.7448213446	1.7454153714	-0.3834861791
H	1.0	0.6384473007	-2.3833455271	-0.3813422094
H	1.0	-2.3841049802	0.6390881516	-0.3850869460
N	7.0	-1.9972981750	-1.9967086093	-0.4221147572
O	8.0	-3.1558229398	-1.6362664378	-0.4297287002
O	8.0	-1.6369414388	-3.1552166164	-0.4257153404
N	7.0	-0.7312132667	2.7284627445	-0.4222885242
O	8.0	-1.9147430829	2.9955733728	-0.4265151352
O	8.0	0.1601699431	3.5515608789	-0.4289786418

N	7.0	2.7277662563	-0.7305940828	-0.4212714800
O	8.0	3.5508515262	0.1608040321	-0.4299918968
O	8.0	2.9949206288	-1.9140756200	-0.4236349706
CL	17.0	0.0047672191	-0.0070288147	2.6323689404

(5) $Cl^{-}\cdots C_6H_3(CN)_3$

C	6.0	0.9815177924	0.9814811742	-0.5516545133
C	6.0	1.3348067456	-0.3575570489	-0.5593361433
C	6.0	0.3592959820	-1.3405357169	-0.5513106372
C	6.0	-0.9769887753	-0.9769750084	-0.5591157581
C	6.0	-1.3405122726	0.3593172726	-0.5512974892
C	6.0	-0.3575205383	1.3348048638	-0.5593306214
H	1.0	1.7438718630	1.7438307464	-0.5215914197
H	1.0	-2.3818819865	0.6383851010	-0.5208247920
H	1.0	0.6383417901	-2.3819119848	-0.5208669036
C	6.0	-0.7274701834	2.7157696939	-0.5650426266
N	7.0	-1.0241270040	3.8215128148	-0.6105569373
C	6.0	2.7157536754	-0.7275646768	-0.5649259227
N	7.0	3.8216347932	-1.0237588860	-0.6103065214
C	6.0	-1.9878834089	-1.9879100677	-0.5645365382
N	7.0	-2.7976016304	-2.7972673111	-0.6098671688
CL	17.0	-0.0012368423	-0.0016209663	2.5516166329

(6) C_6F_6

C	6.0	0.0004158739	1.3831748380	-0.0123893881
C	6.0	-1.1960047054	0.6905959158	-0.0115579436
C	6.0	-1.1945235012	-0.6918283933	-0.0117117295
C	6.0	0.0034771824	-1.3816945220	-0.0122050881
C	6.0	1.1999324076	-0.6891657900	-0.0122929530

C	6.0	1.1983690398	0.6932493980	-0.0131124561
F	9.0	0.0048381675	-2.7071897934	-0.0127610256
F	9.0	-2.3417279765	-1.3557956535	-0.0114177761
F	9.0	-2.3446530379	1.3520808066	-0.0108111728
F	9.0	-0.0010142353	2.7086643272	-0.0125818557
F	9.0	2.3455824494	1.3571873484	-0.0145573953
F	9.0	2.3485146367	-1.3507286958	-0.0118656853

(7) $C_6H_3(NO_2)_3$

C	6.0	0.9873500353	0.9873487326	-0.4216000000
C	6.0	-0.3520215200	1.3137267902	-0.4216000000
C	6.0	-1.3487630731	0.3613926646	-0.4216000000
C	6.0	-0.9617297319	-0.9617302871	-0.4216000000
C	6.0	0.3613936386	-1.3487642942	-0.4216000000
C	6.0	1.3137230542	-0.3520237094	-0.4216000000
H	1.0	1.7493417928	1.7493376146	-0.4216000000
H	1.0	0.6402905950	-2.3896653278	-0.4216000000
H	1.0	-2.3896649767	0.6402877081	-0.4216000000
N	7.0	-2.0064667572	-2.0064690564	-0.4216000000
O	8.0	-3.1574911901	-1.6350649661	-0.4216000000
O	8.0	-1.6350687590	-3.1574905206	-0.4216000000
N	7.0	-0.7343917658	2.7408768461	-0.4216000000
O	8.0	-1.9169031349	2.9947826437	-0.4216000000
O	8.0	0.1627704449	3.5519769163	-0.4216000000
N	7.0	2.7408739713	-0.7343935277	-0.4216000000
O	8.0	3.5519722511	0.1627727074	-0.4216000000
O	8.0	2.9947851256	-1.9169009342	-0.4216000000

(8) $C_6H_3(CN)_3$

C	6.0	0.9838754232	0.9838776064	-0.5636842100
C	6.0	1.3378344087	-0.3584673149	-0.5636842100
C	6.0	0.3601241674	-1.3439895759	-0.5636842100
C	6.0	-0.9793625264	-0.9793648919	-0.5636842100
C	6.0	-1.3439916608	0.3601218937	-0.5636842100
C	6.0	-0.3584713172	1.3378328829	-0.5636842100
H	1.0	1.7471531879	1.7471582380	-0.5636842100
H	1.0	-2.3866510241	0.6395019933	-0.5636842100
H	1.0	0.6395016608	-2.3866494209	-0.5636842100
C	6.0	-0.7288915757	2.7202001252	-0.5636842100
N	7.0	-1.0251340986	3.8258363423	-0.5636842100
C	6.0	2.7202036859	-0.7288823378	-0.5636842100
N	7.0	3.8258368500	-1.0251487833	-0.5636842100
C	6.0	-1.9913271184	-1.9913198461	-0.5636842100
N	7.0	-2.8007000628	-2.8007069107	-0.5636842100

10. The xyz coordinates of complexes and monomers optimized at HF/cc-pVTZ level of theory (in Å).

(1) $Cl^{-}\cdots C_6F_6$

C	6.0	0.0007289826	1.3732645171	-0.0357448088
C	6.0	-1.1868951243	0.6859497118	-0.0341025447
C	6.0	-1.1853849143	-0.6862165357	-0.0330986834
C	6.0	0.0036455451	-1.3711194489	-0.0336149644
C	6.0	1.1912099248	-0.6836917337	-0.0351482229
C	6.0	1.1898252881	0.6884799892	-0.0362580894
F	9.0	0.0051299541	-2.6833006436	-0.0308779176
F	9.0	-2.3210119341	-1.3436512630	-0.0303493675
F	9.0	-2.3240542588	1.3407765153	-0.0323977459

F	9.0	-0.0007058960	2.6855078408	-0.0350900445
F	9.0	2.3255235048	1.3458512637	-0.0360414974
F	9.0	2.3283389960	-1.3385488801	-0.0340433665
CL	17.0	0.0036499318	-0.0033023328	3.3567652530

(2) $Br^{-}\cdots C_6F_6$

C	6.0	0.0007191105	1.3740350675	-0.0531077954
C	6.0	-1.1873762709	0.6864263269	-0.0516281650
C	6.0	-1.1858196734	-0.6862922356	-0.0503850195
C	6.0	0.0037057545	-1.3713910290	-0.0505480825
C	6.0	1.1917754350	-0.6837314057	-0.0517781192
C	6.0	1.1902759402	0.6889910006	-0.0530651342
F	9.0	0.0051730531	-2.6827228132	-0.0509237052
F	9.0	-2.3207735676	-1.3432003612	-0.0505553385
F	9.0	-2.3237771652	1.3408861839	-0.0530749647
F	9.0	-0.0007238650	2.6854447062	-0.0561492296
F	9.0	2.3252890502	1.3458689057	-0.0558853850
F	9.0	2.3281328330	-1.3381996638	-0.0531728180
BR	35.0	0.0033993655	-0.0061156821	3.5802717567

(3) $I^{-}\cdots C_6F_6$

C	6.0	0.0018247601	1.3721348807	-0.0248723891
C	6.0	-1.1866222267	0.6842954450	-0.0245214153
C	6.0	-1.1850670888	-0.6888306212	-0.0279287322
C	6.0	0.0048132457	-1.3741535228	-0.0318872508
C	6.0	1.1932332222	-0.6862516855	-0.0325410010
C	6.0	1.1917464991	0.6868896658	-0.0288280072
F	9.0	0.0062986973	-2.6849678067	-0.0394459065
F	9.0	-2.3194529703	-1.3454386876	-0.0315311113

F	9.0	-2.3223329764	1.3383941855	-0.0247404421
F	9.0	0.0004511285	2.6827541737	-0.0251540672
F	9.0	2.3262207927	1.3434011262	-0.0332354352
F	9.0	2.3291488474	-1.3404789724	-0.0409242359
I	53.0	-0.0146909312	0.0210658192	3.8886919833

(4) $Cl^{-} \cdots C_6H_3(NO_2)_3$

C	6.0	0.9832179259	0.9834809964	-0.4209182383
C	6.0	-0.3512141965	1.3113074569	-0.4354237396
C	6.0	-1.3429414064	0.3601703128	-0.4209423732
C	6.0	-0.9596275477	-0.9593536583	-0.4346243331
C	6.0	0.3599160485	-1.3427274326	-0.4199297604
C	6.0	1.3109841789	-0.3509325507	-0.4348230949
H	1.0	1.7367779023	1.7369988551	-0.3924624308
H	1.0	0.6357173891	-2.3720461880	-0.3905598694
H	1.0	-2.3722887813	0.6359468593	-0.3926128324
N	7.0	-1.9908642305	-1.9907323658	-0.4335379159
O	8.0	-3.1222103181	-1.6363360137	-0.4447837217
O	8.0	-1.6365929958	-3.1220690354	-0.4469249575
N	7.0	-0.7286849416	2.7201183590	-0.4349595437
O	8.0	-1.8856323646	2.9788844968	-0.4468742138
O	8.0	0.1438964876	3.5226838639	-0.4478058273
N	7.0	2.7197411287	-0.7284891731	-0.4341108710
O	8.0	3.5223960604	0.1440779679	-0.4470286001
O	8.0	2.9786956768	-1.8853992298	-0.4463435083
CL	17.0	-0.0012860194	-0.0055835242	2.8142658313

(5) $Cl^{-} \cdots C_6H_3(CN)_3$

C	6.0	0.9781640026	0.9780715698	-0.5584152680

C	6.0	1.3303203578	-0.3571114313	-0.5652828823
C	6.0	0.3577906935	-1.3373440095	-0.5569949956
C	6.0	-0.9745595151	-0.9746410884	-0.5658843631
C	6.0	-1.3372527398	0.3577161980	-0.5579654232
C	6.0	-0.3570198618	1.3302458083	-0.5655802652
H	1.0	1.7349898817	1.7349659747	-0.5373771301
H	1.0	-2.3711637134	0.6348262621	-0.5360629534
H	1.0	0.6349595066	-2.3712230160	-0.5340864073
C	6.0	-0.7297080211	2.7214451148	-0.5787872435
N	7.0	-1.0213051040	3.8101325937	-0.6310756472
C	6.0	2.7215161998	-0.7297803106	-0.5793437422
N	7.0	3.8101665887	-1.0214038835	-0.6330763518
C	6.0	-1.9928972623	-1.9931328304	-0.5828908839
N	7.0	-2.7899317901	-2.7898618426	-0.6385810978
CL	17.0	0.0059307768	0.0070948908	2.7524572947

(6) C_6F_6

C	6.0	0.0008647391	1.3761048345	-0.0097438880
C	6.0	-1.1899862286	0.6868460605	-0.0090814626
C	6.0	-1.1884960704	-0.6890831803	-0.0099028993
C	6.0	0.0038292877	-1.3757805782	-0.0115513515
C	6.0	1.1946797630	-0.6865187566	-0.0123165399
C	6.0	1.1931961164	0.6894138564	-0.0115960622
F	9.0	0.0052629691	-2.6824814883	-0.0124703403
F	9.0	-2.3194171707	-1.3436659550	-0.0090512744
F	9.0	-2.3223156577	1.3389880832	-0.0076898256
F	9.0	-0.0005302461	2.6828023352	-0.0085785154
F	9.0	2.3241269652	1.3439802497	-0.0127099865

F	9.0	2.3270198753	-1.3386456465	-0.0136969983

(7) $C_6H_3(NO_2)_3$

C	6.0	0.9854013772	0.9854013834	-0.4216000000
C	6.0	-0.3517803632	1.3128624185	-0.4216000000
C	6.0	-1.3460848486	0.3606836550	-0.4216000000
C	6.0	-0.9610780197	-0.9610780151	-0.4216000000
C	6.0	0.3606836522	-1.3460848485	-0.4216000000
C	6.0	1.3128624117	-0.3517803560	-0.4216000000
H	1.0	1.7403072547	1.7403072570	-0.4216000000
H	1.0	0.6369912242	-2.3773075059	-0.4216000000
H	1.0	-2.3773075067	0.6369912266	-0.4216000000
N	7.0	-1.9968104079	-1.9968104263	-0.4216000000
O	8.0	-3.1222252131	-1.6333066895	-0.4216000000
O	8.0	-1.6333066865	-3.1222252320	-0.4216000000
N	7.0	-0.7308844944	2.7277034726	-0.4216000000
O	8.0	-1.8872773219	2.9755922014	-0.4216000000
O	8.0	0.1466406774	3.5205726216	-0.4216000000
N	7.0	2.7277034619	-0.7308845069	-0.4216000000
O	8.0	3.5205726044	0.1466406743	-0.4216000000
O	8.0	2.9755921982	-1.8872773302	-0.4216000000

(8) $C_6H_3(CN)_3$

C	6.0	0.9808408165	0.9808408132	-0.5636842100
C	6.0	1.3333439022	-0.3572658757	-0.5636842100
C	6.0	0.3590152397	-1.3398503366	-0.5636842100
C	6.0	-0.9760694519	-0.9760694478	-0.5636842100
C	6.0	-1.3398503356	0.3590152456	-0.5636842100
C	6.0	-0.3572658711	1.3333439040	-0.5636842100

H	1.0	1.7383308642	1.7383308577	-0.5636842100
H	1.0	-2.3746012972	0.6362714148	-0.5636842100
H	1.0	0.6362714047	-2.3746012993	-0.5636842100
C	6.0	-0.7302139566	2.7251855714	-0.5636842100
N	7.0	-1.0219265948	3.8138008096	-0.5636842100
C	6.0	2.7251855664	-0.7302139727	-0.5636842100
N	7.0	3.8138008139	-1.0219265764	-0.5636842100
C	6.0	-1.9949676609	-1.9949676469	-0.5636842100
N	7.0	-2.7918934394	-2.7918934609	-0.5636842100

11. The xyz coordinates of all complexes and monomers optimized at ωB97X/cc-pVTZ level of theory (in Å).

(1) $Cl^{-}\cdots C_6F_6$

C	6.0	0.0007560402	1.3811027963	-0.0242224679
C	6.0	-1.1939038346	0.6896470665	-0.0229026048
C	6.0	-1.1924365506	-0.6906953957	-0.0218123898
C	6.0	0.0037364517	-1.3795465038	-0.0234630484
C	6.0	1.1984073130	-0.6880849229	-0.0245110082
C	6.0	1.1969429469	0.6922689211	-0.0241755036
F	9.0	0.0052011672	-2.7125037745	-0.0119789340
F	9.0	-2.3460753988	-1.3584108747	-0.0081274589
F	9.0	-2.3489936577	1.3548897631	-0.0101125728
F	9.0	-0.0006736803	2.7140873811	-0.0135395916
F	9.0	2.3506121257	1.3600173979	-0.0128227233
F	9.0	2.3535414819	-1.3532972944	-0.0140636583
CL	17.0	0.0028855952	0.0005244402	3.1617299616

(2) $Br^{-}\cdots C_6F_6$

C	6.0	0.0013632089	1.3819670389	-0.0387701384

C	6.0	-1.1937732713	0.6902204330	-0.0362948090
C	6.0	-1.1921600077	-0.6906595879	-0.0353353594
C	6.0	0.0044427275	-1.3798211283	-0.0369759037
C	6.0	1.1995478118	-0.6880262677	-0.0395772863
C	6.0	1.1980246103	0.6928715538	-0.0404077914
F	9.0	0.0059139844	-2.7119128153	-0.0269764020
F	9.0	-2.3450275840	-1.3578770553	-0.0234261042
F	9.0	-2.3480332793	1.3551068753	-0.0250423279
F	9.0	-0.0000011391	2.7141327924	-0.0304859938
F	9.0	2.3510686479	1.3601698242	-0.0337941382
F	9.0	2.3539641005	-1.3528918263	-0.0321050959
BR	35.0	-0.0053298101	-0.0032808369	3.3491893501

(3) $I^{-}\cdots C_6F_6$

C	6.0	0.0008922635	1.3814689194	-0.0102016631
C	6.0	-1.1945456543	0.6895354219	-0.0102441049
C	6.0	-1.1930530570	-0.6917231870	-0.0112659702
C	6.0	0.0038982532	-1.3810540523	-0.0122918008
C	6.0	1.1993521065	-0.6891226293	-0.0122316396
C	6.0	1.1978395554	0.6921414494	-0.0111733086
F	9.0	0.0053577525	-2.7126269734	-0.0066068419
F	9.0	-2.3454319004	-1.3587622605	-0.0042426858
F	9.0	-2.3483589357	1.3539929640	-0.0019816335
F	9.0	-0.0005327269	2.7129413714	-0.0019299439
F	9.0	2.3502697787	1.3591229867	-0.0041106265
F	9.0	2.3532422437	-1.3536718735	-0.0064301765
I	53.0	-0.0033586792	0.0065718631	3.6157923846

(4) $Cl^{-}\cdots C_6H_3(NO_2)_3$

C	6.0	0.9883233579	0.9881152076	-0.4260622047
C	6.0	-0.3505744347	1.3142819309	-0.4355952771
C	6.0	-1.3470044946	0.3622415481	-0.4219389848
C	6.0	-0.9601065229	-0.9603415044	-0.4332055403
C	6.0	0.3624649128	-1.3472441285	-0.4221387314
C	6.0	1.3144915311	-0.3507965102	-0.4360643889
H	1.0	1.7502630693	1.7500247162	-0.3929606393
H	1.0	0.6412800751	-2.3879078522	-0.3849017732
H	1.0	-2.3876929285	0.6410438688	-0.3844702031
N	7.0	-1.9997693698	-2.0000502171	-0.4177314768
O	8.0	-3.1554484711	-1.6387179926	-0.4273735386
O	8.0	-1.6384446610	-3.1557397593	-0.4184089178
N	7.0	-0.7310761924	2.7345856051	-0.4231672353
O	8.0	-1.9125152498	2.9997256327	-0.4300817990
O	8.0	0.1597010978	3.5548609367	-0.4282851833
N	7.0	2.7347762729	-0.7313119596	-0.4249102987
O	8.0	3.5551347798	0.1593923658	-0.4303433187
O	8.0	3.0000214349	-1.9127490070	-0.4325245733
CL	17.0	-0.0238242067	-0.0194128810	2.6597640843

(5) $Cl^{-}\cdots C_6H_3(CN)_3$

C	6.0	0.9841108225	0.9830937938	-0.5524593127
C	6.0	1.3367254337	-0.3580810767	-0.5596697345
C	6.0	0.3607301076	-1.3432387008	-0.5520528166
C	6.0	-0.9770261871	-0.9780648408	-0.5579682953
C	6.0	-1.3421480025	0.3597244118	-0.5500734284
C	6.0	-0.3570195789	1.3357065496	-0.5582063835
H	1.0	1.7479444712	1.7469211568	-0.5250989021

H	1.0	-2.3854737287	0.6392996031	-0.5202574546
H	1.0	0.6402875718	-2.3866392255	-0.5242398673
C	6.0	-0.7286529872	2.7228786573	-0.5653878296
N	7.0	-1.0253859197	3.8309505794	-0.6141549924
C	6.0	2.7238519781	-0.7298196068	-0.5687130402
N	7.0	3.8319435567	-1.0265114490	-0.6185546580
C	6.0	-1.9924268886	-1.9935804517	-0.5649590560
N	7.0	-2.8034855737	-2.8048286694	-0.6137827229
CL	17.0	-0.0139750754	0.0021892689	2.5766311341

(6) C_6F_6

C	6.0	0.0008804755	1.3848136993	-0.0096309703
C	6.0	-1.1974946494	0.6911971248	-0.0092033188
C	6.0	-1.1960290260	-0.6934444398	-0.0098219997
C	6.0	0.0038510675	-1.3844327673	-0.0115240617
C	6.0	1.2022254477	-0.6908572785	-0.0124297592
C	6.0	1.2007346118	0.6937689368	-0.0115143434
F	9.0	0.0052910901	-2.7111957075	-0.0122512242
F	9.0	-2.3442933887	-1.3581254225	-0.0088750494
F	9.0	-2.3472689778	1.3532466444	-0.0080880178
F	9.0	-0.0006169741	2.7115687948	-0.0084036611
F	9.0	2.3490018975	1.3584087623	-0.0123976556
F	9.0	2.3519527683	-1.3529885321	-0.0142490829

(7) $C_6H_3(NO_2)_3$

C	6.0	0.9891576002	0.9891564347	-0.4216000000
C	6.0	-0.3523744474	1.3150549551	-0.4216000000
C	6.0	-1.3512341387	0.3620494673	-0.4216000000
C	6.0	-0.9627230622	-0.9627229704	-0.4216000000

C	6.0	0.3620499561	-1.3512353721	-0.4216000000
C	6.0	1.3150508477	-0.3523766960	-0.4216000000
H	1.0	1.7526873981	1.7526832910	-0.4216000000
H	1.0	0.6415458884	-2.3942290357	-0.4216000000
H	1.0	-2.3942282548	0.6415438481	-0.4216000000
N	7.0	-2.0091928826	-2.0091954401	-0.4216000000
O	8.0	-3.1581446494	-1.6375100894	-0.4216000000
O	8.0	-1.6375123971	-3.1581461207	-0.4216000000
N	7.0	-0.7353836591	2.7445881044	-0.4216000000
O	8.0	-1.9162388712	2.9972176435	-0.4216000000
O	8.0	0.1609919800	3.5537508676	-0.4216000000
N	7.0	2.7445847627	-0.7353852991	-0.4216000000
O	8.0	3.5537447634	0.1609940652	-0.4216000000
O	8.0	2.9972191659	-1.9162376534	-0.4216000000

(8) $C_6H_3(CN)_3$

C	6.0	0.9854249076	0.9854234537	-0.5636842100
C	6.0	1.3387670998	-0.3587158777	-0.5636842100
C	6.0	0.3606906023	-1.3461040458	-0.5636842100
C	6.0	-0.9800394264	-0.9800410569	-0.5636842100
C	6.0	-1.3461036082	0.3606896404	-0.5636842100
C	6.0	-0.3587157150	1.3387646350	-0.5636842100
H	1.0	1.7500209220	1.7500199602	-0.5636842100
H	1.0	-2.3905611506	0.6405527140	-0.5636842100
H	1.0	0.6405499488	-2.3905623345	-0.5636842100
C	6.0	-0.7307304695	2.7270924105	-0.5636842100
N	7.0	-1.0276434498	3.8350778469	-0.5636842100
C	6.0	2.7270945933	-0.7307366206	-0.5636842100

N	7.0	3.8350788003	-1.0276337656	-0.5636842100
C	6.0	-1.9963629502	-1.9963615116	-0.5636842100
N	7.0	-2.8074701046	-2.8074654480	-0.5636842100

12. The xyz coordinates of complexes and monomers optimized at SCSMP2/cc-pVTZ level of theory (in Å).

(1) $Cl^{-}\cdots C_6F_6$

6	0	0.000564	1.386457	-0.020709
6	0	-1.198755	0.692286	-0.019769
6	0	-1.197232	-0.693453	-0.019535
6	0	0.003595	-1.385037	-0.020354
6	0	1.202919	-0.690875	-0.021290
6	0	1.201366	0.694852	-0.021456
9	0	0.005044	-2.721377	-0.006734
9	0	-2.353802	-1.362877	-0.005066
9	0	-2.356783	1.359192	-0.005581
9	0	-0.000857	2.722802	-0.007567
9	0	2.357950	1.364276	-0.008130
9	0	2.360971	-1.357771	-0.008706
17	0	0.005018	0.001524	3.114896

(2) $Br^{-}\cdots C_6F_6$

6	0	0.001438	1.387415	-0.034851
6	0	-1.198339	0.692999	-0.032486
6	0	-1.196871	-0.693241	-0.031509
6	0	0.004418	-1.385025	-0.033070
6	0	1.204183	-0.690590	-0.035434
6	0	1.202756	0.695661	-0.036531
9	0	0.006001	-2.720370	-0.016627

9	0	-2.352517	-1.362132	-0.013416
9	0	-2.355435	1.359525	-0.015482
9	0	-0.000030	2.722901	-0.021186
9	0	2.358605	1.364660	-0.024471
9	0	2.361466	-1.357098	-0.022331
35	0	-0.005674	-0.004708	3.267392

(3) $I^{-}\cdots C_6F_6$

6	0	0.000729	1.387452	-0.050457
6	0	-1.199399	0.692825	-0.050279
6	0	-1.197900	-0.693831	-0.050471
6	0	0.003734	-1.385851	-0.050863
6	0	1.203858	-0.691216	-0.051041
6	0	1.202369	0.695441	-0.050975
9	0	0.005192	-2.720628	-0.038244
9	0	-2.353121	-1.362475	-0.037584
9	0	-2.356068	1.358961	-0.037098
9	0	-0.000721	2.722225	-0.037342
9	0	2.357591	1.364078	-0.038365
9	0	2.360532	-1.357355	-0.038488
53	0	0.003203	0.000372	3.481203

(4) $Cl^{-}\cdots C_6H_3(NO_2)_3$

6	0	0.994442	0.992930	-0.432186
6	0	-0.351323	1.314316	-0.431647
6	0	-1.356029	0.363043	-0.430043
6	0	-0.961574	-0.963152	-0.432296
6	0	0.364599	-1.357719	-0.432793
6	0	1.315857	-0.352894	-0.433640

1	0	1.754382	1.752962	-0.392718
1	0	0.642738	-2.395919	-0.394004
1	0	-2.394110	0.641218	-0.388938
7	0	-2.003152	-2.004712	-0.415668
8	0	-3.172966	-1.637004	-0.422274
8	0	-1.635365	-3.174534	-0.413757
7	0	-0.732571	2.737096	-0.413969
8	0	-1.929517	3.003521	-0.419157
8	0	0.170853	3.566277	-0.412875
7	0	2.738740	-0.734161	-0.420033
8	0	3.568029	0.169173	-0.424355
8	0	3.005233	-1.931132	-0.422398
17	0	-0.018266	0.010692	2.622351

(5) $Cl^{-}\cdots C_6H_3(CN)_3$

6	0	0.991110	0.989930	-0.554441
6	0	1.341513	-0.358666	-0.555719
6	0	0.363706	-1.351302	-0.552303
6	0	-0.979349	-0.980515	-0.552374
6	0	-1.350114	0.362540	-0.550454
6	0	-0.357465	1.340286	-0.553730
1	0	1.754535	1.753413	-0.527027
1	0	-2.392844	0.641974	-0.518625
1	0	0.643110	-2.394128	-0.522534
6	0	-0.729073	2.727610	-0.560677
7	0	-1.030861	3.855812	-0.609448
6	0	2.728796	-0.730388	-0.564724
7	0	3.856950	-1.032220	-0.614518

6	0	-1.994826	-1.996143	-0.557209
7	0	-2.820597	-2.822036	-0.604921
17	0	-0.024589	-0.006167	2.529755

(6) C_6F_6

6	0	0.000622	1.391104	-0.013912
6	0	-1.202718	0.694612	-0.012927
6	0	-1.201209	-0.695757	-0.012728
6	0	0.003640	-1.389635	-0.013513
6	0	1.206980	-0.693143	-0.014497
6	0	1.205471	0.697226	-0.014697
9	0	0.005084	-2.719586	-0.013507
9	0	-2.352259	-1.361983	-0.011971
9	0	-2.355212	1.358338	-0.012362
9	0	-0.000822	2.721055	-0.014288
9	0	2.356521	1.363452	-0.015824
9	0	2.359473	-1.356869	-0.015434

(7) $C_6H_3(NO_2)_3$

6	0	0.995718	0.995293	-0.416402
6	0	-0.353249	1.318444	-0.416201
6	0	-1.359915	0.364102	-0.416361
6	0	-0.965286	-0.965715	-0.416831
6	0	0.364528	-1.360341	-0.417005
6	0	1.318868	-0.353676	-0.416922
1	0	1.757410	1.756986	-0.416615
1	0	0.643326	-2.400831	-0.417591
1	0	-2.400413	0.642902	-0.416676
7	0	-2.012261	-2.012700	-0.417182

8	0	-3.176458	-1.634177	-0.419242
8	0	-1.633748	-3.176891	-0.415793
7	0	-0.736466	2.748646	-0.415791
8	0	-1.933950	3.002935	-0.416756
8	0	0.173434	3.567607	-0.414814
7	0	2.749066	-0.736898	-0.417445
8	0	3.568031	0.173006	-0.418634
8	0	3.003359	-1.934379	-0.417673

(8) $C_6H_3(CN)_3$

6	0	0.993416	0.991442	-0.560381
6	0	1.346143	-0.360381	-0.561304
6	0	0.364770	-1.354754	-0.559758
6	0	-0.982306	-0.984326	-0.557613
6	0	-1.352758	0.362758	-0.556644
6	0	-0.358412	1.344151	-0.558227
1	0	1.757584	1.755625	-0.561498
1	0	-2.396641	0.642456	-0.554875
1	0	0.644495	-2.398632	-0.560393
6	0	-0.730334	2.732131	-0.557215
7	0	-1.032969	3.860934	-0.556168
6	0	2.734122	-0.732294	-0.563480
7	0	3.862931	-1.034906	-0.565021
6	0	-1.998380	-2.000400	-0.555965
7	0	-2.824873	-2.826652	-0.554401
